# Supplementary material for: Using Zebrafish to Screen Developmental Toxicity of Per- and Polyfluoroalkyl Substances (PFAS)
Source: Toxics. 2024 Jul 10;12(7):501. doi: 10.3390/toxics12070501 (PMC11281043; doi:10.3390/toxics12070501)
Supplement: Supplementary file 1 [file toxics-12-00501-s001.zip › Supplemental Figure S1 BMC Curves 12-05-2023.pdf]

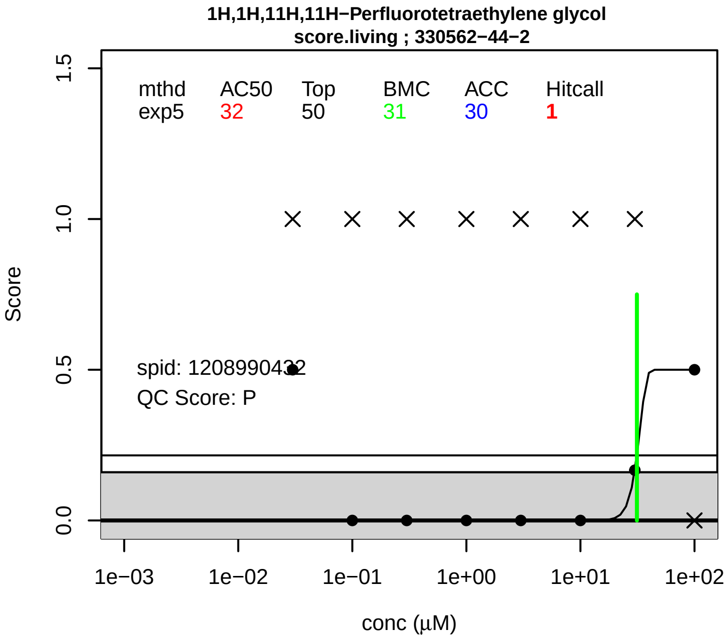
1H,1H,11H,11H−Perfluorotetraethylene glycol
score.living ; 330562−44−2
mthd exp5
AC50 32
Top 50
BMC 31
ACC 30
Hitcall 1
spid: 1208990432
QC Score: P
Score
conc (µM)

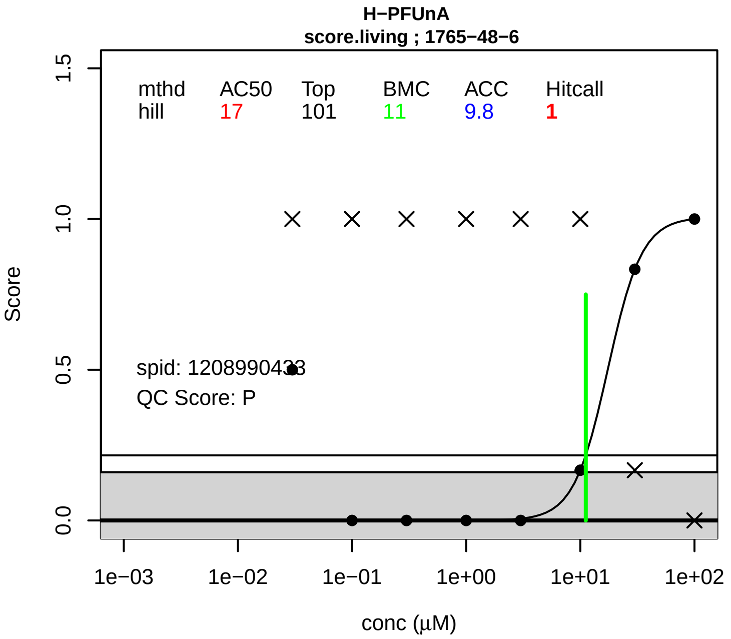
H−PFUnA
score.living ; 1765−48−6
mthd hill
AC50 17
Top 101
BMC 11
ACC 9.8
Hitcall 1
spid: 1208990433
QC Score: P
Score
conc (µM)

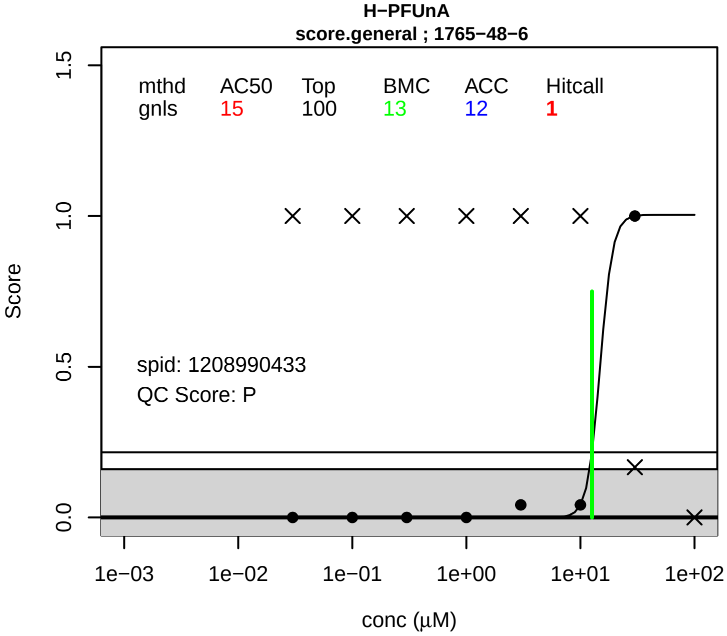
H−PFUnA
score.general ; 1765−48−6
mthd gnls
AC50 15
Top 100
BMC 13
ACC 12
Hitcall 1
spid: 1208990433
QC Score: P
Score
conc (µM)

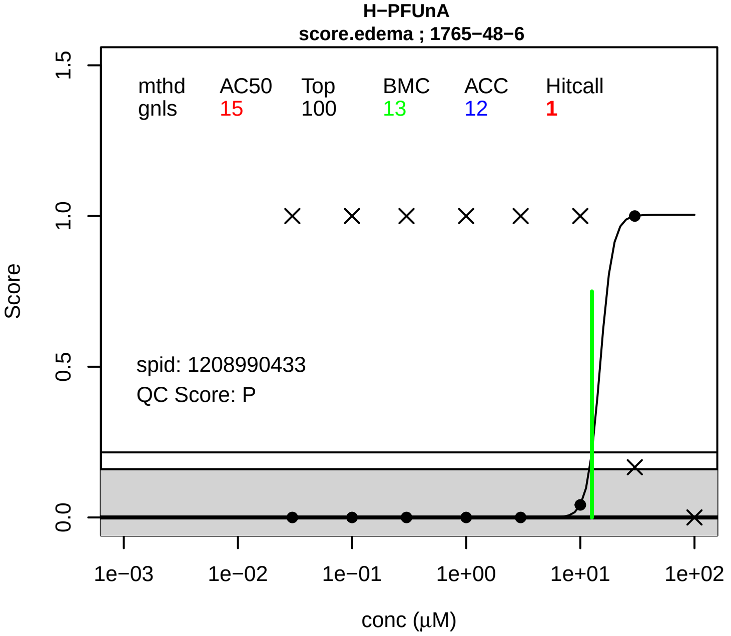
H−PFUnA
score.edema ; 1765−48−6
mthd gnls
AC50 15
Top 100
BMC 13
ACC 12
Hitcall 1
spid: 1208990433
QC Score: P
Score
conc (µM)

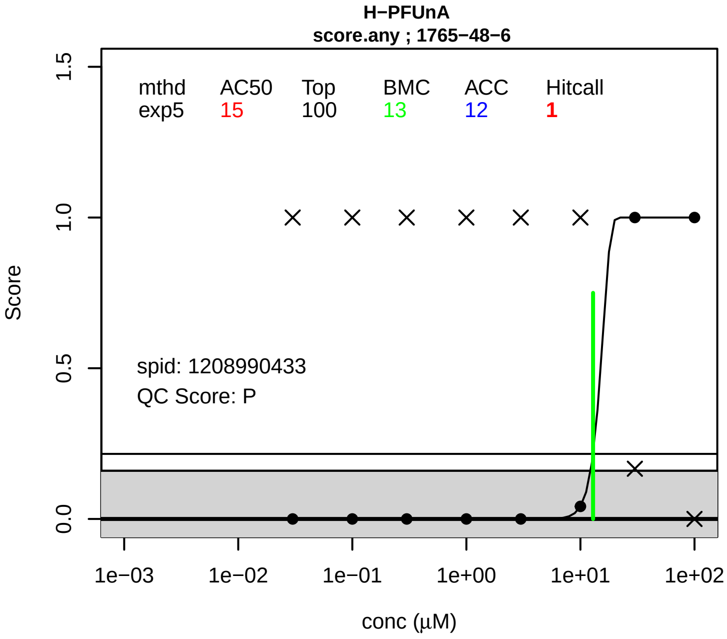
H−PFUnA
score.any ; 1765−48−6
mthd exp5
AC50 15
Top 100
BMC 13
ACC 12
Hitcall 1
spid: 1208990433
QC Score: P
Score
conc (µM)

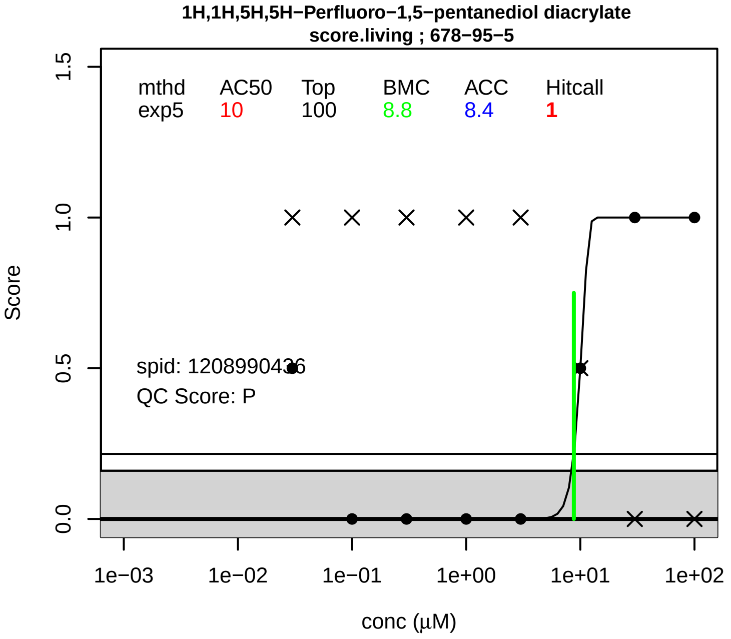
1H,1H,5H,5H−Perfluoro−1,5−pentanediol diacrylate
score.living ; 678−95−5
mthd exp5
AC50 10
Top 100
BMC 8.8
ACC 8.4
Hitcall 1
spid: 1208990436
QC Score: P
Score
conc (µM)

1H,1H,5H,5H−Perfluoro−1,5−pentanediol diacrylate
score.any ; 678−95−5

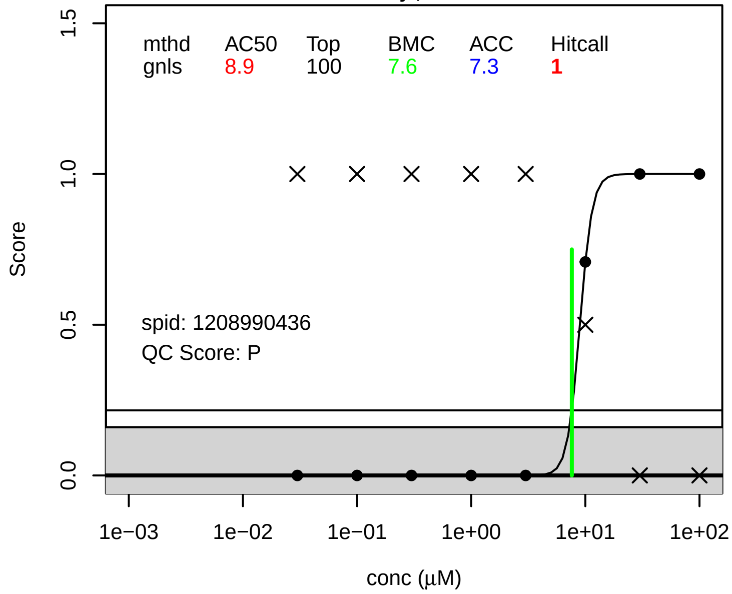

2,2,3,3−Tetrafluoropropyl acrylate
score.living ; 7383−71−3

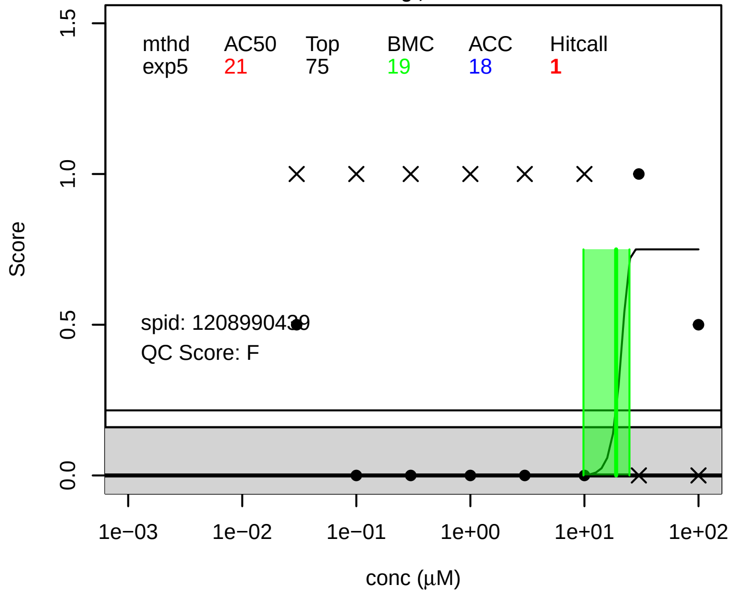

2,2,3,3−Tetrafluoropropyl acrylate
score.general ; 7383−71−3

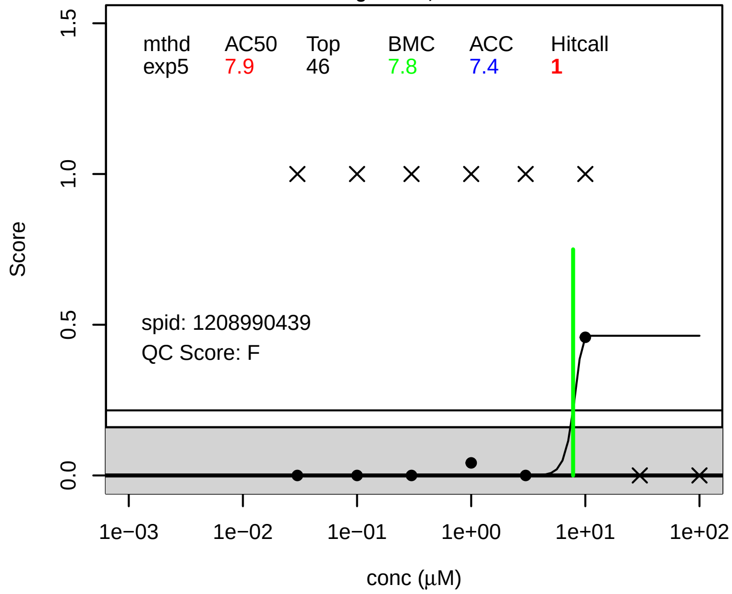

2,2,3,3−Tetrafluoropropyl acrylate
score.edema ; 7383−71−3

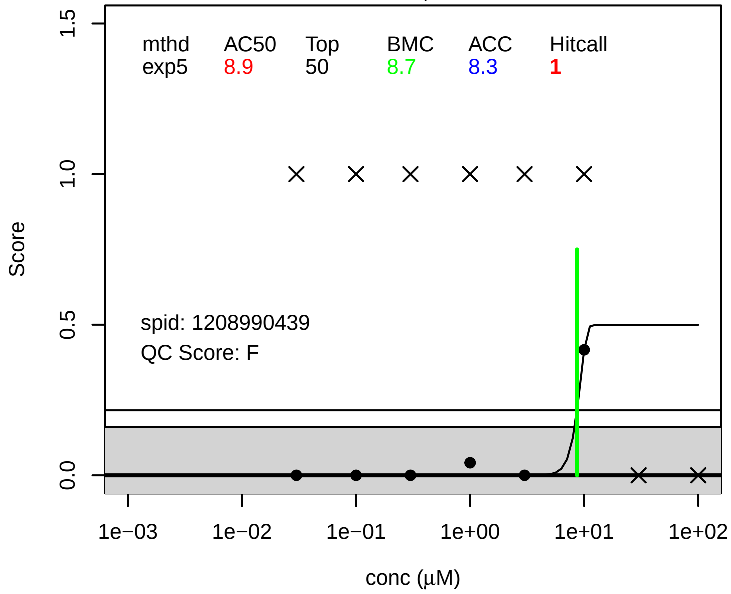

2,2,3,3−Tetrafluoropropyl acrylate
score.any ; 7383−71−3

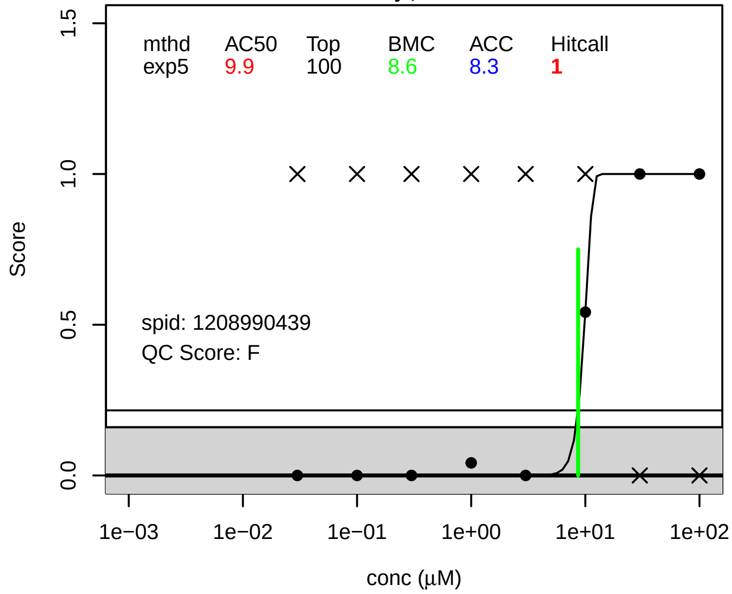

1H,1H,7H−Perfluoroheptyl 4−methylbenzenesulfonate
score.general ; 424−16−8

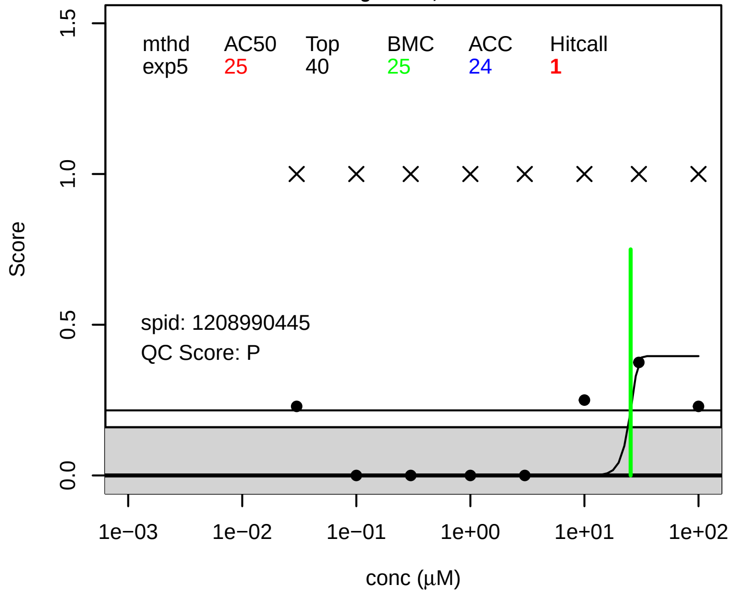

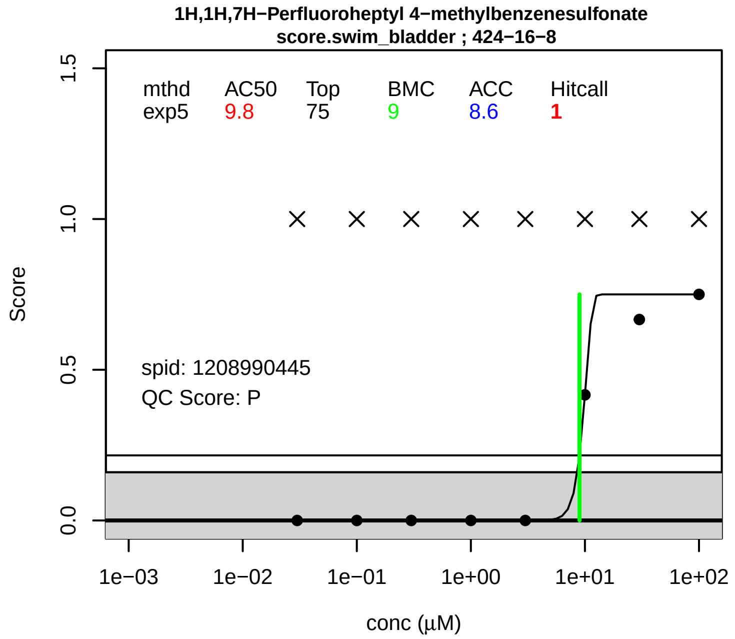
1H,1H,7H−Perfluoroheptyl 4−methylbenzenesulfonate
score.swim_bladder ; 424−16−8
mthd AC50 Top BMC ACC Hitcall
exp5 9.8 75 9 8.6 1
spid: 1208990445
QC Score: P
Score
conc (µM)

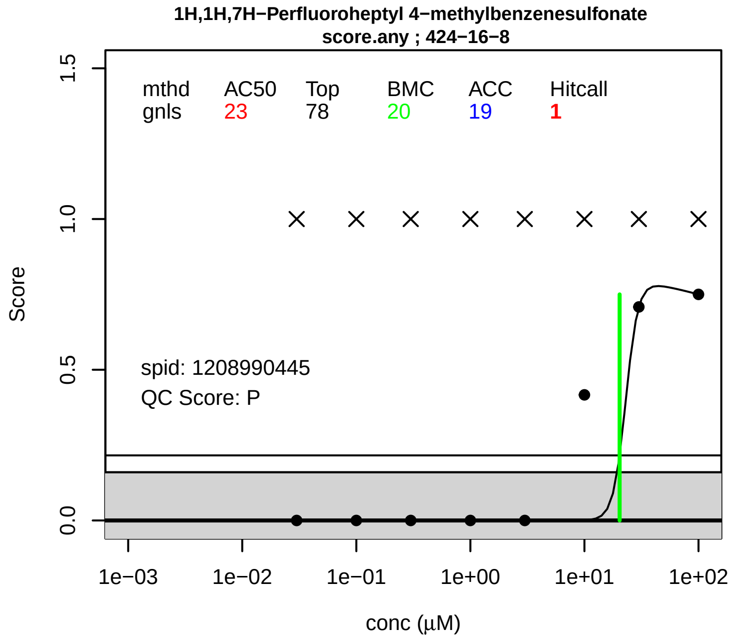
1H,1H,7H−Perfluoroheptyl 4−methylbenzenesulfonate
score.any ; 424−16−8
mthd AC50 Top BMC ACC Hitcall
gnls 23 78 20 19 1
spid: 1208990445
QC Score: P
Score
conc (µM)

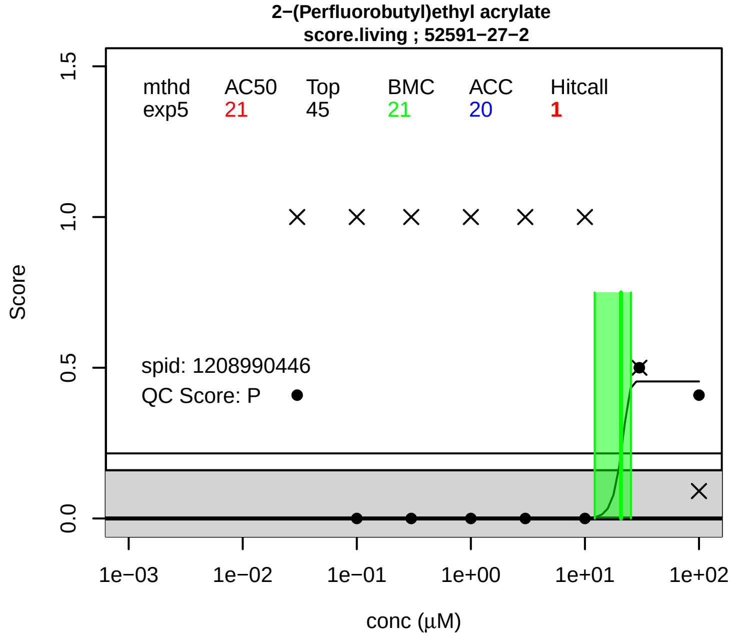
2−(Perfluorobutyl)ethyl acrylate
score.living ; 52591−27−2
mthd AC50 Top BMC ACC Hitcall
exp5 21 45 21 20 1
spid: 1208990446
QC Score: P
Score
conc (µM)

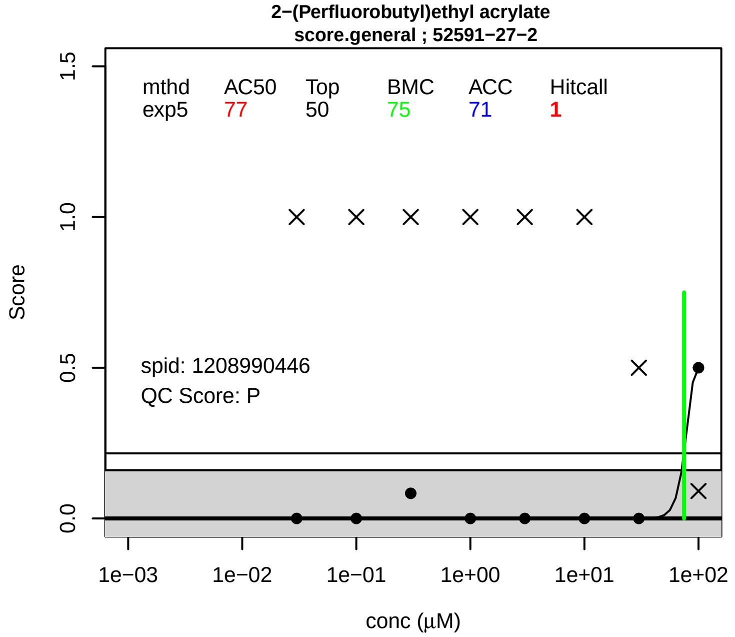
2−(Perfluorobutyl)ethyl acrylate
score.general ; 52591−27−2
mthd AC50 Top BMC ACC Hitcall
exp5 77 50 75 71 1
spid: 1208990446
QC Score: P
Score
conc (µM)

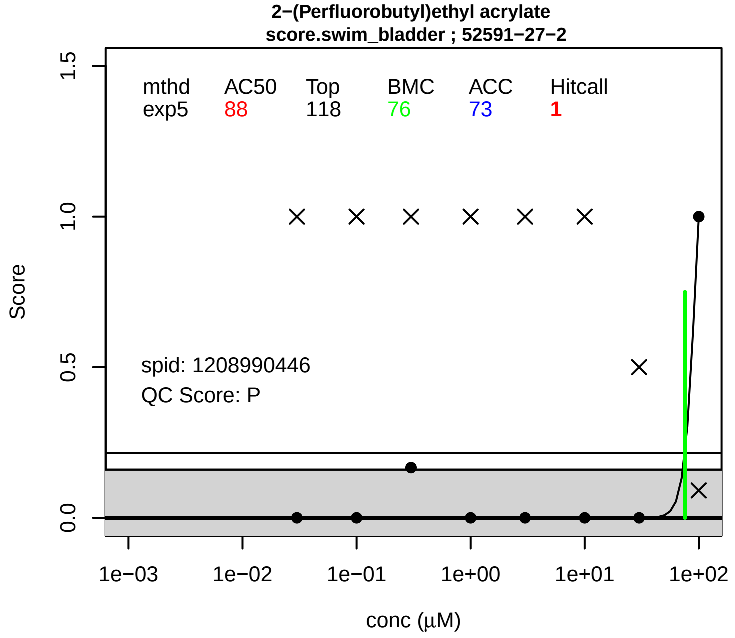
2−(Perfluorobutyl)ethyl acrylate
score.swim_bladder ; 52591−27−2
mthd AC50 Top BMC ACC Hitcall
exp5 88 118 76 73 1
spid: 1208990446
QC Score: P
Score
conc (µM)

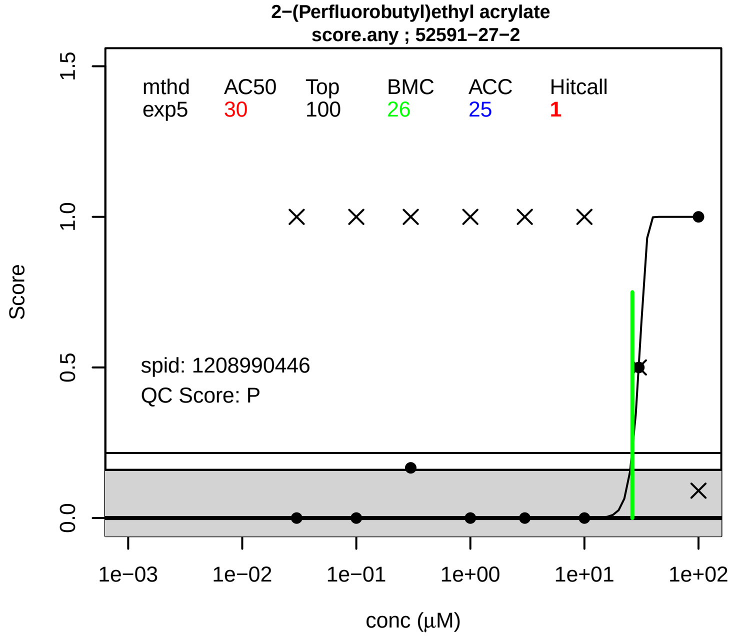
2−(Perfluorobutyl)ethyl acrylate
score.any ; 52591−27−2
mthd AC50 Top BMC ACC Hitcall
exp5 30 100 26 25 1
spid: 1208990446
QC Score: P
Score
conc (µM)

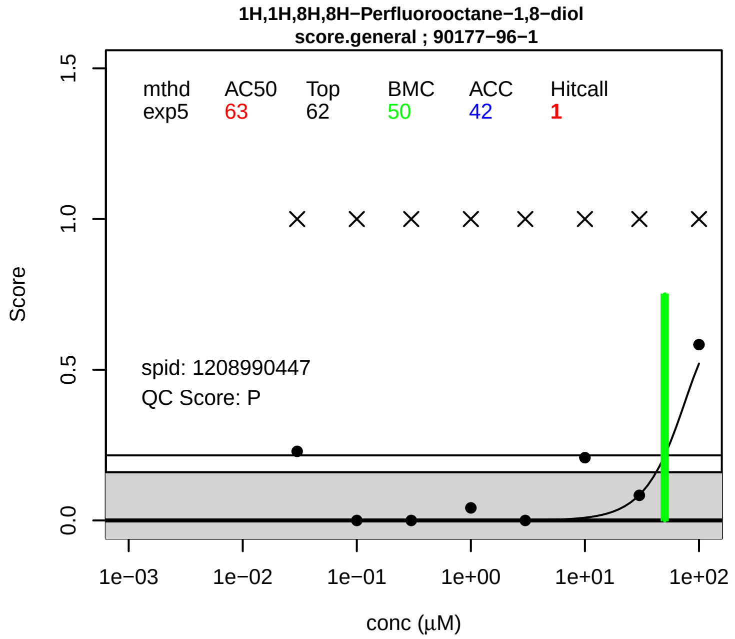
1H,1H,8H,8H−Perfluorooctane−1,8−diol
score.general ; 90177−96−1
mthd AC50 Top BMC ACC Hitcall
exp5 63 62 50 42 1
spid: 1208990447
QC Score: P
Score
conc (μM)

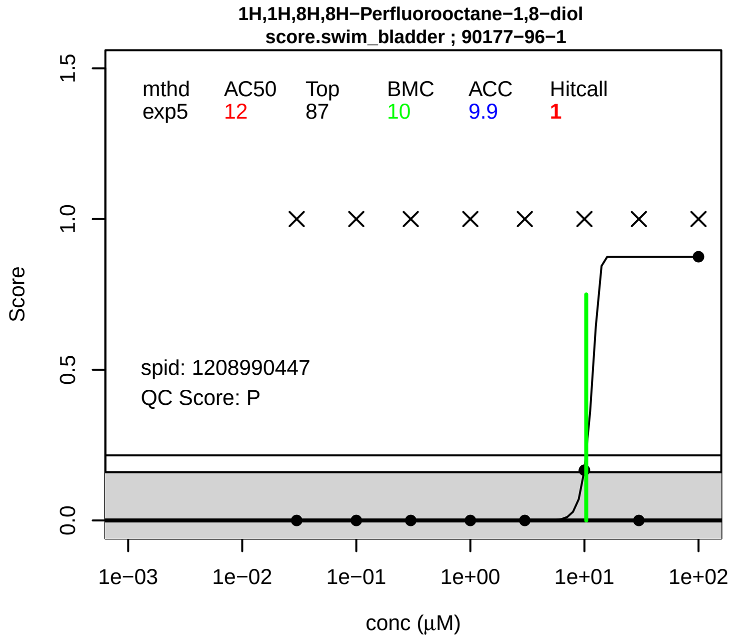
1H,1H,8H,8H−Perfluorooctane−1,8−diol
score.swim_bladder ; 90177−96−1
mthd AC50 Top BMC ACC Hitcall
exp5 12 87 10 9.9 1
spid: 1208990447
QC Score: P
Score
conc (μM)

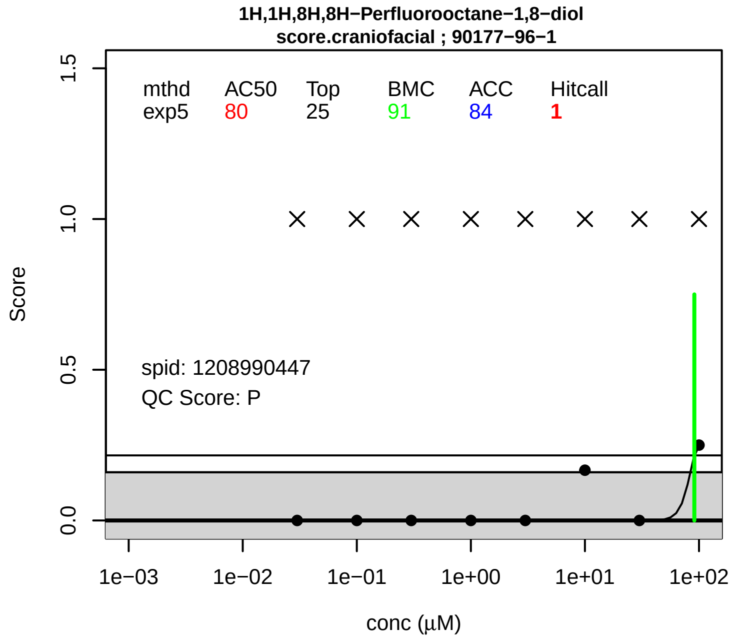
1H,1H,8H,8H−Perfluorooctane−1,8−diol
score.craniofacial ; 90177−96−1
mthd AC50 Top BMC ACC Hitcall
exp5 80 25 91 84 1
spid: 1208990447
QC Score: P
Score
conc (μM)

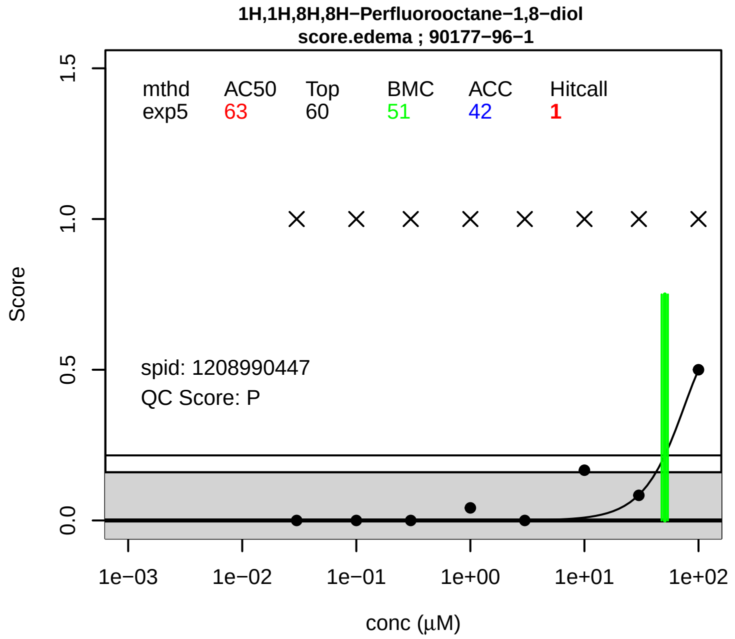
1H,1H,8H,8H−Perfluorooctane−1,8−diol
score.edema ; 90177−96−1
mthd AC50 Top BMC ACC Hitcall
exp5 63 60 51 42 1
spid: 1208990447
QC Score: P
Score
conc (μM)

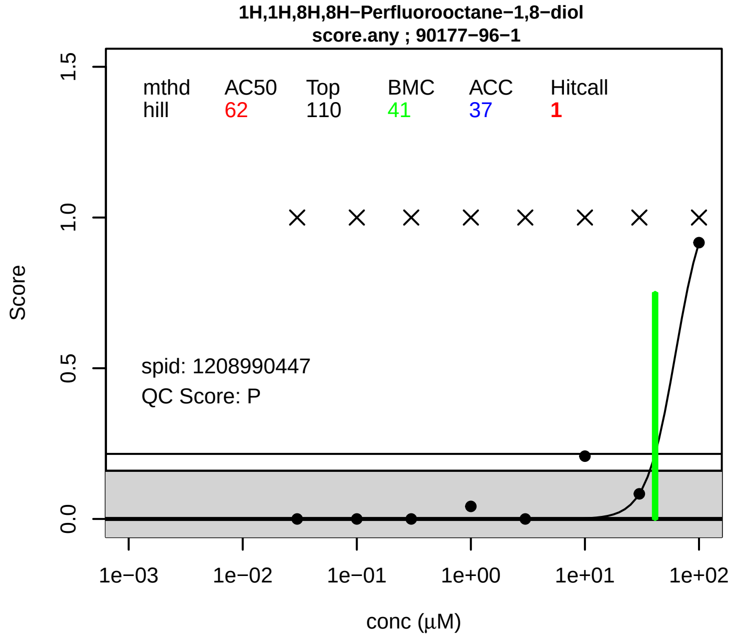
1H,1H,8H,8H−Perfluorooctane−1,8−diol
score.any ; 90177−96−1
mthd AC50 Top BMC ACC Hitcall
hill 62 110 41 37 1
spid: 1208990447
QC Score: P
Score
conc (μM)

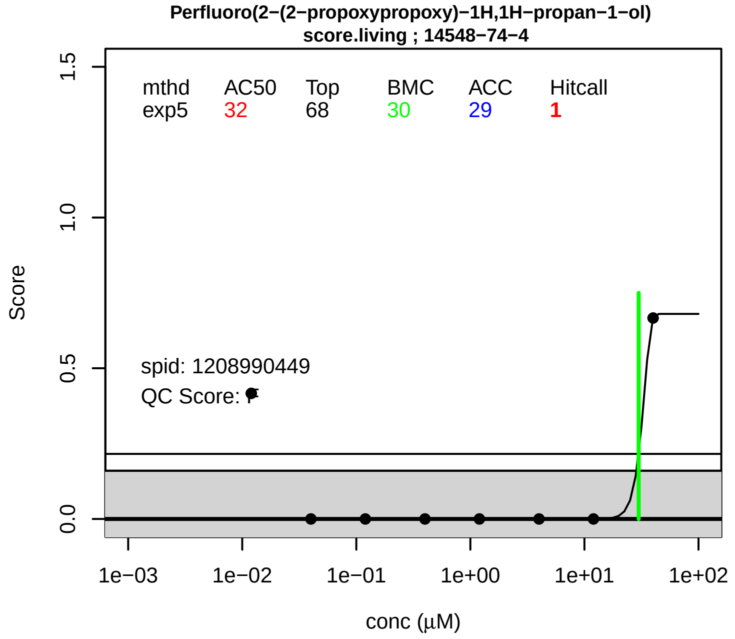
Perfluoro(2−(2−propoxypropoxy)−1H,1H−propan−1−ol)
score.living ; 14548−74−4
mthd AC50 Top BMC ACC Hitcall
exp5 32 68 30 29 1
spid: 1208990449
QC Score:
Score
conc (μM)

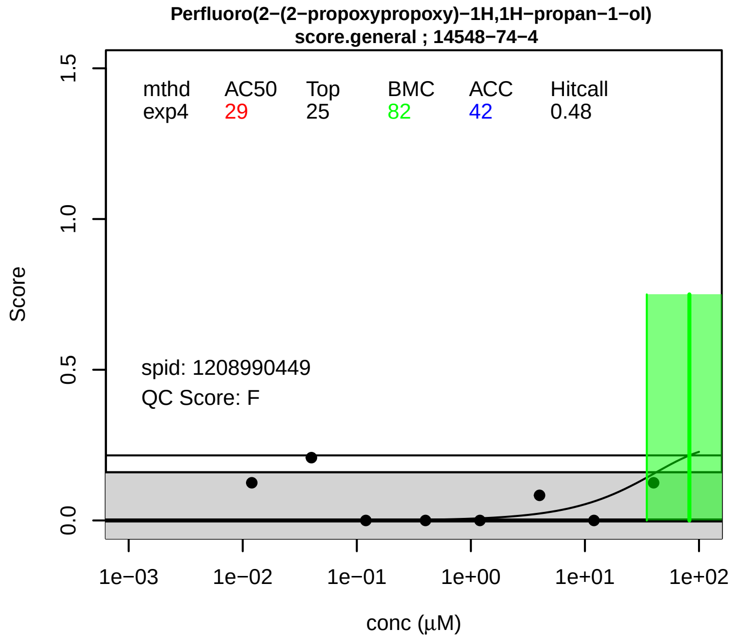
Perfluoro(2−(2−propoxypropoxy)−1H,1H−propan−1−ol)
score.general ; 14548−74−4
mthd AC50 Top BMC ACC Hitcall
exp4 29 25 82 42 0.48
spid: 1208990449
QC Score: F
Score
conc (µM)
0.0 0.5 1.0 1.5
1e−03 1e−02 1e−01 1e+00 1e+01 1e+02

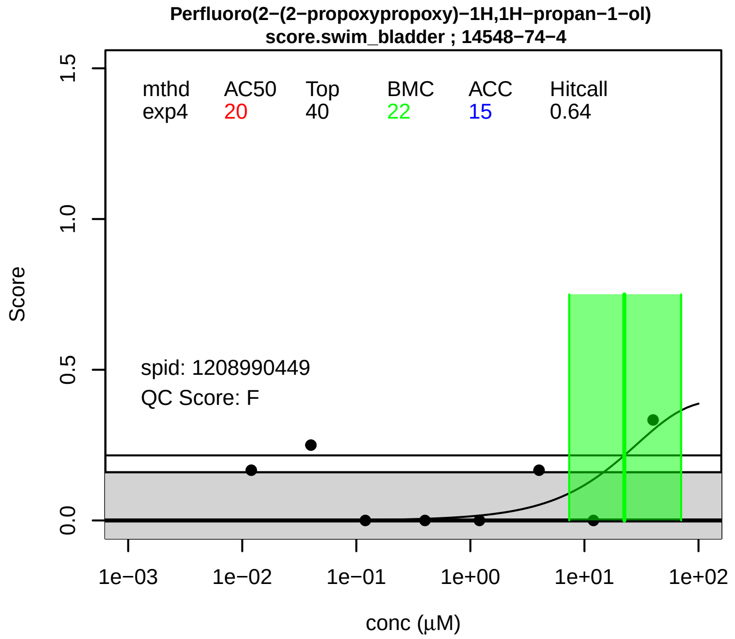
Perfluoro(2−(2−propoxypropoxy)−1H,1H−propan−1−ol)
score.swim_bladder ; 14548−74−4
mthd AC50 Top BMC ACC Hitcall
exp4 20 40 22 15 0.64
spid: 1208990449
QC Score: F
Score
conc (µM)
0.0 0.5 1.0 1.5
1e−03 1e−02 1e−01 1e+00 1e+01 1e+02

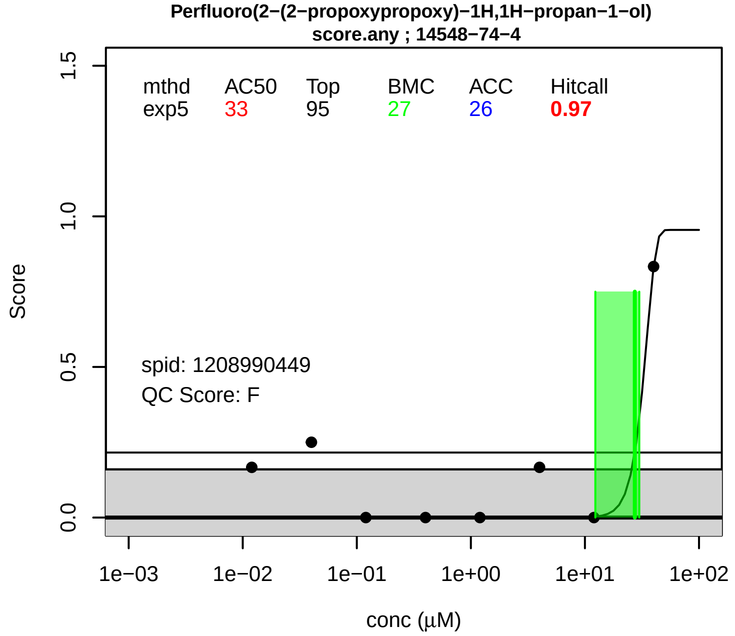
Perfluoro(2−(2−propoxypropoxy)−1H,1H−propan−1−ol)
score.any ; 14548−74−4
mthd AC50 Top BMC ACC Hitcall
exp5 33 95 27 26 0.97
spid: 1208990449
QC Score: F
Score
conc (µM)
0.0 0.5 1.0 1.5
1e−03 1e−02 1e−01 1e+00 1e+01 1e+02

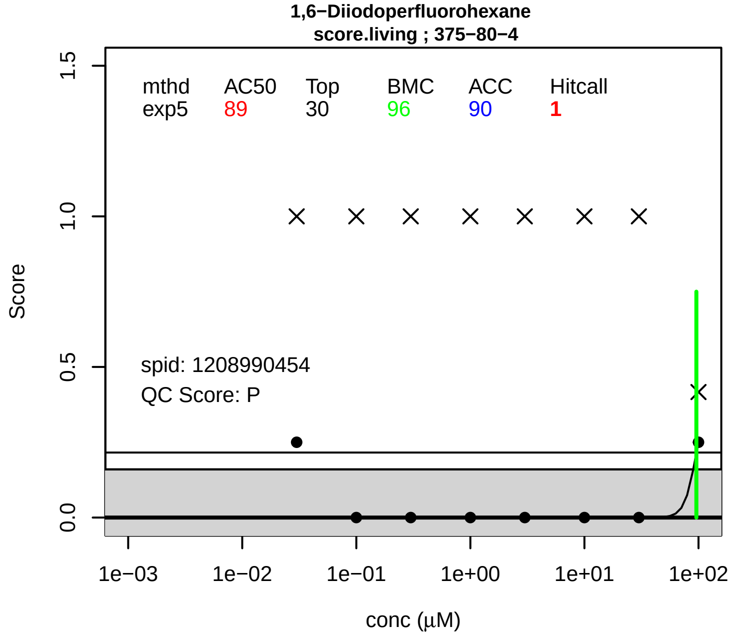
1,6−Diiodoperfluorohexane
score.living ; 375−80−4
mthd AC50 Top BMC ACC Hitcall
exp5 89 30 96 90 1
spid: 1208990454
QC Score: P
Score
conc (µM)
0.0 0.5 1.0 1.5
1e−03 1e−02 1e−01 1e+00 1e+01 1e+02

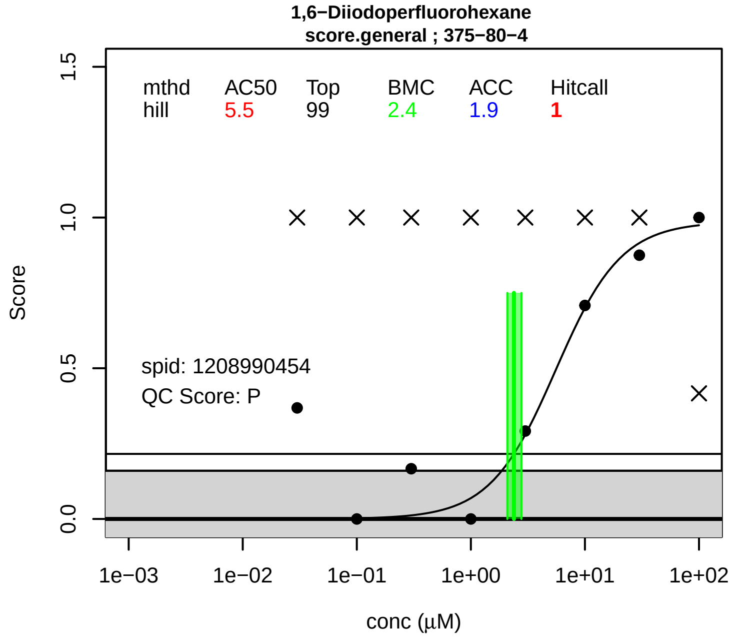
1,6−Diiodoperfluorohexane
score.general ; 375−80−4
mthd AC50 Top BMC ACC Hitcall
hill 5.5 99 2.4 1.9 1
spid: 1208990454
QC Score: P
Score
conc (µM)
0.0 0.5 1.0 1.5
1e−03 1e−02 1e−01 1e+00 1e+01 1e+02

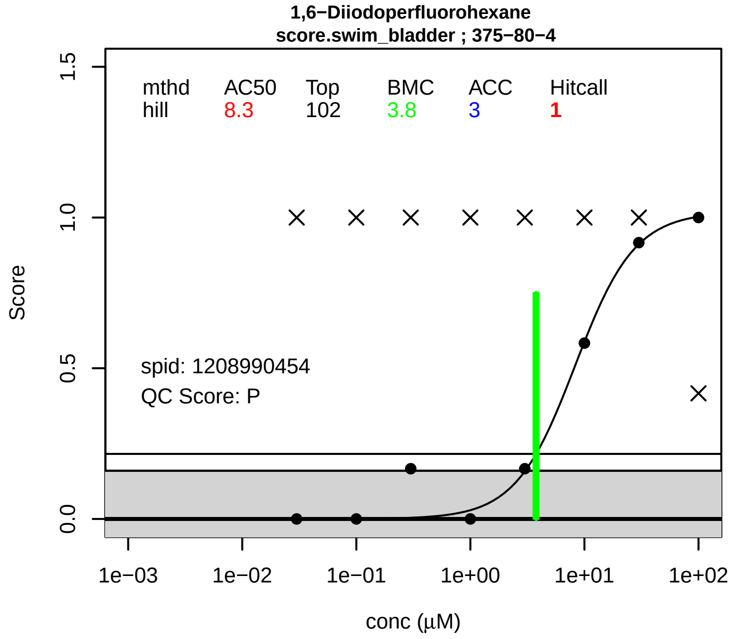
1,6−Diiodoperfluorohexane
score.swim_bladder ; 375−80−4
mthd AC50 Top BMC ACC Hitcall
hill 8.3 102 3.8 3 1
spid: 1208990454
QC Score: P
Score
conc (µM)
0.0 0.5 1.0 1.5
1e−03 1e−02 1e−01 1e+00 1e+01 1e+02

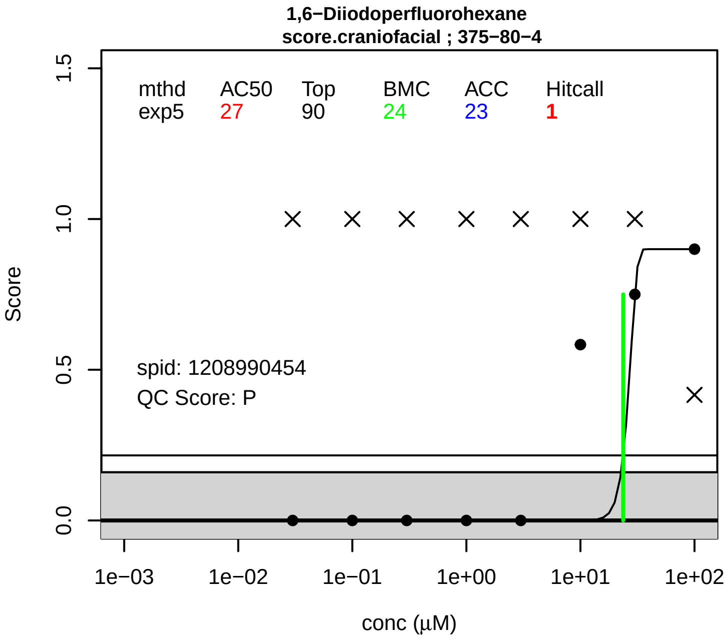
1,6−Diiodoperfluorohexane
score.craniofacial ; 375−80−4
mthd exp5
AC50 27
Top 90
BMC 24
ACC 23
Hitcall 1
spid: 1208990454
QC Score: P
Score
conc (µM)
0.0 0.5 1.0 1.5
1e−03 1e−02 1e−01 1e+00 1e+01 1e+02

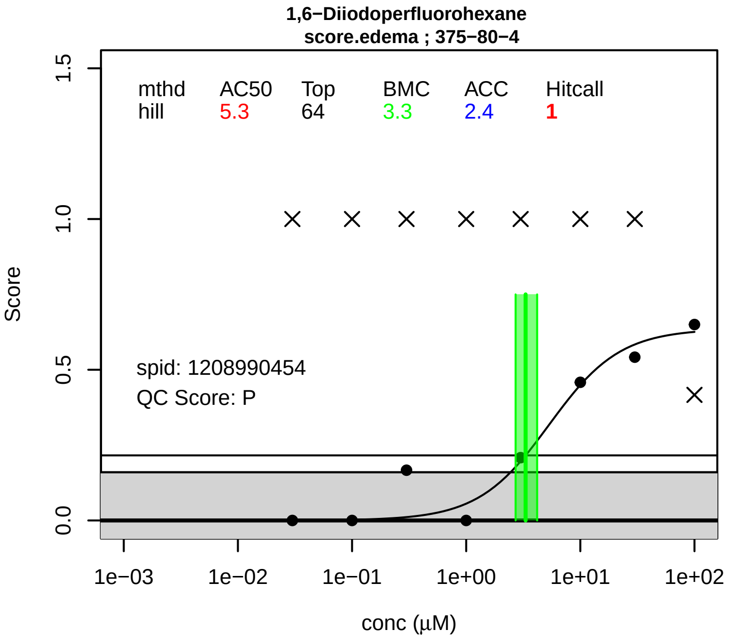
1,6−Diiodoperfluorohexane
score.edema ; 375−80−4
mthd hill
AC50 5.3
Top 64
BMC 3.3
ACC 2.4
Hitcall 1
spid: 1208990454
QC Score: P
Score
conc (µM)
0.0 0.5 1.0 1.5
1e−03 1e−02 1e−01 1e+00 1e+01 1e+02

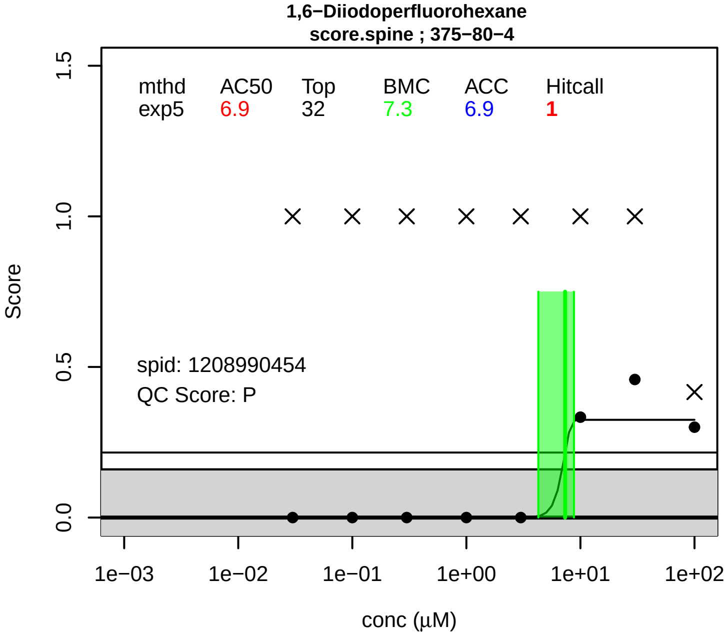
1,6−Diiodoperfluorohexane
score.spine ; 375−80−4
mthd exp5
AC50 6.9
Top 32
BMC 7.3
ACC 6.9
Hitcall 1
spid: 1208990454
QC Score: P
Score
conc (µM)
0.0 0.5 1.0 1.5
1e−03 1e−02 1e−01 1e+00 1e+01 1e+02

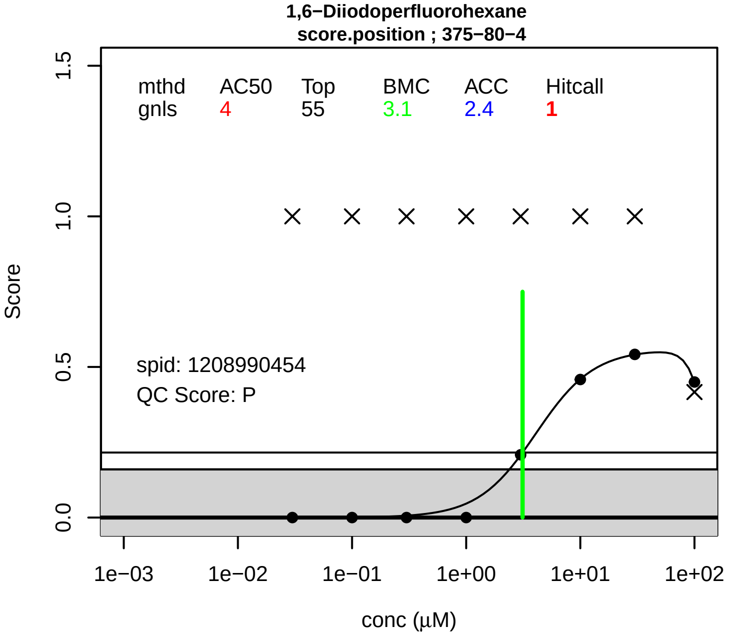
1,6−Diiodoperfluorohexane
score.position ; 375−80−4
mthd gnls
AC50 4
Top 55
BMC 3.1
ACC 2.4
Hitcall 1
spid: 1208990454
QC Score: P
Score
conc (µM)
0.0 0.5 1.0 1.5
1e−03 1e−02 1e−01 1e+00 1e+01 1e+02

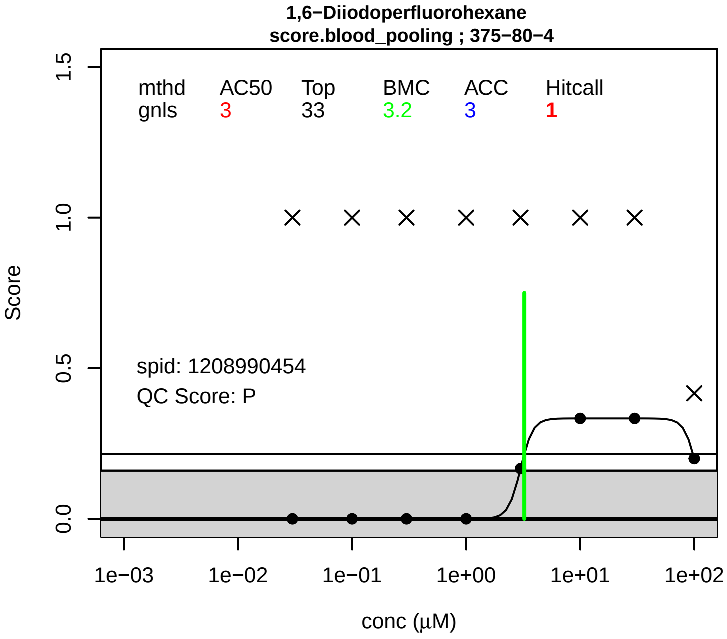
1,6−Diiodoperfluorohexane
score.blood_pooling ; 375−80−4
mthd gnls
AC50 3
Top 33
BMC 3.2
ACC 3
Hitcall 1
spid: 1208990454
QC Score: P
Score
conc (µM)
0.0 0.5 1.0 1.5
1e−03 1e−02 1e−01 1e+00 1e+01 1e+02

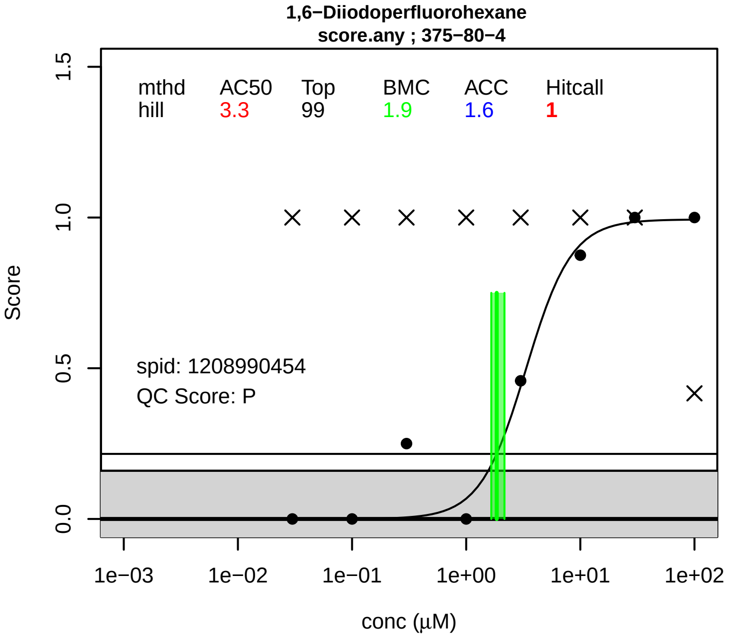
1,6−Diiodoperfluorohexane
score.any ; 375−80−4
mthd hill
AC50 3.3
Top 99
BMC 1.9
ACC 1.6
Hitcall 1
spid: 1208990454
QC Score: P
Score
conc (µM)
0.0 0.5 1.0 1.5
1e−03 1e−02 1e−01 1e+00 1e+01 1e+02

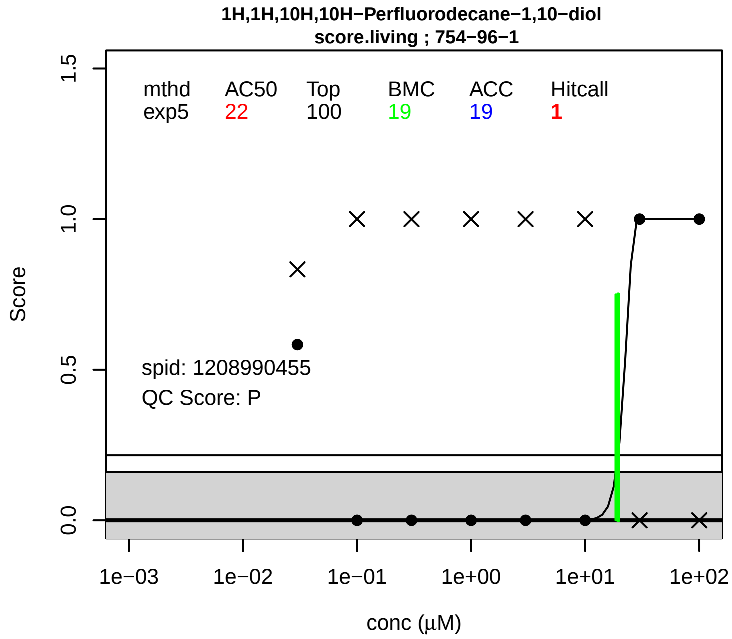
1H,1H,10H,10H−Perfluorodecane−1,10−diol
score.living ; 754−96−1
mthd AC50 Top BMC ACC Hitcall
exp5 22 100 19 19 1
spid: 1208990455
QC Score: P
Score
conc (µM)

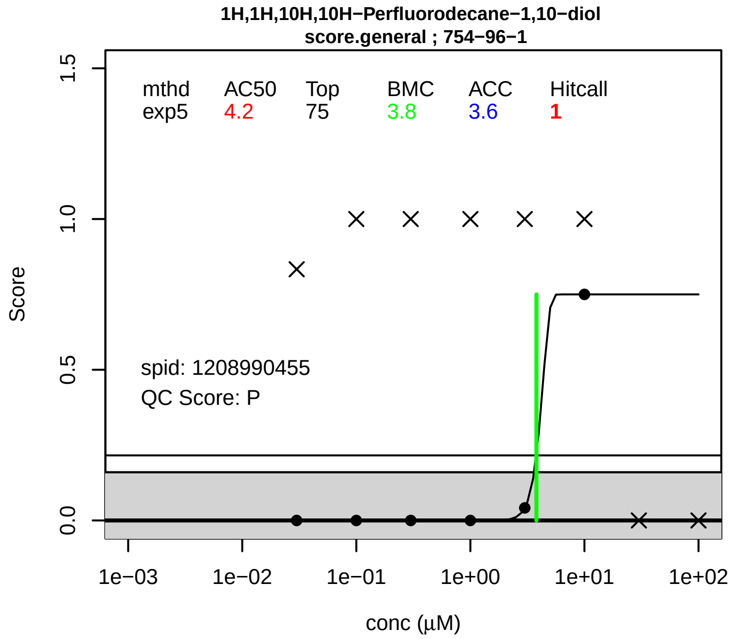
1H,1H,10H,10H−Perfluorodecane−1,10−diol
score.general ; 754−96−1
mthd AC50 Top BMC ACC Hitcall
exp5 4.2 75 3.8 3.6 1
spid: 1208990455
QC Score: P
Score
conc (µM)

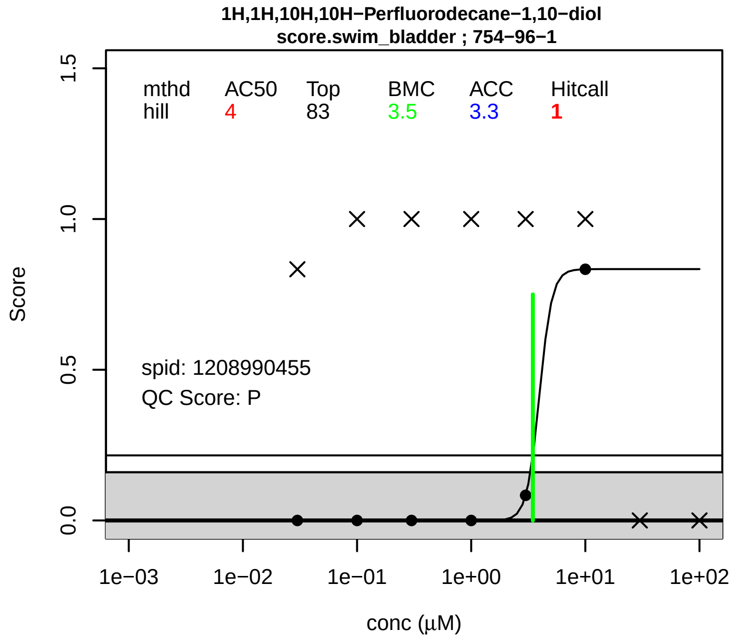
1H,1H,10H,10H−Perfluorodecane−1,10−diol
score.swim_bladder ; 754−96−1
mthd AC50 Top BMC ACC Hitcall
hill 4 83 3.5 3.3 1
spid: 1208990455
QC Score: P
Score
conc (µM)

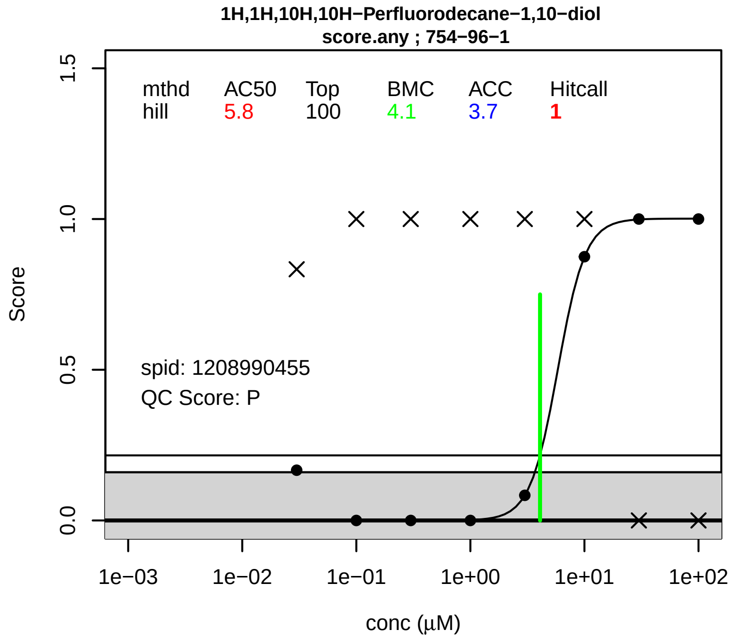
1H,1H,10H,10H−Perfluorodecane−1,10−diol
score.any ; 754−96−1
mthd AC50 Top BMC ACC Hitcall
hill 5.8 100 4.1 3.7 1
spid: 1208990455
QC Score: P
Score
conc (µM)

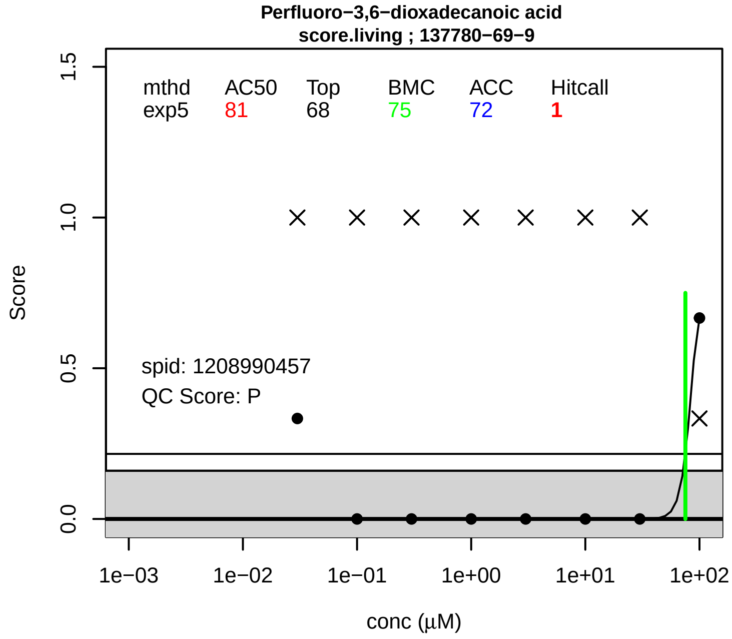
Perfluoro−3,6−dioxadecanoic acid
score.living ; 137780−69−9
mthd AC50 Top BMC ACC Hitcall
exp5 81 68 75 72 1
spid: 1208990457
QC Score: P
Score
conc (µM)

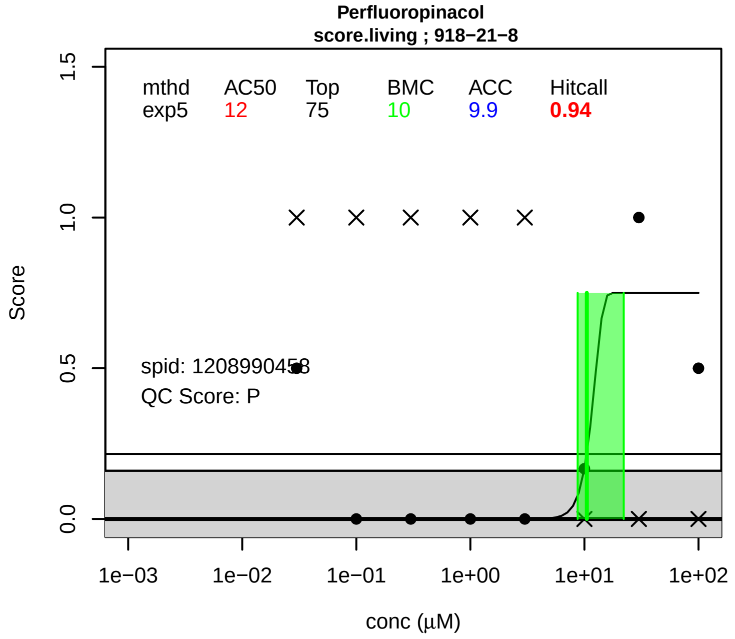
Perfluoropinacol
score.living ; 918−21−8
mthd AC50 Top BMC ACC Hitcall
exp5 12 75 10 9.9 0.94
QC Score: P
Score
conc (µM)

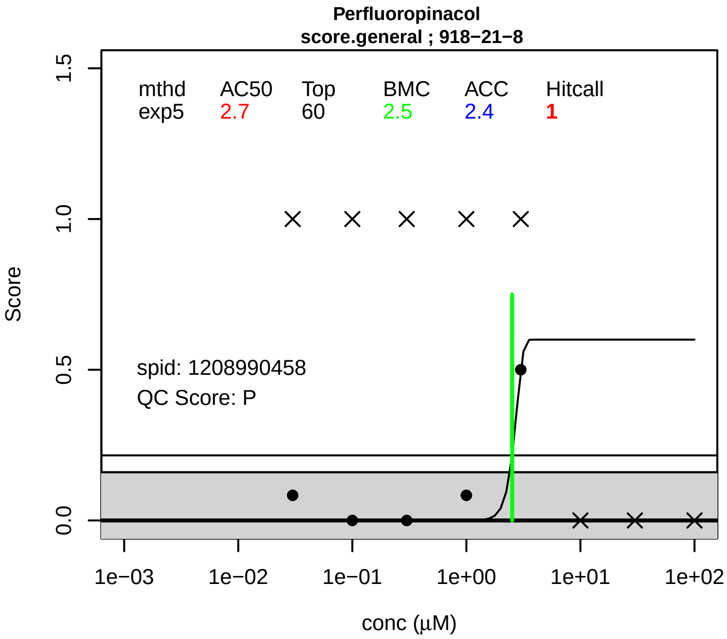
Perfluoropinacol
score.general ; 918−21−8
mthd AC50 Top BMC ACC Hitcall
exp5 2.7 60 2.5 2.4 1
spid: 1208990458
QC Score: P
Score
conc (µM)
1e−03 1e−02 1e−01 1e+00 1e+01 1e+02
0.0 0.5 1.0 1.5

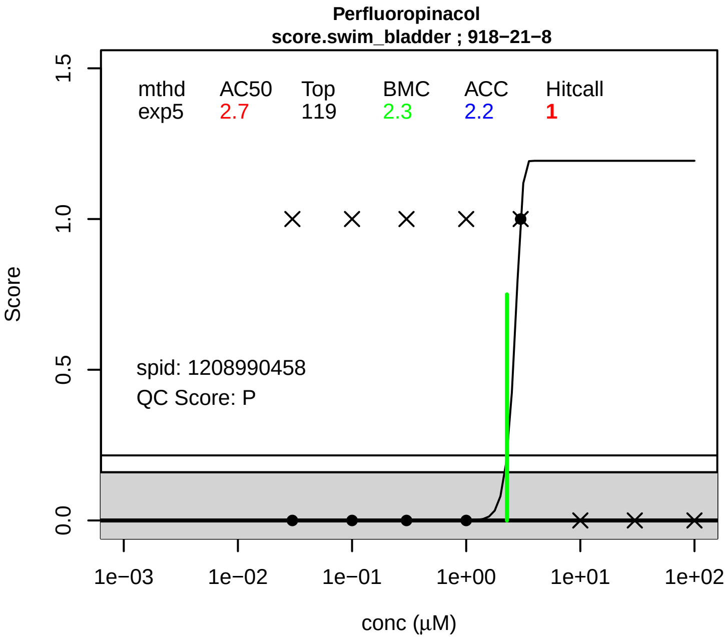
Perfluoropinacol
score.swim_bladder ; 918−21−8
mthd AC50 Top BMC ACC Hitcall
exp5 2.7 119 2.3 2.2 1
spid: 1208990458
QC Score: P
Score
conc (µM)
1e−03 1e−02 1e−01 1e+00 1e+01 1e+02
0.0 0.5 1.0 1.5

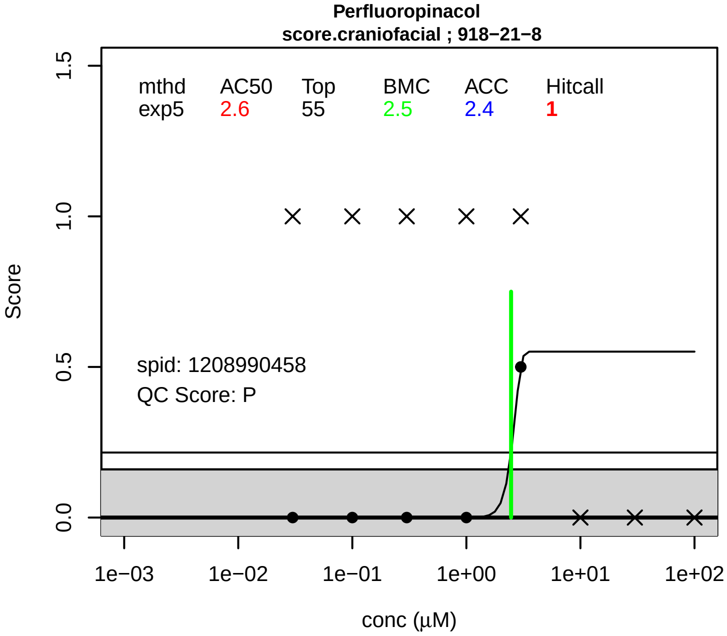
Perfluoropinacol
score.craniofacial ; 918−21−8
mthd AC50 Top BMC ACC Hitcall
exp5 2.6 55 2.5 2.4 1
spid: 1208990458
QC Score: P
Score
conc (µM)
1e−03 1e−02 1e−01 1e+00 1e+01 1e+02
0.0 0.5 1.0 1.5

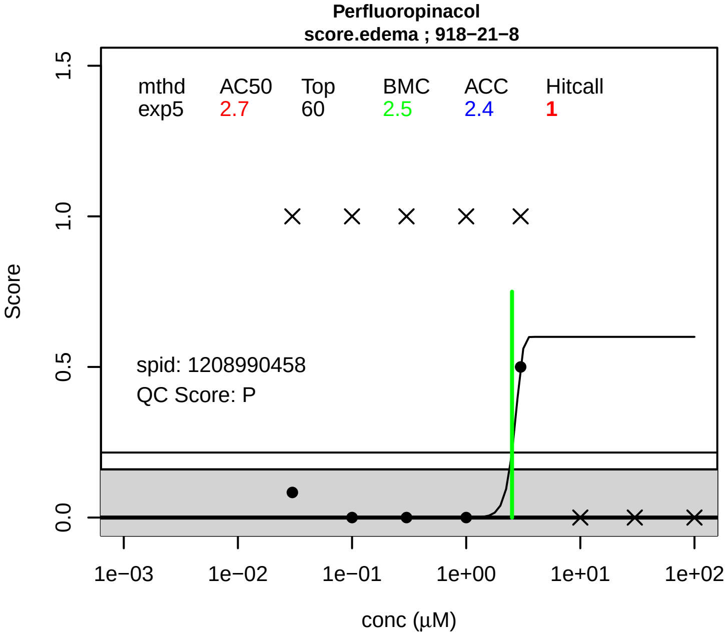
Perfluoropinacol
score.edema ; 918−21−8
mthd AC50 Top BMC ACC Hitcall
exp5 2.7 60 2.5 2.4 1
spid: 1208990458
QC Score: P
Score
conc (µM)
1e−03 1e−02 1e−01 1e+00 1e+01 1e+02
0.0 0.5 1.0 1.5

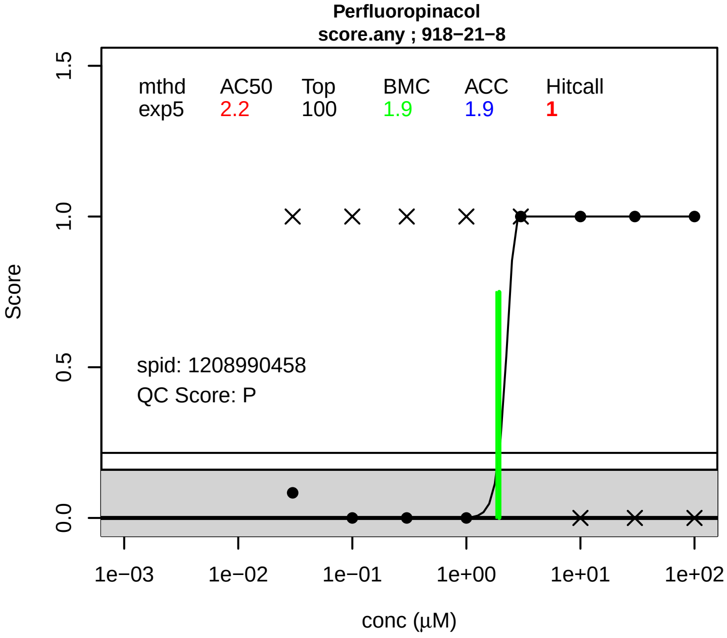
Perfluoropinacol
score.any ; 918−21−8
mthd AC50 Top BMC ACC Hitcall
exp5 2.2 100 1.9 1.9 1
spid: 1208990458
QC Score: P
Score
conc (µM)
1e−03 1e−02 1e−01 1e+00 1e+01 1e+02
0.0 0.5 1.0 1.5

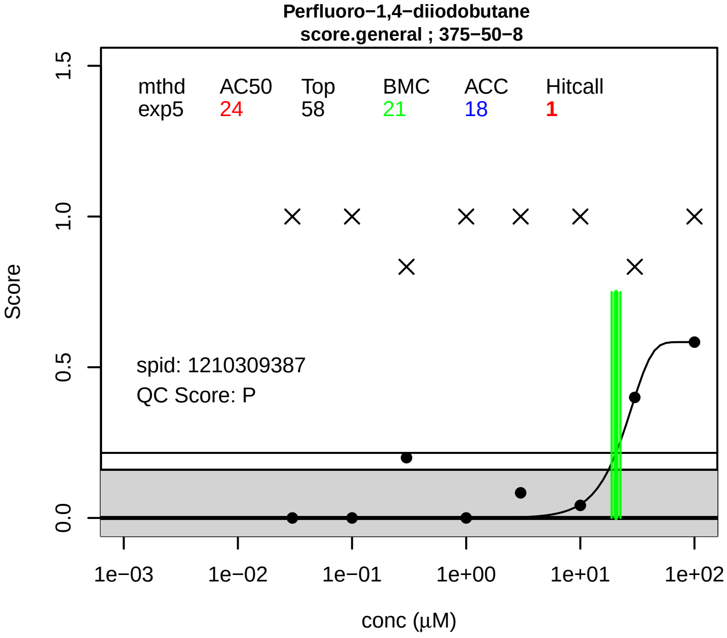
Perfluoro−1,4−diiodobutane
score.general ; 375−50−8
mthd AC50 Top BMC ACC Hitcall
exp5 24 58 21 18 1
spid: 1210309387
QC Score: P
Score
conc (µM)
1e−03 1e−02 1e−01 1e+00 1e+01 1e+02
0.0 0.5 1.0 1.5

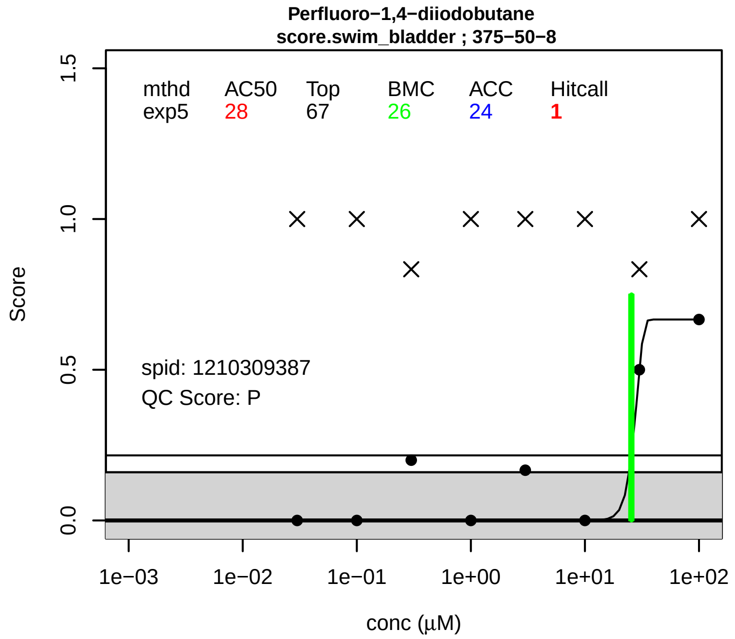
Perfluoro−1,4−diiodobutane
score.swim_bladder ; 375−50−8
mthd AC50 Top BMC ACC Hitcall
exp5 28 67 26 24 1
spid: 1210309387
QC Score: P
Score
conc (µM)

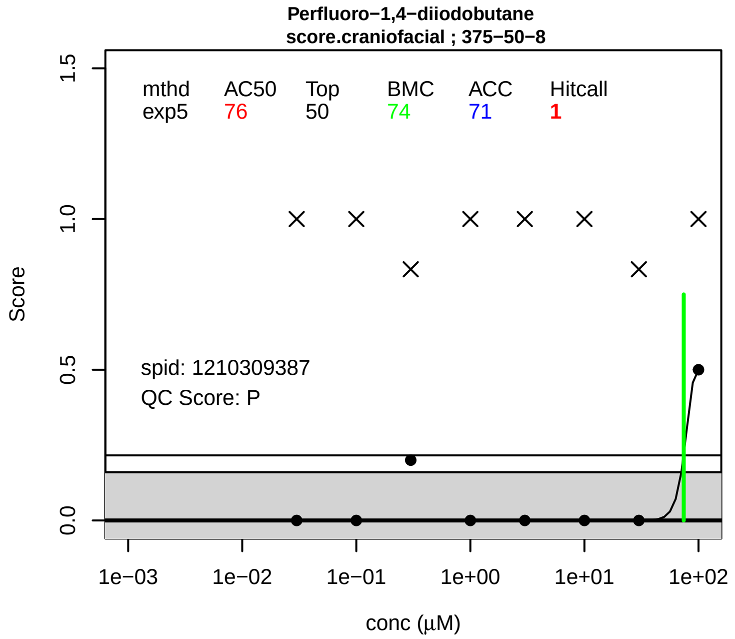
Perfluoro−1,4−diiodobutane
score.craniofacial ; 375−50−8
mthd AC50 Top BMC ACC Hitcall
exp5 76 50 74 71 1
spid: 1210309387
QC Score: P
Score
conc (µM)

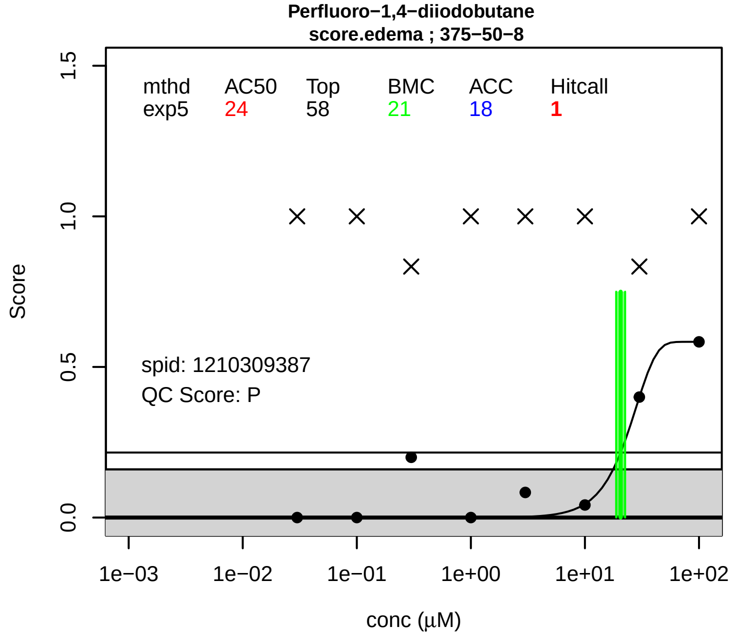
Perfluoro−1,4−diiodobutane
score.edema ; 375−50−8
mthd AC50 Top BMC ACC Hitcall
exp5 24 58 21 18 1
spid: 1210309387
QC Score: P
Score
conc (µM)

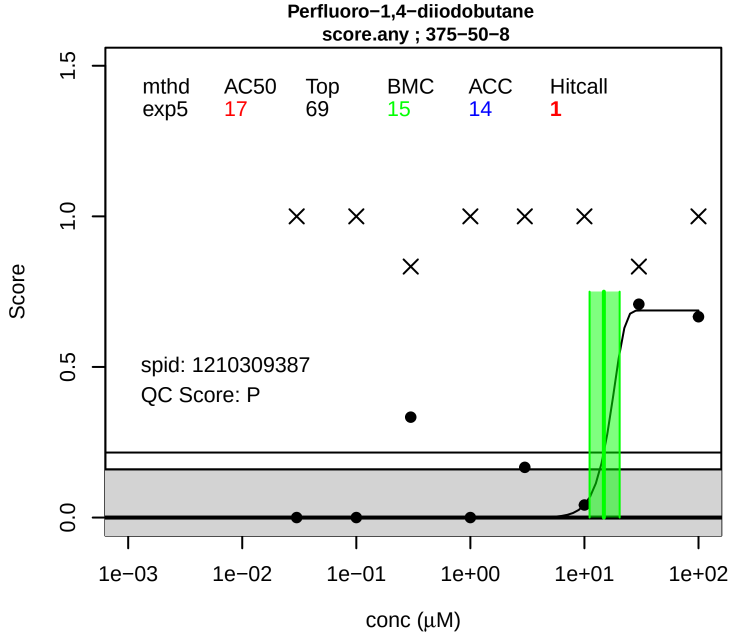
Perfluoro−1,4−diiodobutane
score.any ; 375−50−8
mthd AC50 Top BMC ACC Hitcall
exp5 17 69 15 14 1
spid: 1210309387
QC Score: P
Score
conc (µM)

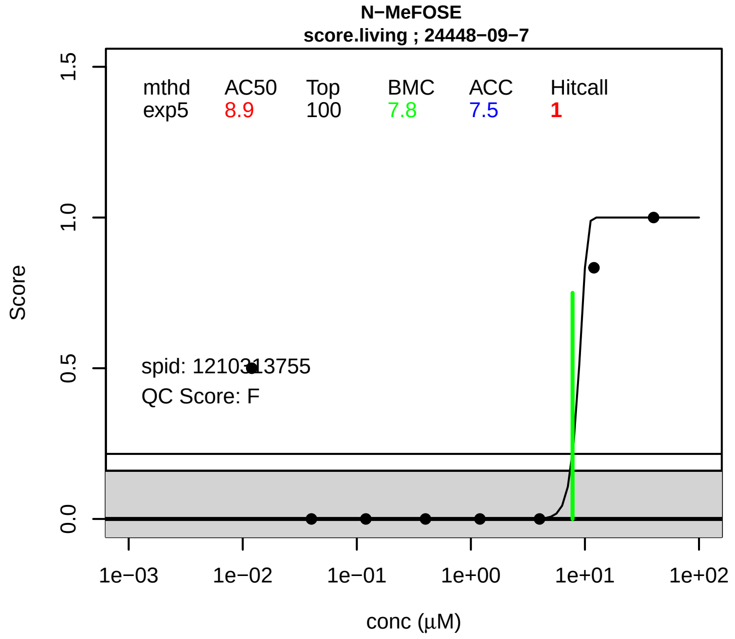
N−MeFOSE
score.living ; 24448−09−7
mthd AC50 Top BMC ACC Hitcall
exp5 8.9 100 7.8 7.5 1
spid: 1210313755
QC Score: F
Score
conc (µM)

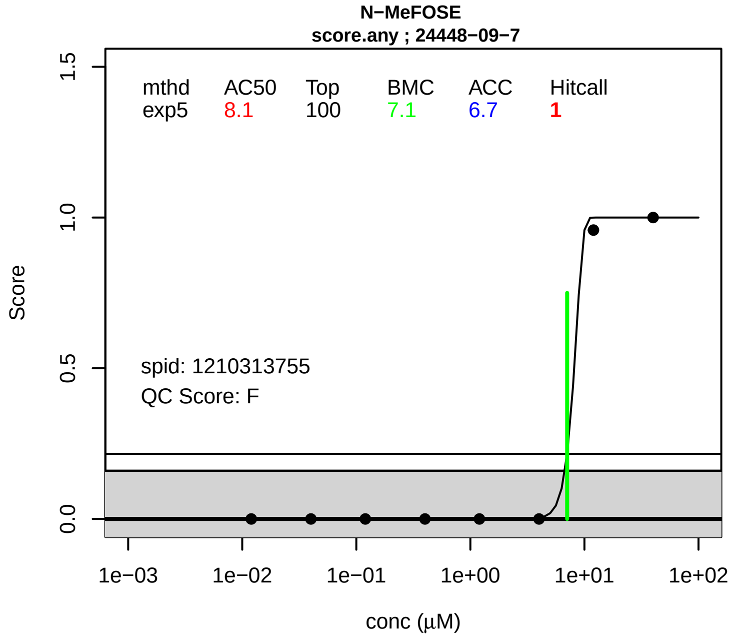
N−MeFOSE
score.any ; 24448−09−7
mthd AC50 Top BMC ACC Hitcall
exp5 8.1 100 7.1 6.7 1
spid: 1210313755
QC Score: F
Score
conc (µM)

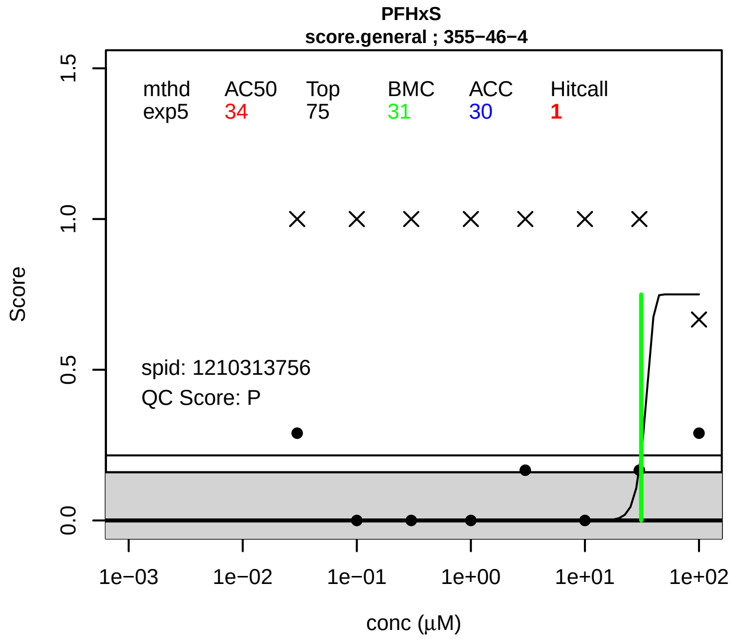
PFHxS
score.general ; 355−46−4
mthd exp5
AC50 34
Top 75
BMC 31
ACC 30
Hitcall 1
spid: 1210313756
QC Score: P
Score
conc (µM)

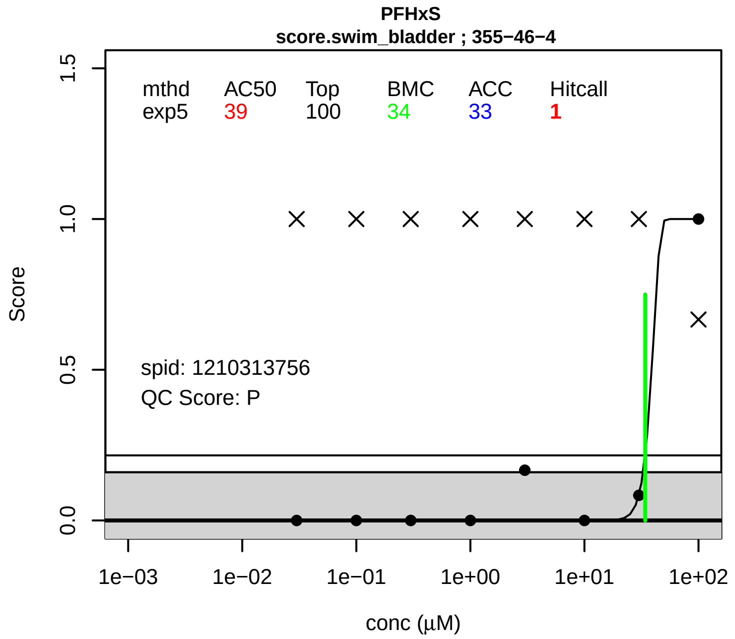
PFHxS
score.swim_bladder ; 355−46−4
mthd exp5
AC50 39
Top 100
BMC 34
ACC 33
Hitcall 1
spid: 1210313756
QC Score: P
Score
conc (µM)

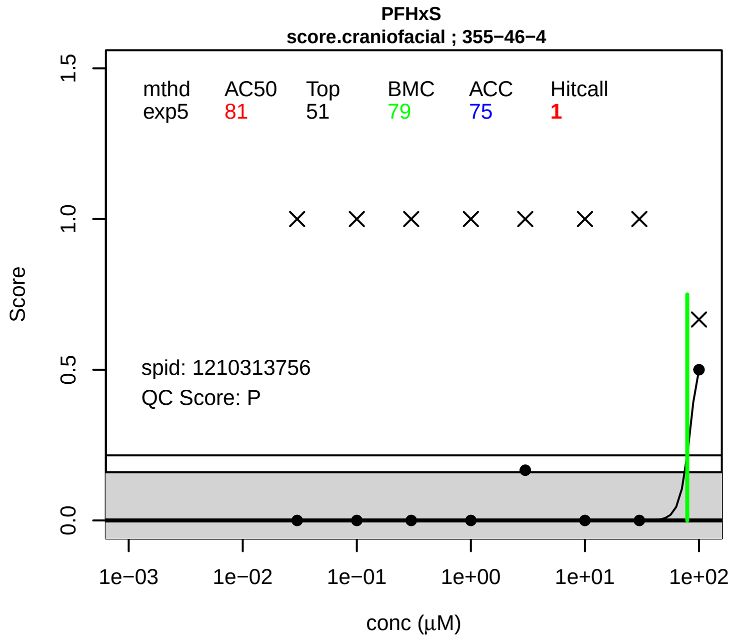
PFHxS
score.craniofacial ; 355−46−4
mthd exp5
AC50 81
Top 51
BMC 79
ACC 75
Hitcall 1
spid: 1210313756
QC Score: P
Score
conc (µM)

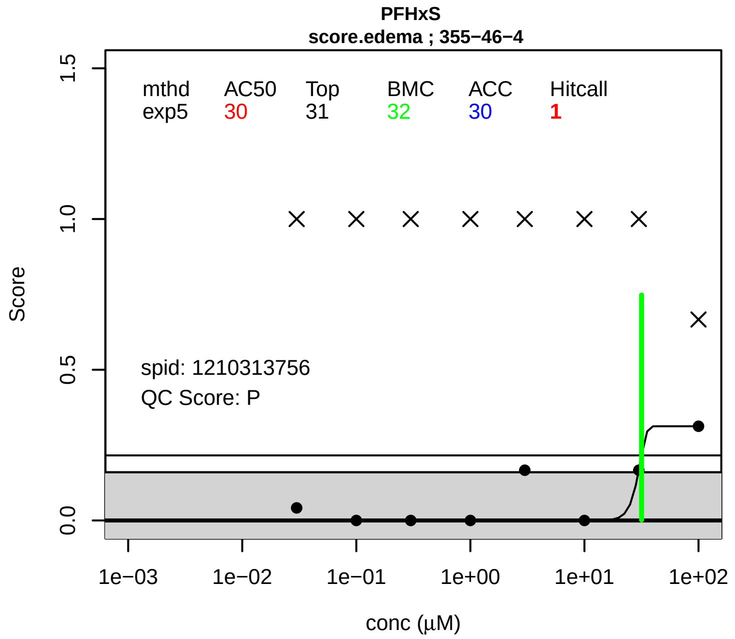
PFHxS
score.edema ; 355−46−4
mthd exp5
AC50 30
Top 31
BMC 32
ACC 30
Hitcall 1
spid: 1210313756
QC Score: P
Score
conc (µM)

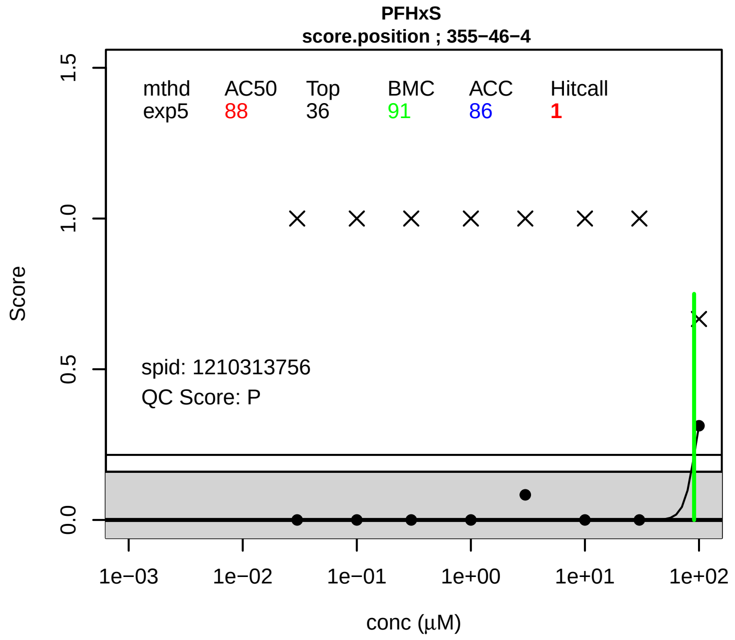
PFHxS
score.position ; 355−46−4
mthd exp5
AC50 88
Top 36
BMC 91
ACC 86
Hitcall 1
spid: 1210313756
QC Score: P
Score
conc (µM)

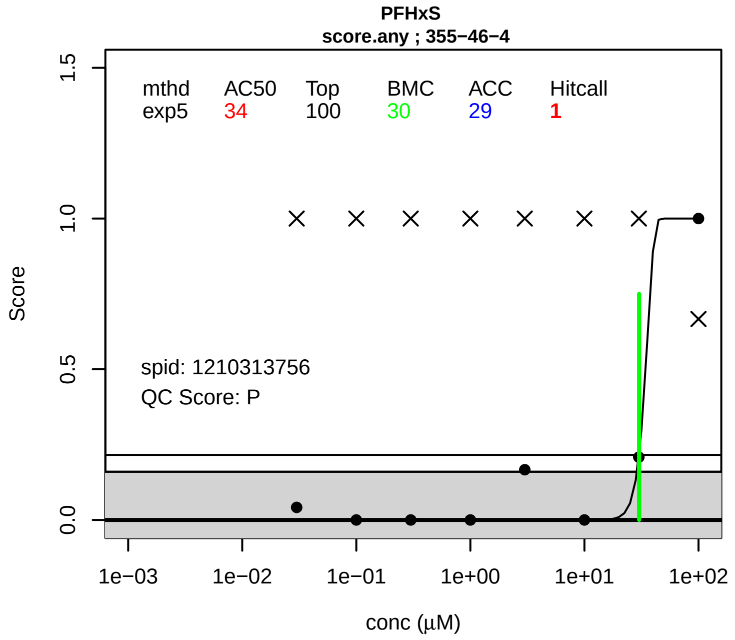
PFHxS
score.any ; 355−46−4
mthd exp5
AC50 34
Top 100
BMC 30
ACC 29
Hitcall 1
spid: 1210313756
QC Score: P
Score
conc (µM)

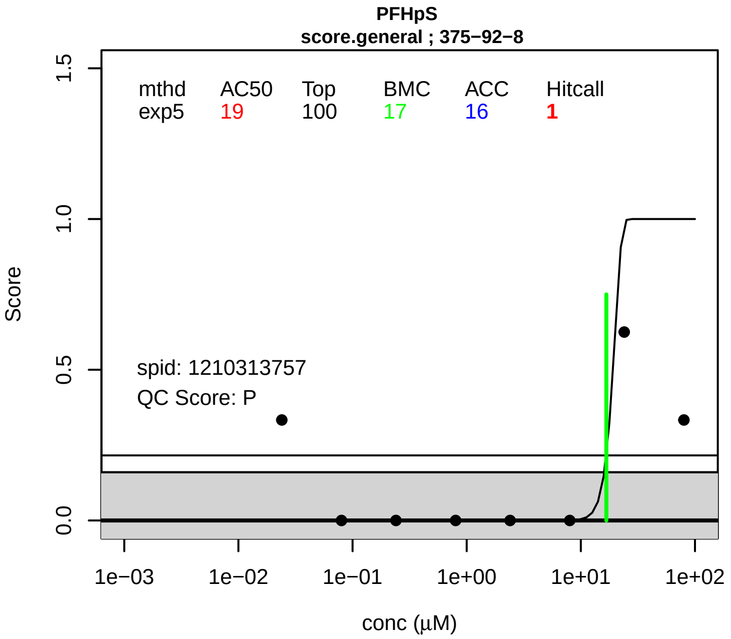
PFHpS
score.general ; 375−92−8
mthd exp5
AC50 19
Top 100
BMC 17
ACC 16
Hitcall 1
spid: 1210313757
QC Score: P
Score
conc (µM)
0.0 0.5 1.0 1.5
1e−03 1e−02 1e−01 1e+00 1e+01 1e+02

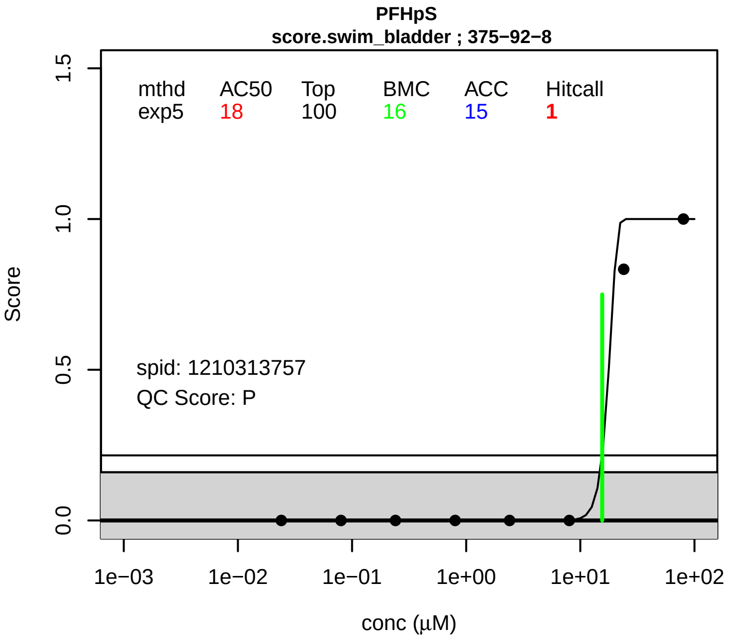
PFHpS
score.swim_bladder ; 375−92−8
mthd exp5
AC50 18
Top 100
BMC 16
ACC 15
Hitcall 1
spid: 1210313757
QC Score: P
Score
conc (µM)
0.0 0.5 1.0 1.5
1e−03 1e−02 1e−01 1e+00 1e+01 1e+02

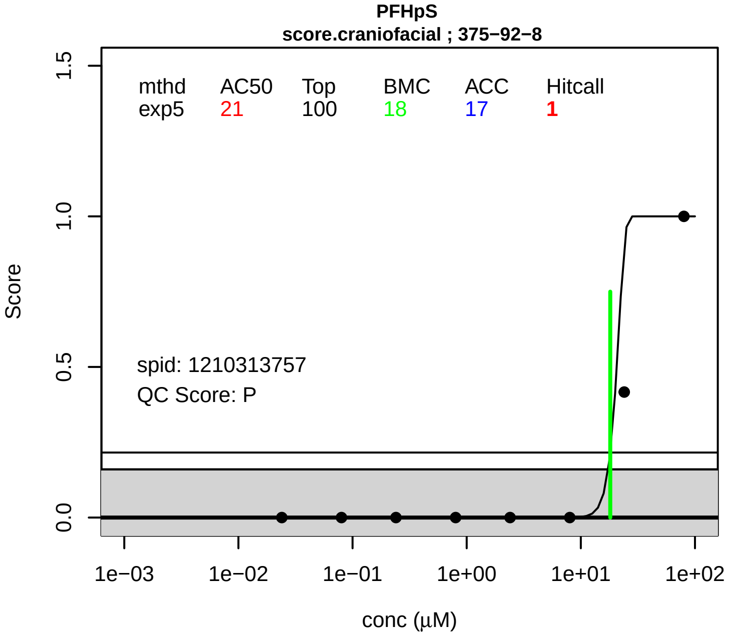
PFHpS
score.craniofacial ; 375−92−8
mthd exp5
AC50 21
Top 100
BMC 18
ACC 17
Hitcall 1
spid: 1210313757
QC Score: P
Score
conc (µM)
0.0 0.5 1.0 1.5
1e−03 1e−02 1e−01 1e+00 1e+01 1e+02

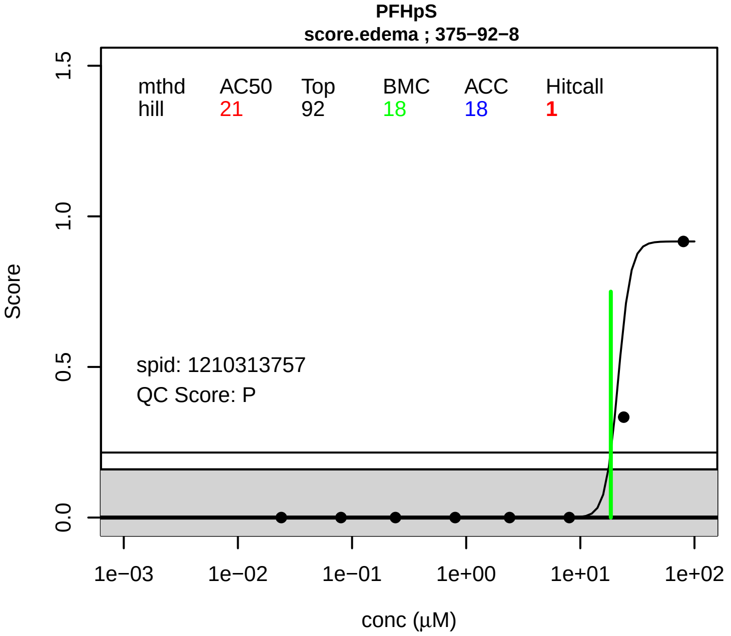
PFHpS
score.edema ; 375−92−8
mthd hill
AC50 21
Top 92
BMC 18
ACC 18
Hitcall 1
spid: 1210313757
QC Score: P
Score
conc (µM)
0.0 0.5 1.0 1.5
1e−03 1e−02 1e−01 1e+00 1e+01 1e+02

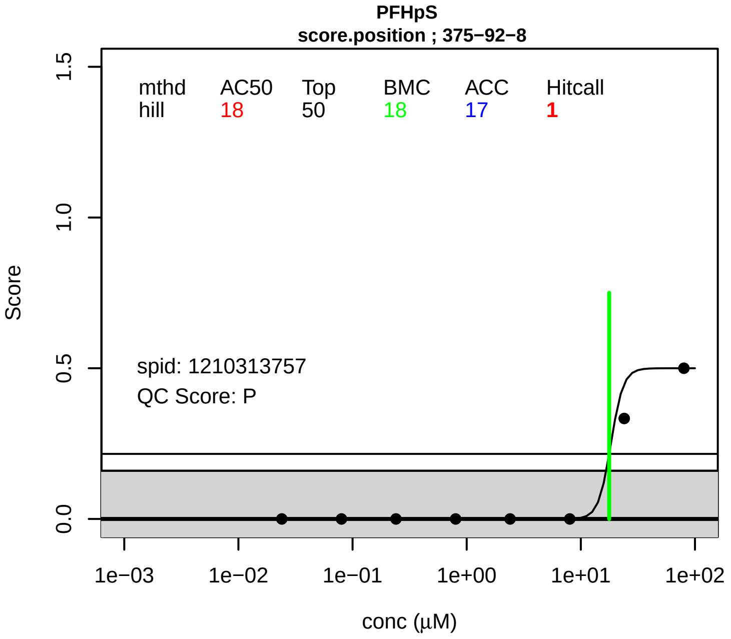
PFHpS
score.position ; 375−92−8
mthd hill
AC50 18
Top 50
BMC 18
ACC 17
Hitcall 1
spid: 1210313757
QC Score: P
Score
conc (µM)
0.0 0.5 1.0 1.5
1e−03 1e−02 1e−01 1e+00 1e+01 1e+02

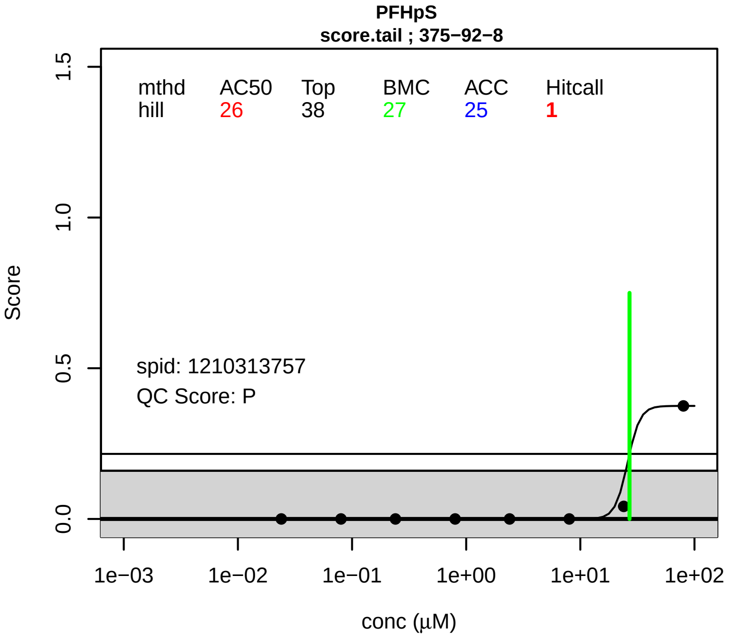
PFHpS
score.tail ; 375−92−8
mthd hill
AC50 26
Top 38
BMC 27
ACC 25
Hitcall 1
spid: 1210313757
QC Score: P
Score
conc (µM)
0.0 0.5 1.0 1.5
1e−03 1e−02 1e−01 1e+00 1e+01 1e+02

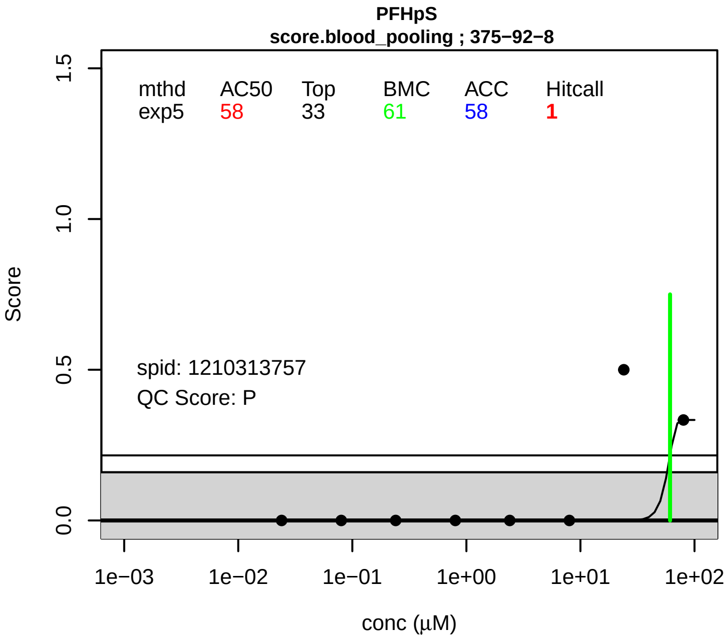
PFHpS
score.blood_pooling ; 375−92−8
mthd AC50 Top BMC ACC Hitcall
exp5 58 33 61 58 1
spid: 1210313757
QC Score: P
Score
1.5
1.0
0.5
0.0
1e−03 1e−02 1e−01 1e+00 1e+01 1e+02
conc (µM)

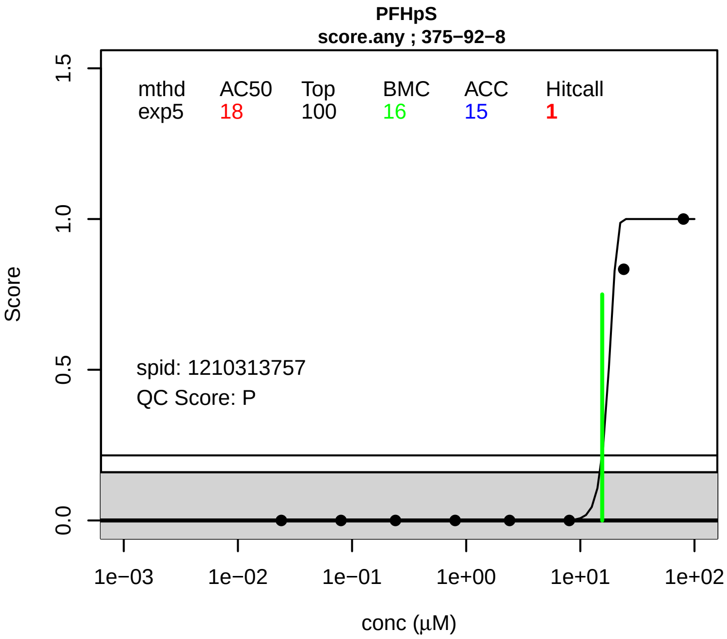
PFHpS
score.any ; 375−92−8
mthd AC50 Top BMC ACC Hitcall
exp5 18 100 16 15 1
spid: 1210313757
QC Score: P
Score
1.5
1.0
0.5
0.0
1e−03 1e−02 1e−01 1e+00 1e+01 1e+02
conc (µM)

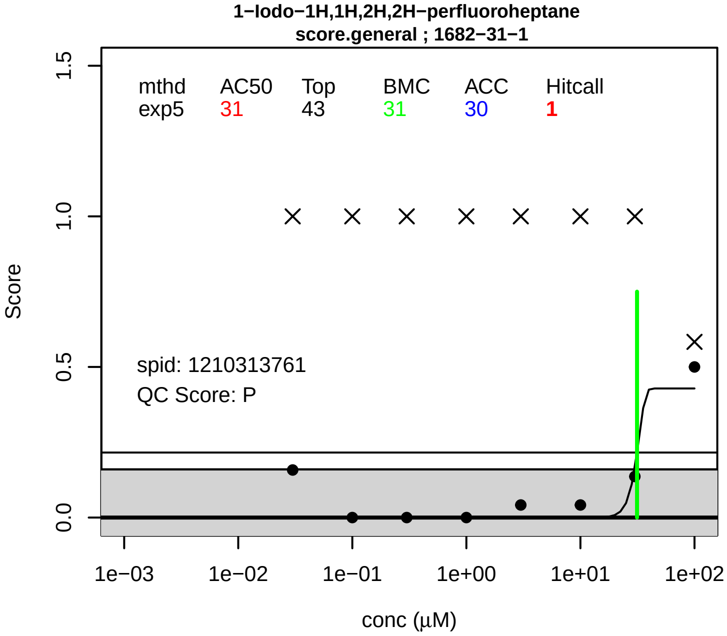
1−Iodo−1H,1H,2H,2H−perfluoroheptane
score.general ; 1682−31−1
mthd AC50 Top BMC ACC Hitcall
exp5 31 43 31 30 1
spid: 1210313761
QC Score: P
Score
1.5
1.0
0.5
0.0
1e−03 1e−02 1e−01 1e+00 1e+01 1e+02
conc (µM)

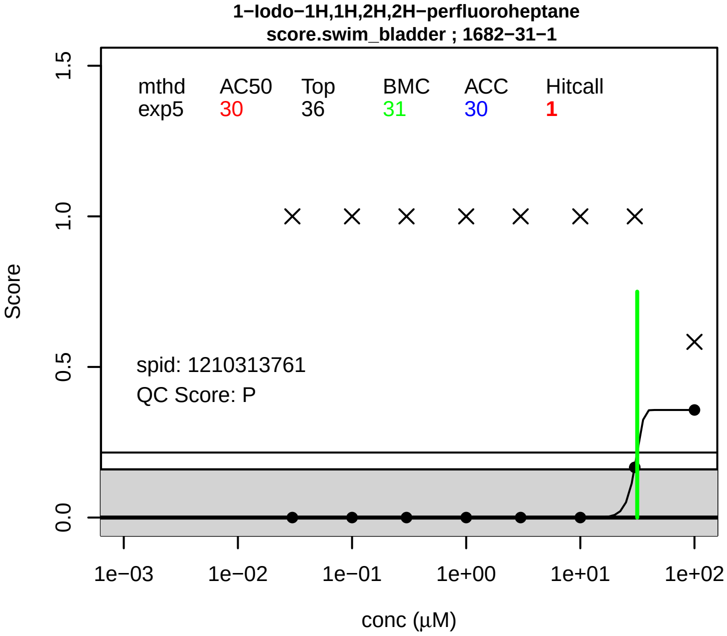
1−Iodo−1H,1H,2H,2H−perfluoroheptane
score.swim_bladder ; 1682−31−1
mthd AC50 Top BMC ACC Hitcall
exp5 30 36 31 30 1
spid: 1210313761
QC Score: P
Score
1.5
1.0
0.5
0.0
1e−03 1e−02 1e−01 1e+00 1e+01 1e+02
conc (µM)

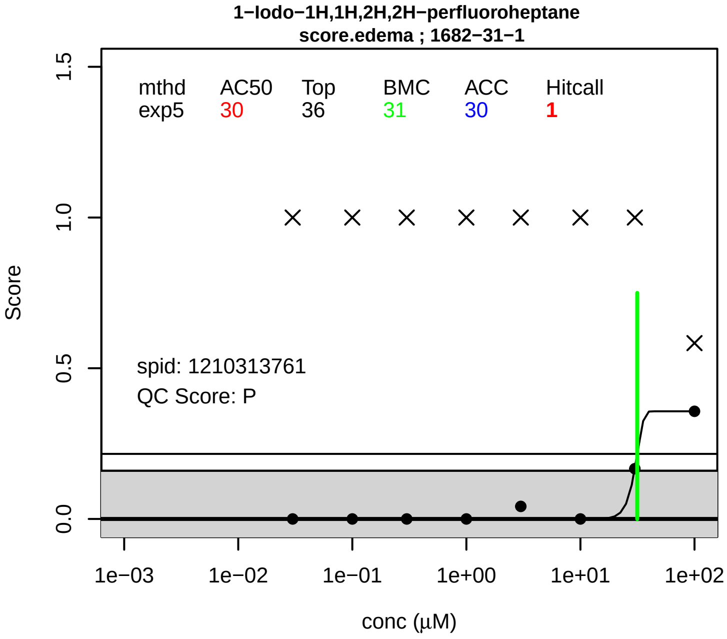
1−Iodo−1H,1H,2H,2H−perfluoroheptane
score.edema ; 1682−31−1
mthd AC50 Top BMC ACC Hitcall
exp5 30 36 31 30 1
spid: 1210313761
QC Score: P
Score
1.5
1.0
0.5
0.0
1e−03 1e−02 1e−01 1e+00 1e+01 1e+02
conc (µM)

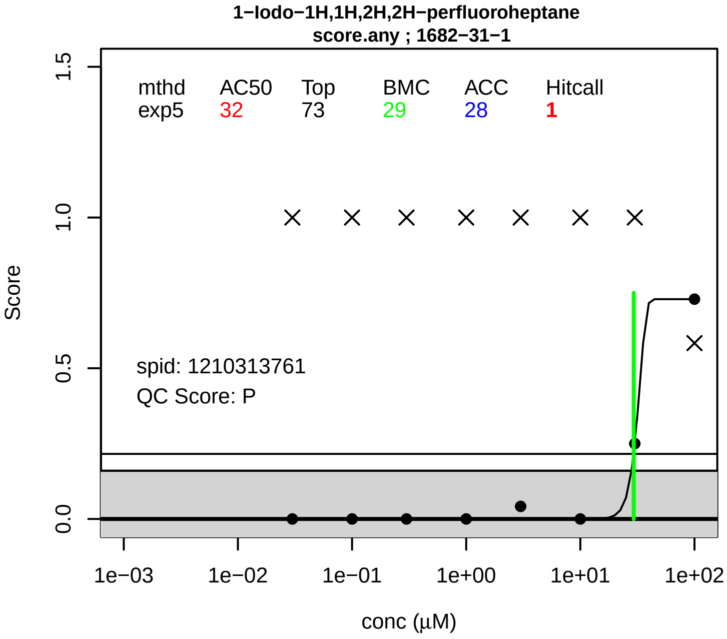
1−Iodo−1H,1H,2H,2H−perfluoroheptane
score.any ; 1682−31−1
mthd AC50 Top BMC ACC Hitcall
exp5 32 73 29 28 1
spid: 1210313761
QC Score: P
Score
1.5
1.0
0.5
0.0
1e−03 1e−02 1e−01 1e+00 1e+01 1e+02
conc (µM)

1H,1H,6H,6H−Perfluorohexane−1,6−diol diacrylate
score.living ; 2264−01−9

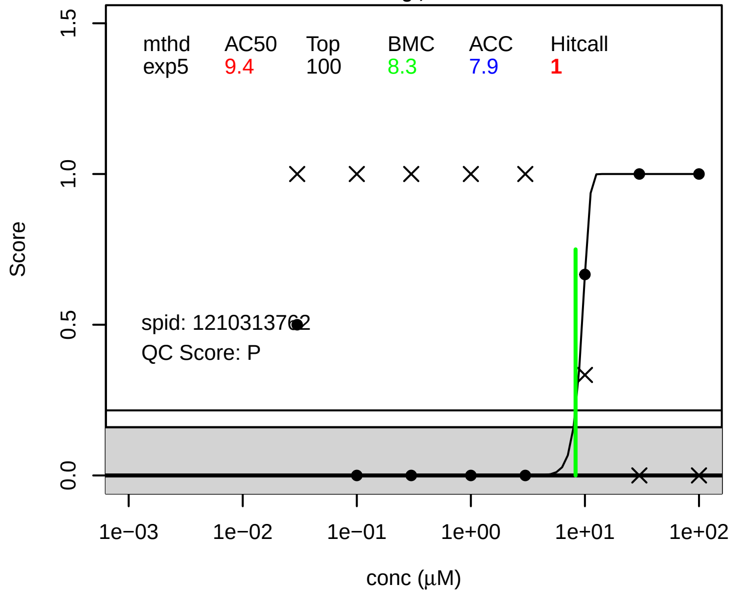

1H,1H,6H,6H−Perfluorohexane−1,6−diol diacrylate
score.general ; 2264−01−9

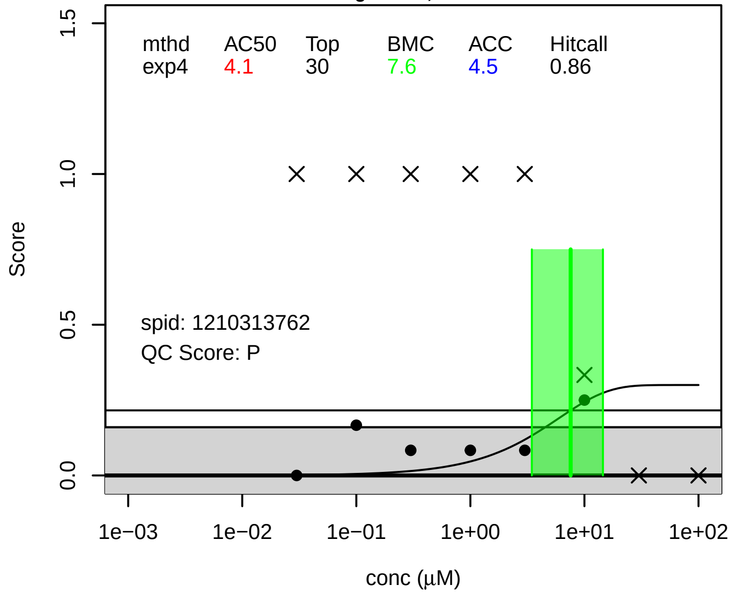

1H,1H,6H,6H−Perfluorohexane−1,6−diol diacrylate
score.swim_bladder ; 2264−01−9

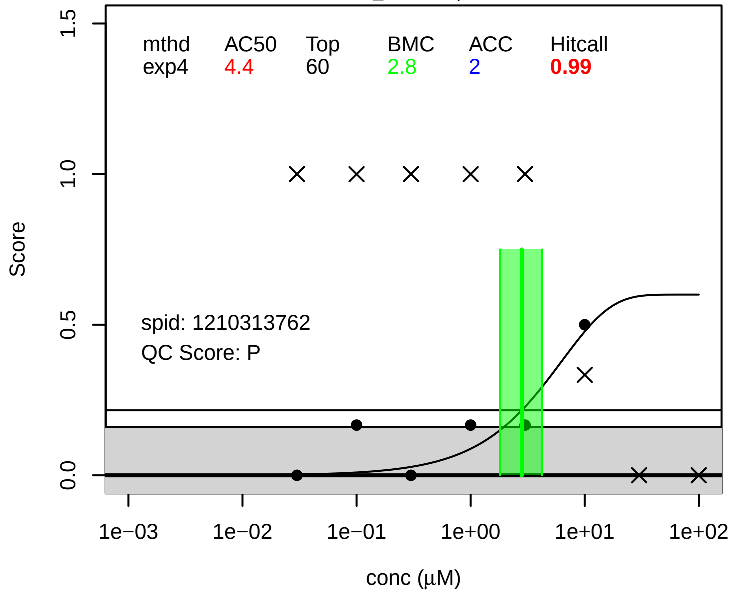

1H,1H,6H,6H−Perfluorohexane−1,6−diol diacrylate
score.edema ; 2264−01−9

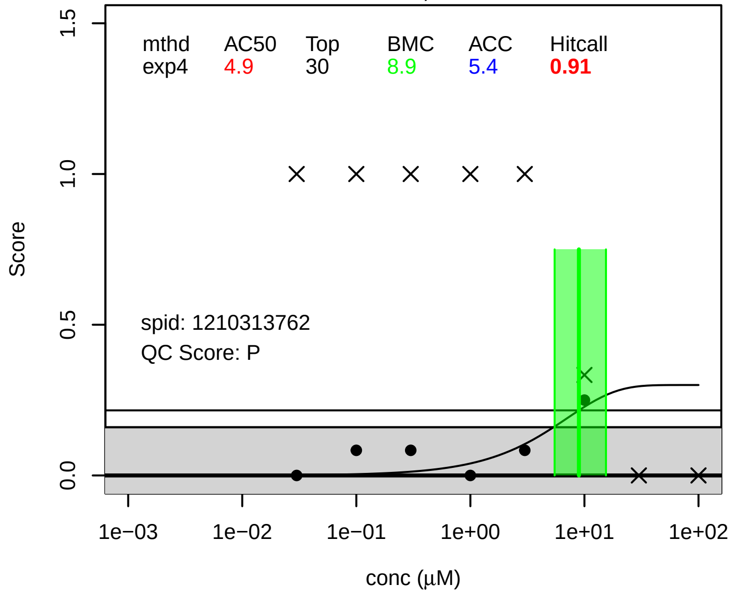

1H,1H,6H,6H−Perfluorohexane−1,6−diol diacrylate
score.any ; 2264−01−9

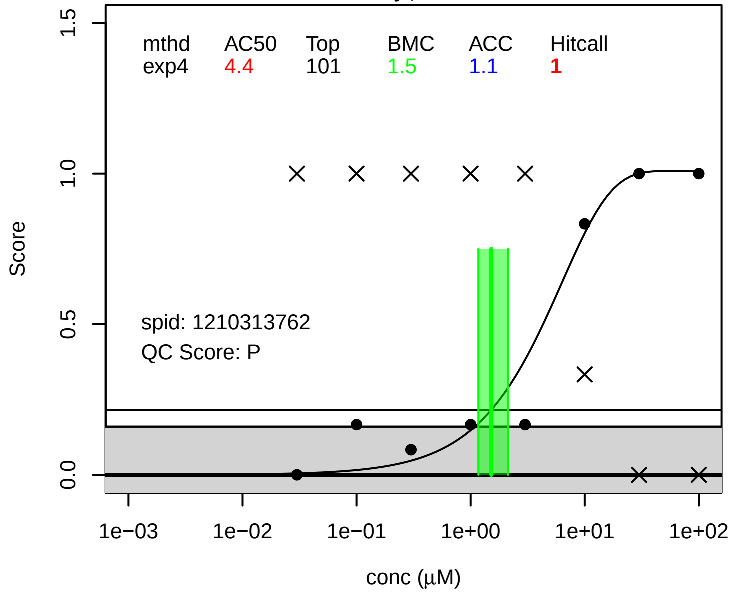

1−(Perfluorofluorooctyl)propane−2,3−diol
score.general ; 94159−84−9

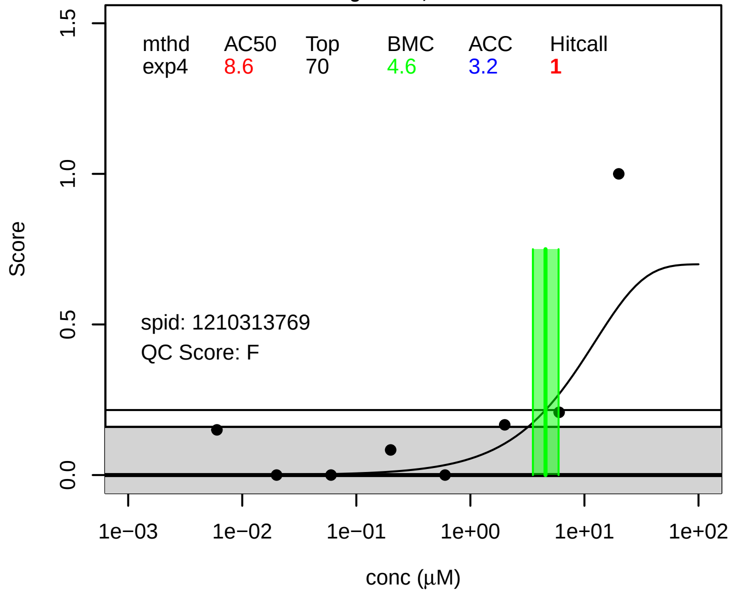

1−(Perfluorofluorooctyl)propane−2,3−diol
score.swim_bladder ; 94159−84−9

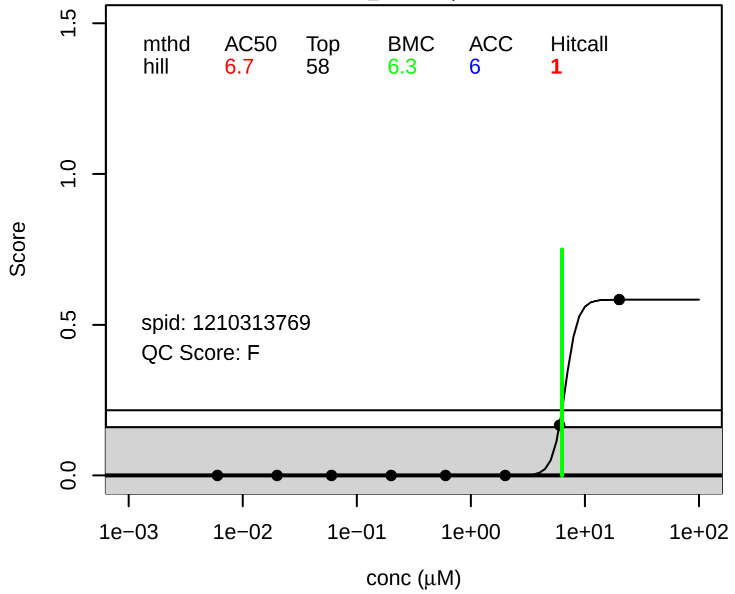

1−(Perfluorofluorooctyl)propane−2,3−diol
score.craniofacial ; 94159−84−9

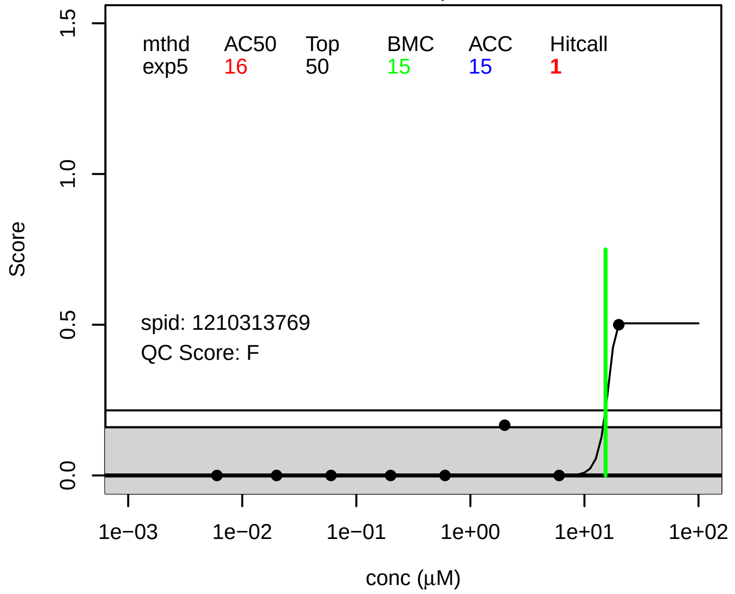

1−(Perfluorofluorooctyl)propane−2,3−diol
score.edema ; 94159−84−9

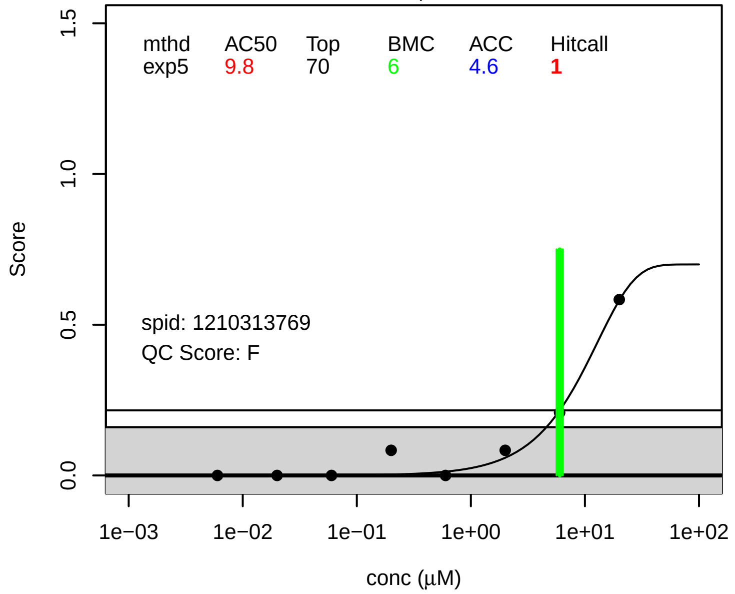

1−(Perfluorofluorooctyl)propane−2,3−diol
score.position ; 94159−84−9

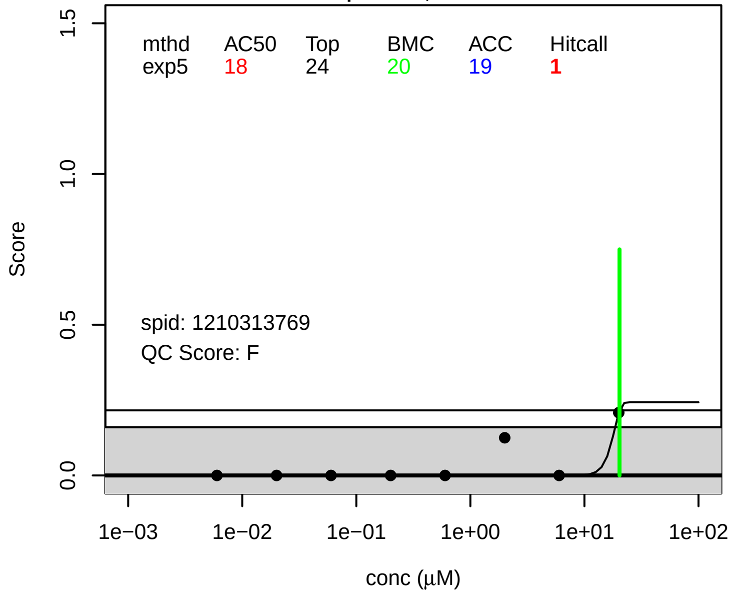

1−(Perfluorofluorooctyl)propane−2,3−diol
score.any ; 94159−84−9

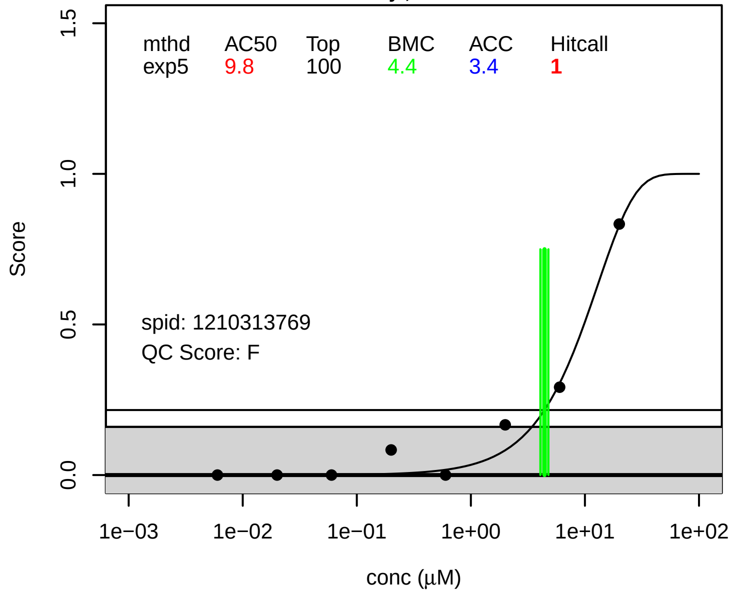

N−[(Perfluorooctylsulfonamido)propyl]−N,N,N−trimethylammonium iodide
score.living ; 1652−63−7

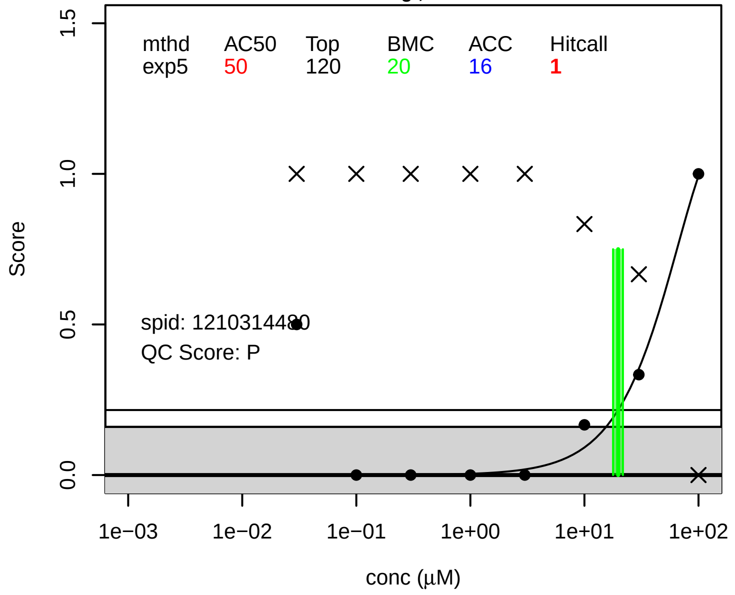

N−[(Perfluorooctylsulfonamido)propyl]−N,N,N−trimethylammonium iodide
score.any ; 1652−63−7

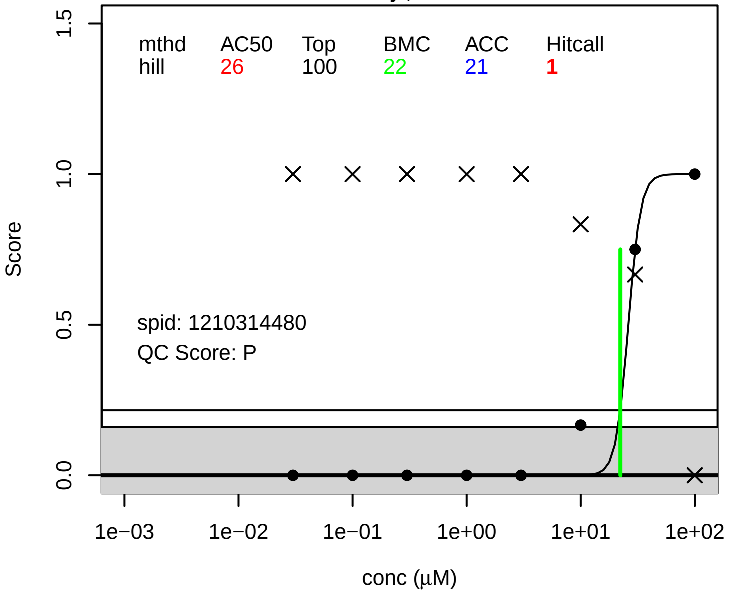

1−Iodopentadecafluoroheptane
score.general ; 335−58−0

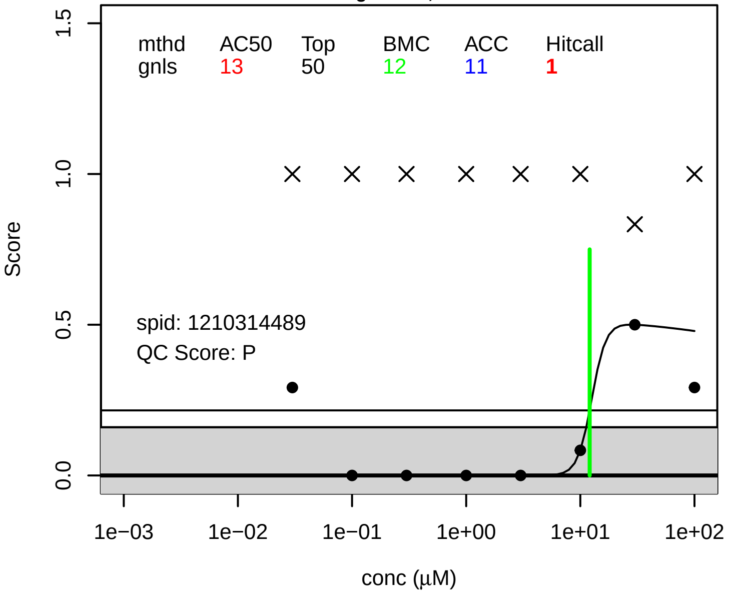

1−Iodopentadecafluoroheptane
score.swim_bladder ; 335−58−0

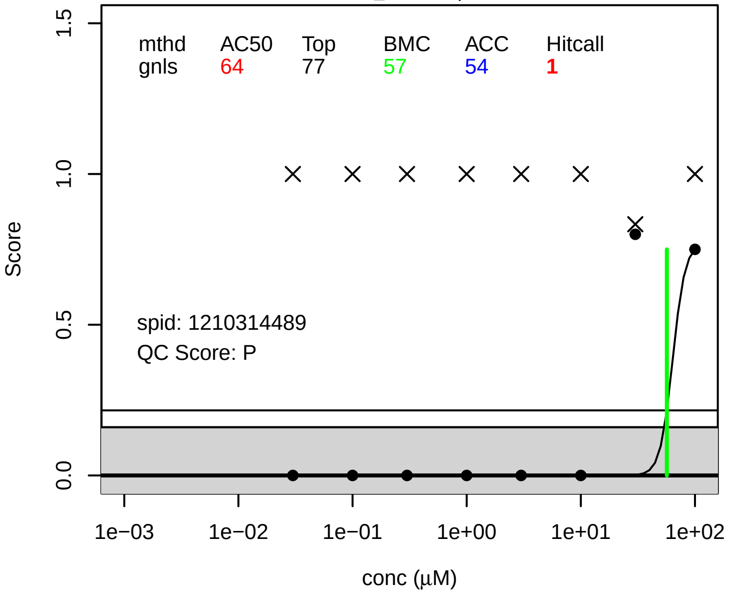

1−Iodopentadecafluoroheptane
score.any ; 335−58−0

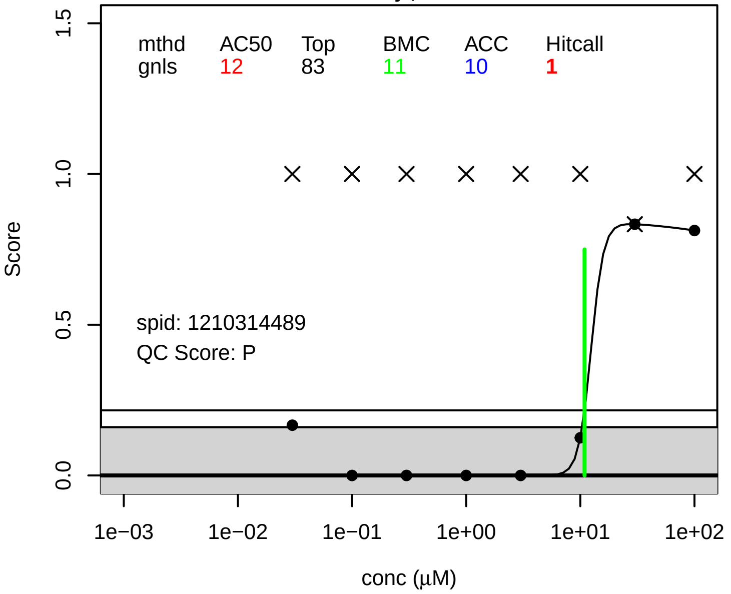

6:1 FTOH
score.living ; 375−82−6

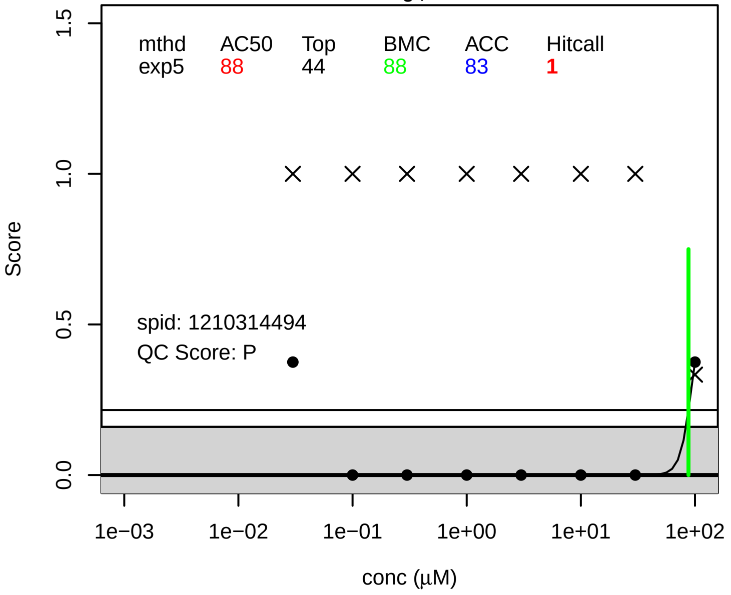

6:1 FTOH
score.general ; 375−82−6

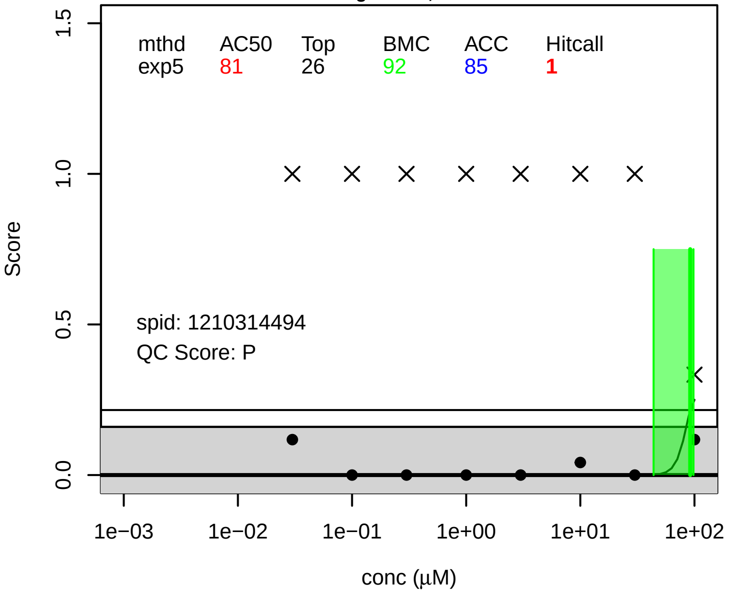

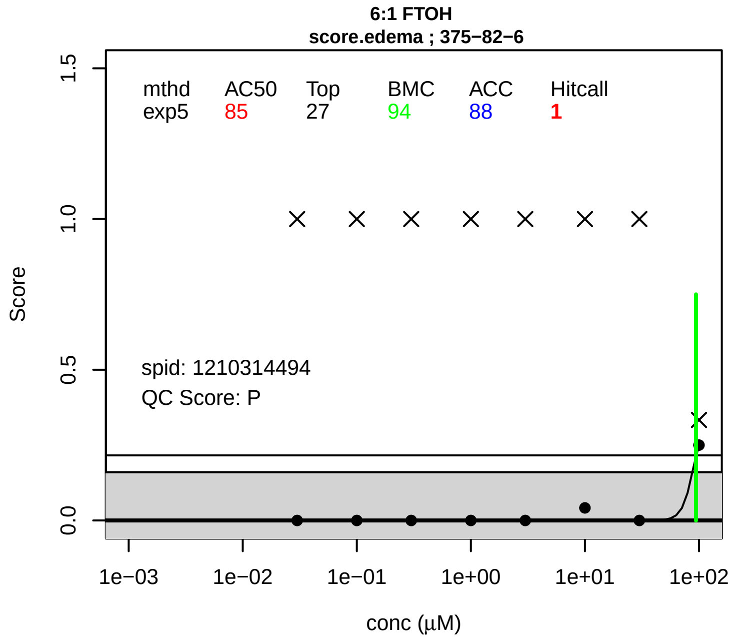
6:1 FTOH
score.edema ; 375−82−6
mthd exp5
AC50 85
Top 27
BMC 94
ACC 88
Hitcall 1
spid: 1210314494
QC Score: P
Score
conc (µM)

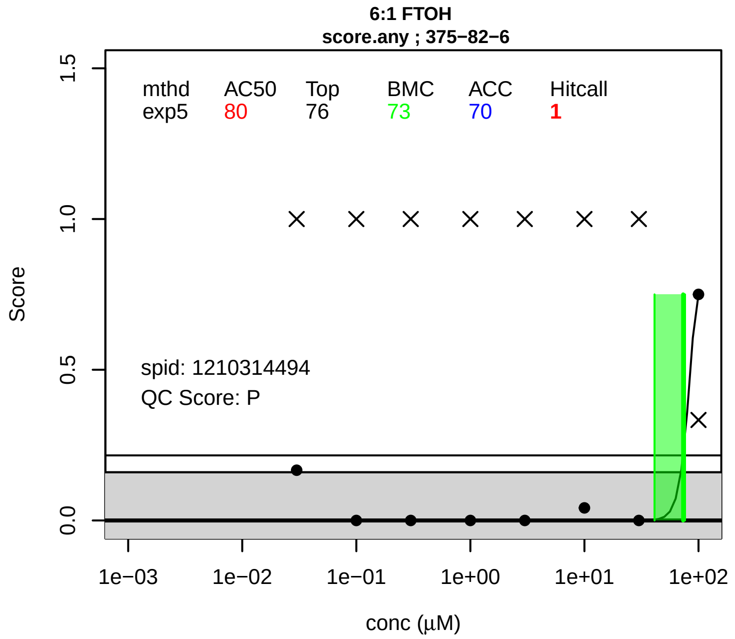
6:1 FTOH
score.any ; 375−82−6
mthd exp5
AC50 80
Top 76
BMC 73
ACC 70
Hitcall 1
spid: 1210314494
QC Score: P
Score
conc (µM)

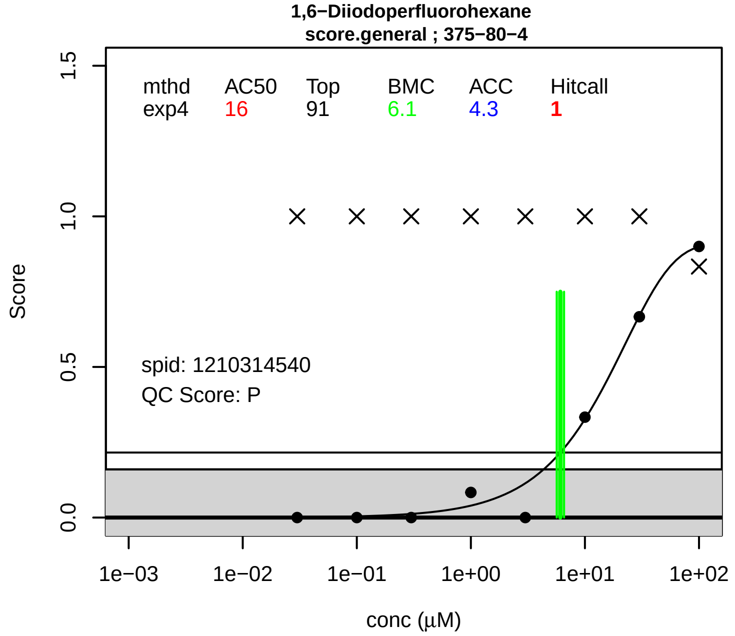
1,6−Diiodoperfluorohexane
score.general ; 375−80−4
mthd exp4
AC50 16
Top 91
BMC 6.1
ACC 4.3
Hitcall 1
spid: 1210314540
QC Score: P
Score
conc (µM)

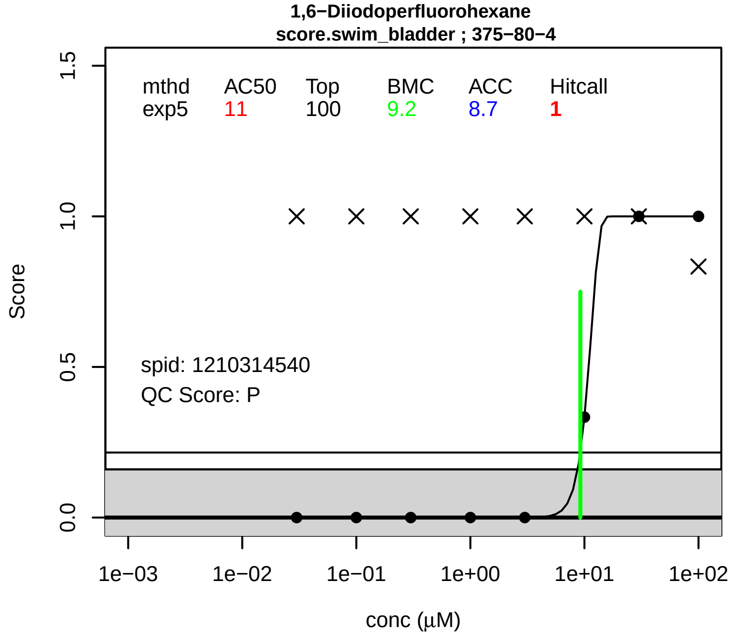
1,6−Diiodoperfluorohexane
score.swim_bladder ; 375−80−4
mthd exp5
AC50 11
Top 100
BMC 9.2
ACC 8.7
Hitcall 1
spid: 1210314540
QC Score: P
Score
conc (µM)

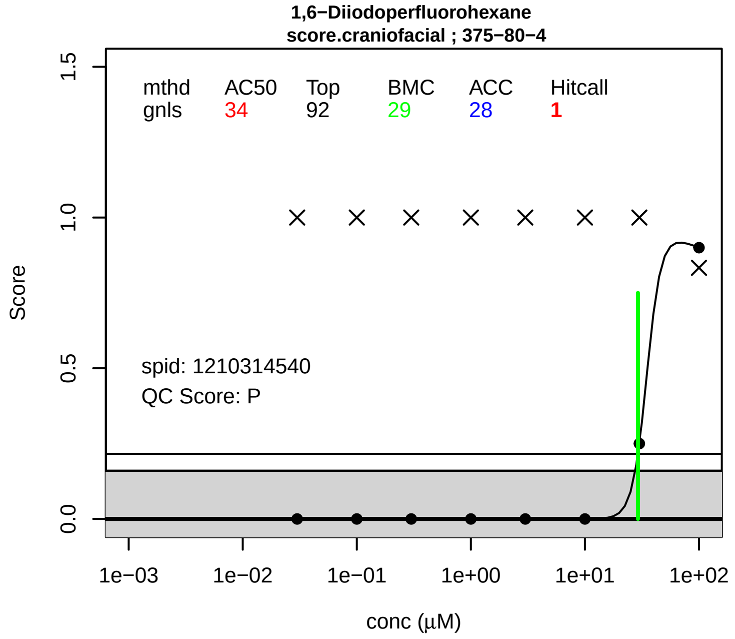
1,6−Diiodoperfluorohexane
score.craniofacial ; 375−80−4
mthd gnls
AC50 34
Top 92
BMC 29
ACC 28
Hitcall 1
spid: 1210314540
QC Score: P
Score
conc (µM)

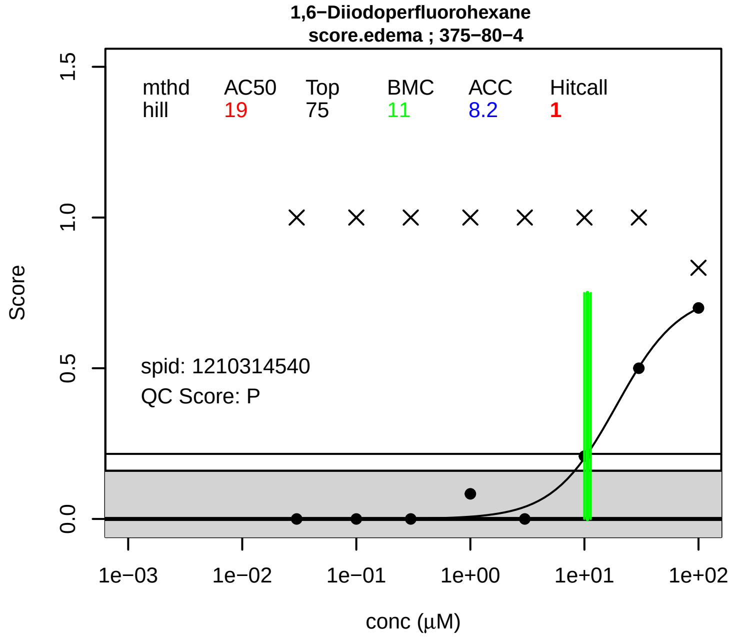
1,6−Diiodoperfluorohexane
score.edema ; 375−80−4
mthd hill
AC50 19
Top 75
BMC 11
ACC 8.2
Hitcall 1
spid: 1210314540
QC Score: P
Score
conc (µM)

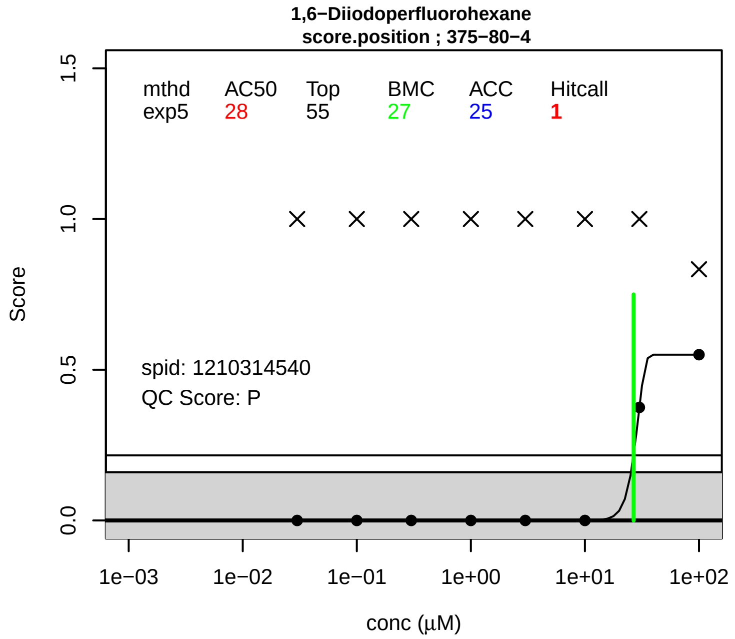
1,6−Diiodoperfluorohexane
score.position ; 375−80−4
mthd exp5
AC50 28
Top 55
BMC 27
ACC 25
Hitcall 1
spid: 1210314540
QC Score: P
Score
conc (µM)

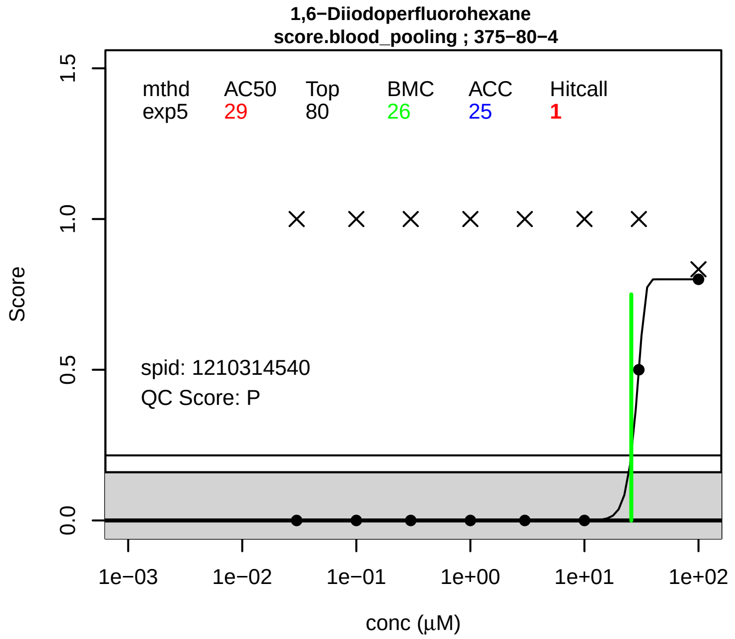
1,6−Diiodoperfluorohexane
score.blood_pooling ; 375−80−4
mthd exp5
AC50 29
Top 80
BMC 26
ACC 25
Hitcall 1
spid: 1210314540
QC Score: P
Score
conc (µM)

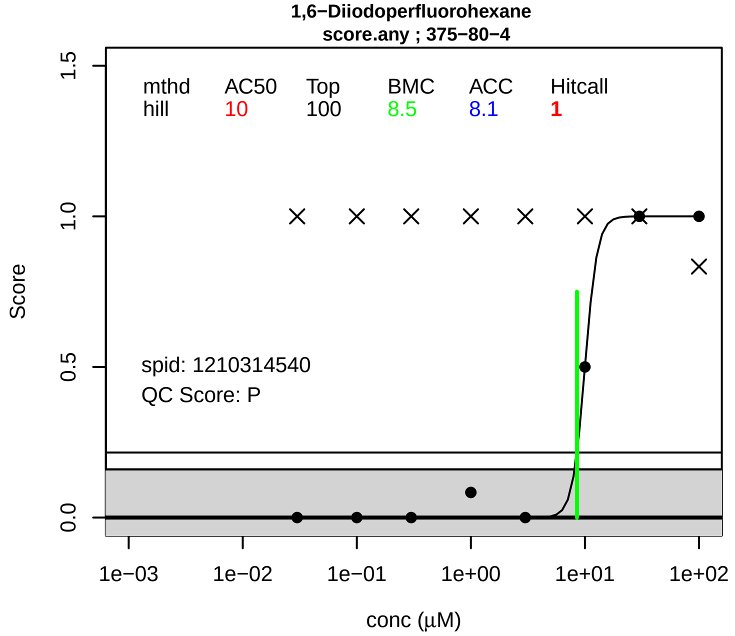
1,6−Diiodoperfluorohexane
score.any ; 375−80−4
mthd hill
AC50 10
Top 100
BMC 8.5
ACC 8.1
Hitcall 1
spid: 1210314540
QC Score: P
Score
conc (µM)

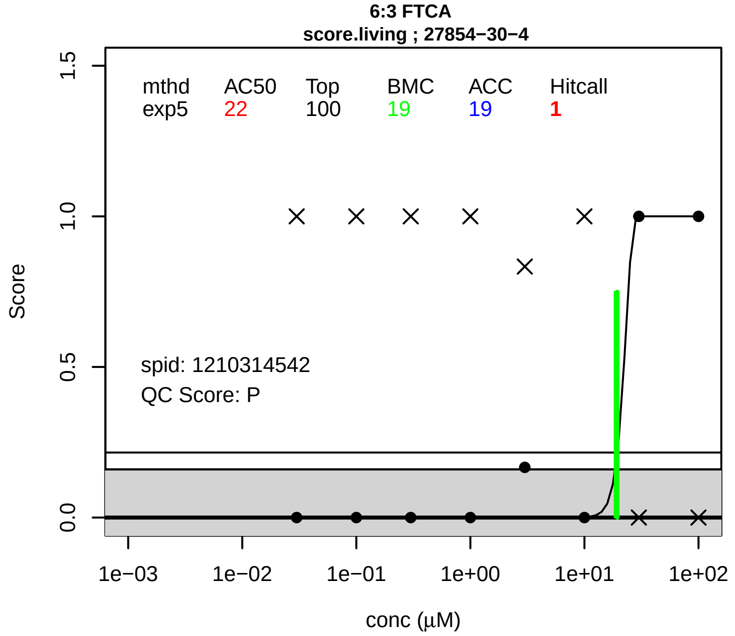
6:3 FTCA
score.living ; 27854−30−4
mthd exp5
AC50 22
Top 100
BMC 19
ACC 19
Hitcall 1
spid: 1210314542
QC Score: P
Score
conc (µM)

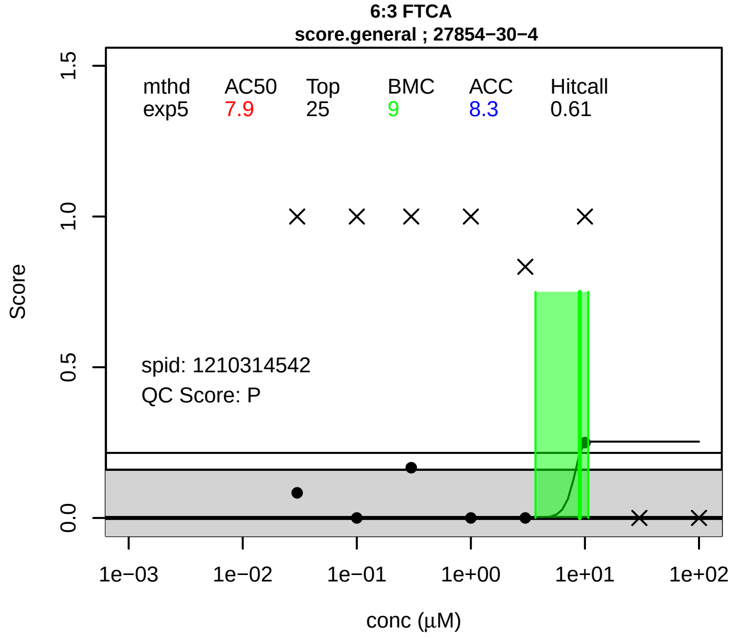
6:3 FTCA
score.general ; 27854−30−4
mthd exp5
AC50 7.9
Top 25
BMC 9
ACC 8.3
Hitcall 0.61
spid: 1210314542
QC Score: P
Score
conc (µM)

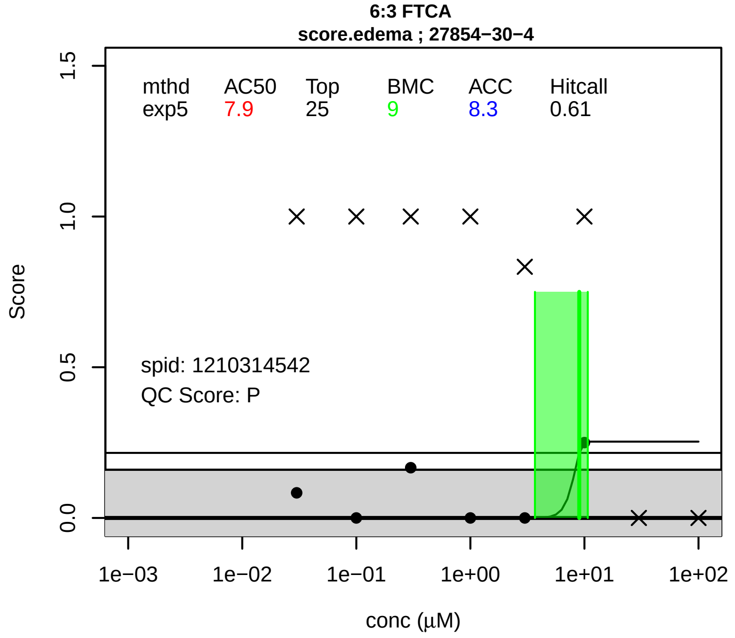
6:3 FTCA
score.edema ; 27854−30−4
mthd exp5
AC50 7.9
Top 25
BMC 9
ACC 8.3
Hitcall 0.61
spid: 1210314542
QC Score: P
Score
conc (µM)

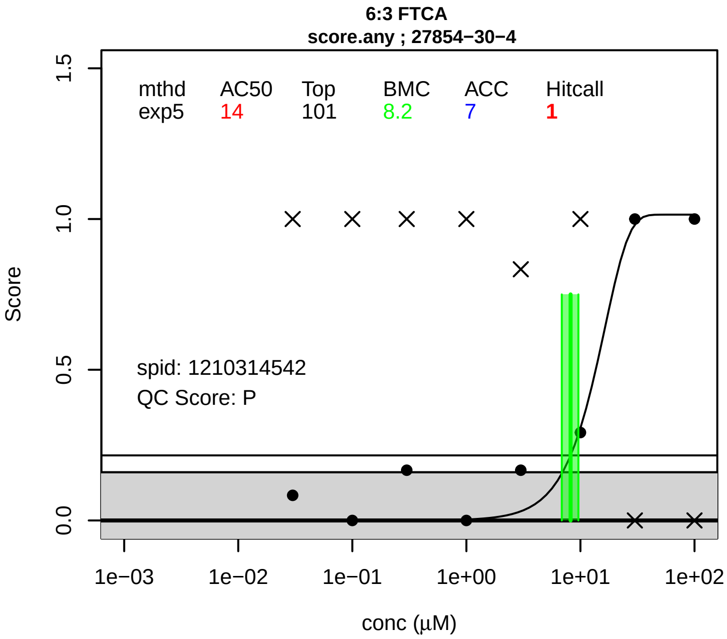
6:3 FTCA
score.any ; 27854−30−4
mthd AC50 Top BMC ACC Hitcall
exp5 14 101 8.2 7 1
spid: 1210314542
QC Score: P
Score
0.0 0.5 1.0 1.5
1e−03 1e−02 1e−01 1e+00 1e+01 1e+02
conc (µM)

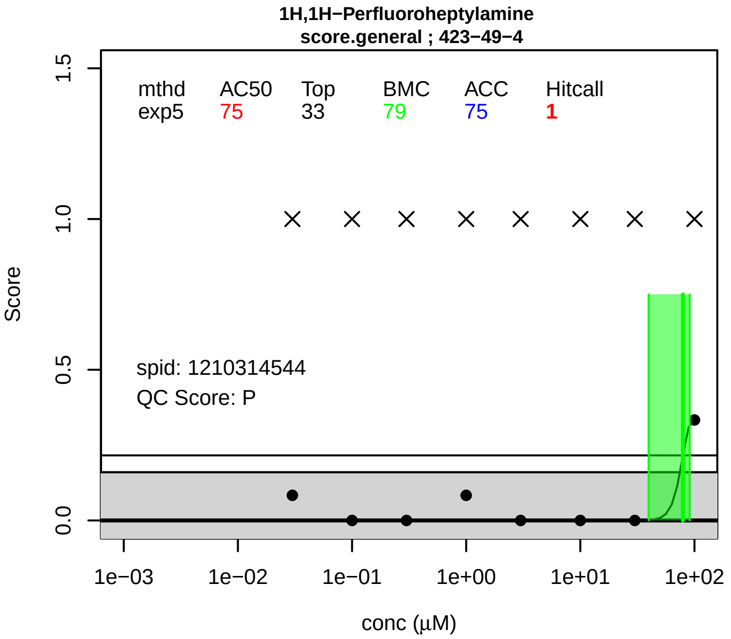
1H,1H−Perfluoroheptylamine
score.general ; 423−49−4
mthd AC50 Top BMC ACC Hitcall
exp5 75 33 79 75 1
spid: 1210314544
QC Score: P
Score
0.0 0.5 1.0 1.5
1e−03 1e−02 1e−01 1e+00 1e+01 1e+02
conc (µM)

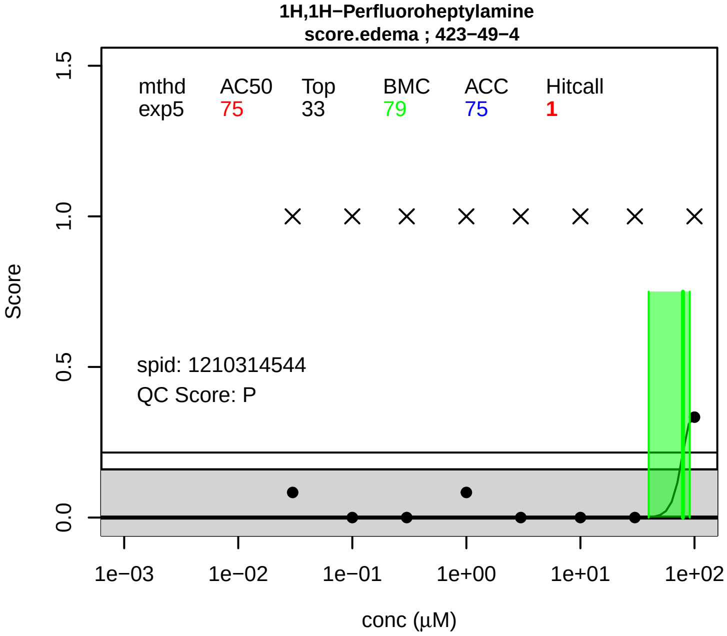
1H,1H−Perfluoroheptylamine
score.edema ; 423−49−4
mthd AC50 Top BMC ACC Hitcall
exp5 75 33 79 75 1
spid: 1210314544
QC Score: P
Score
0.0 0.5 1.0 1.5
1e−03 1e−02 1e−01 1e+00 1e+01 1e+02
conc (µM)

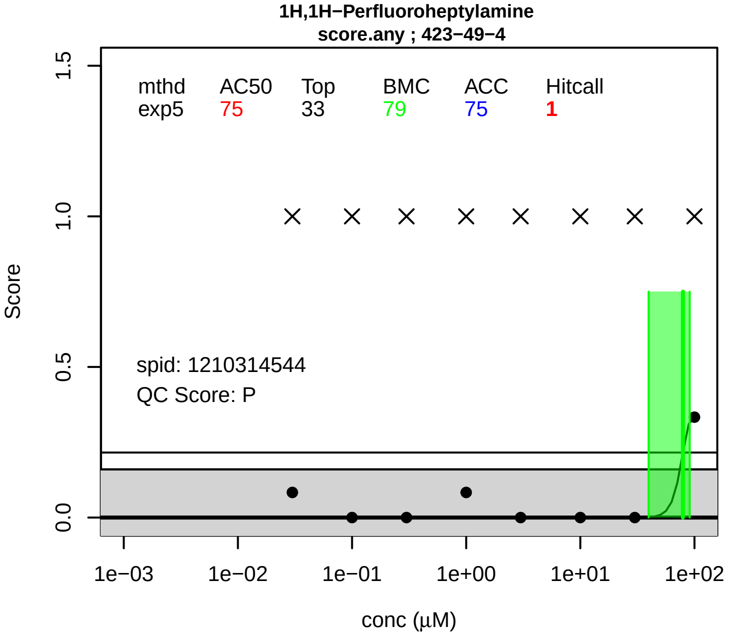
1H,1H−Perfluoroheptylamine
score.any ; 423−49−4
mthd AC50 Top BMC ACC Hitcall
exp5 75 33 79 75 1
spid: 1210314544
QC Score: P
Score
0.0 0.5 1.0 1.5
1e−03 1e−02 1e−01 1e+00 1e+01 1e+02
conc (µM)

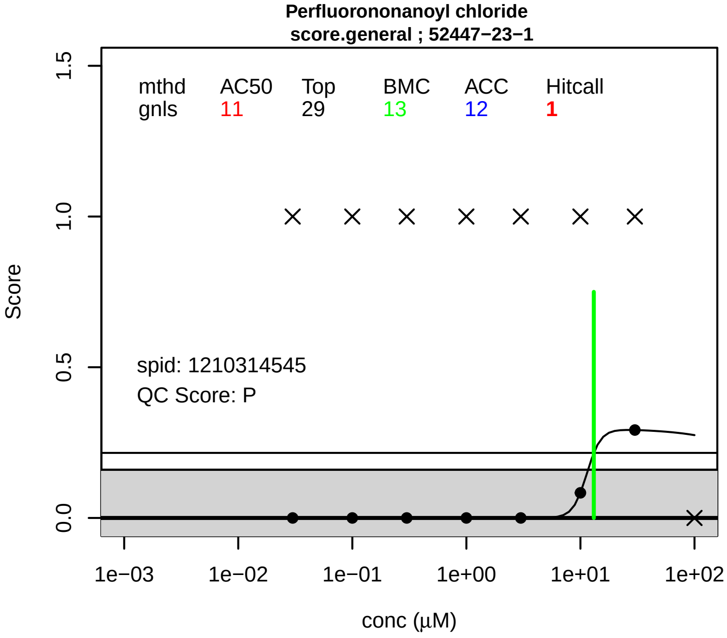
Perfluorononanoyl chloride
score.general ; 52447−23−1
mthd AC50 Top BMC ACC Hitcall
gnls 11 29 13 12 1
spid: 1210314545
QC Score: P
Score
0.0 0.5 1.0 1.5
1e−03 1e−02 1e−01 1e+00 1e+01 1e+02
conc (µM)

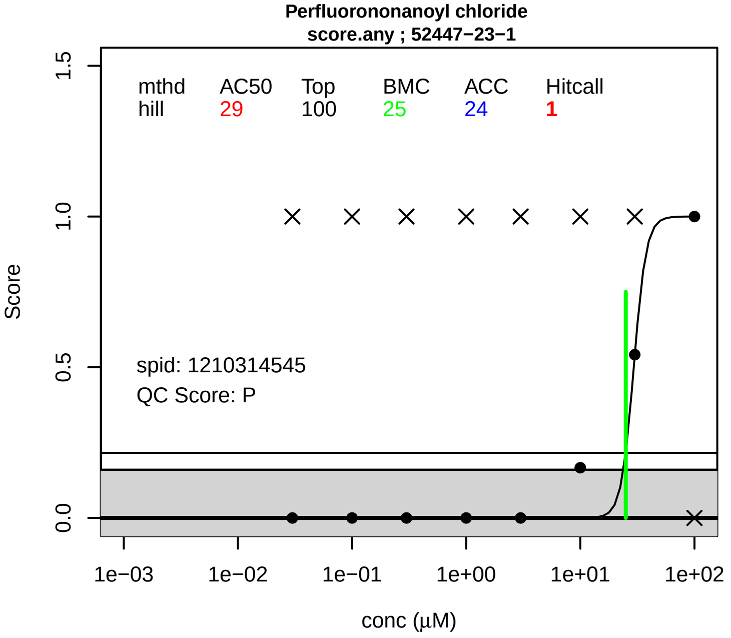
Perfluorononanoyl chloride
score.any ; 52447−23−1
mthd AC50 Top BMC ACC Hitcall
hill 29 100 25 24 1
spid: 1210314545
QC Score: P
Score
0.0 0.5 1.0 1.5
1e−03 1e−02 1e−01 1e+00 1e+01 1e+02
conc (µM)

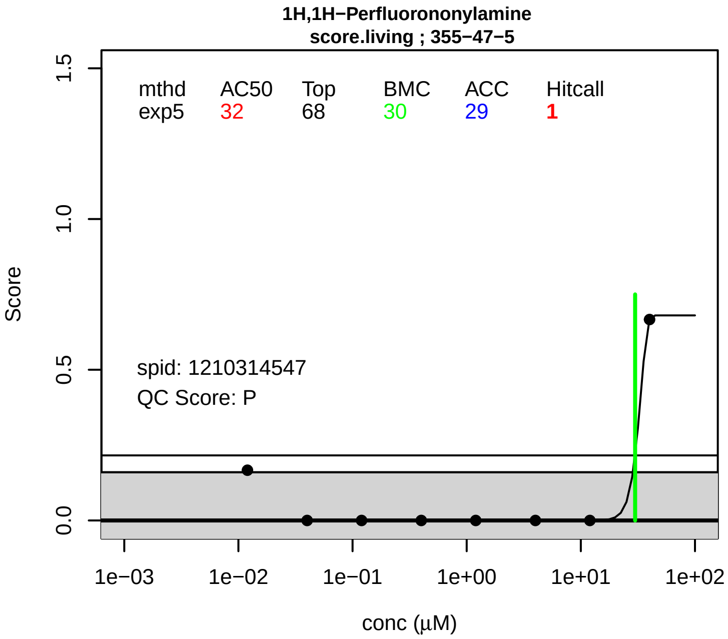
1H,1H−Perfluorononylamine
score.living ; 355−47−5
mthd AC50 Top BMC ACC Hitcall
exp5 32 68 30 29 1
spid: 1210314547
QC Score: P
Score
conc (µM)

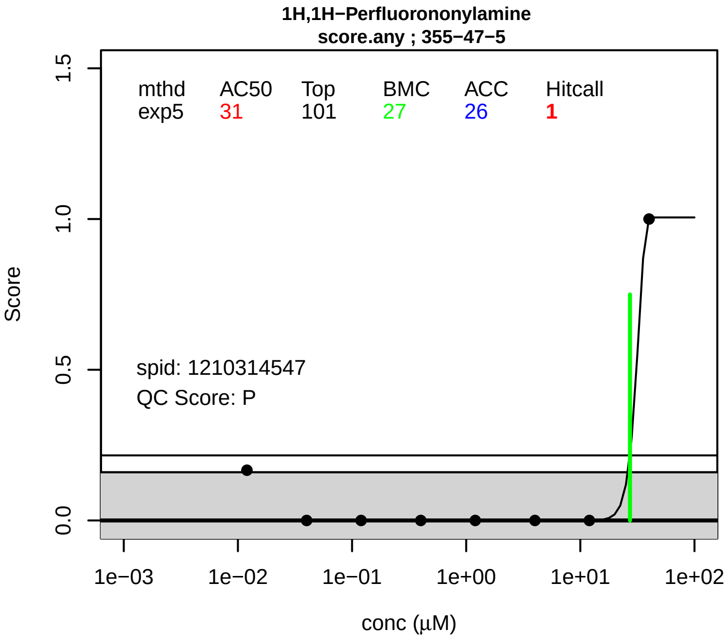
1H,1H−Perfluorononylamine
score.any ; 355−47−5
mthd AC50 Top BMC ACC Hitcall
exp5 31 101 27 26 1
spid: 1210314547
QC Score: P
Score
conc (µM)

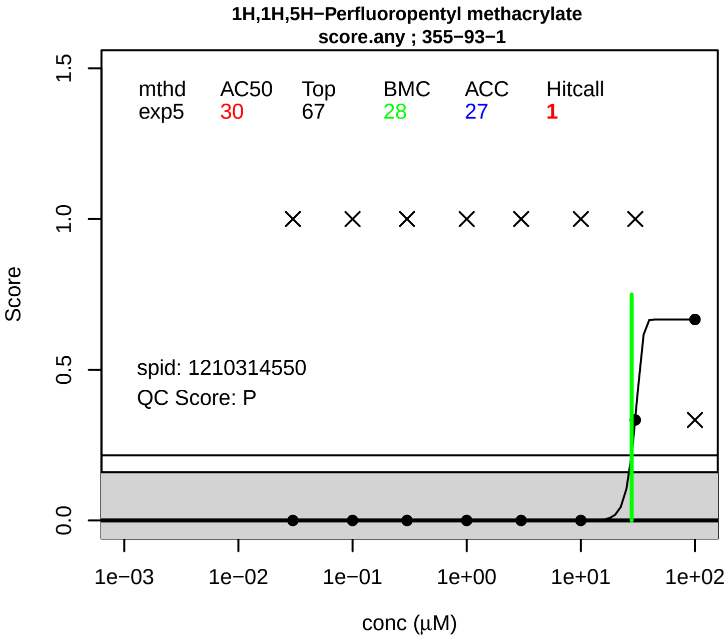
1H,1H,5H−Perfluoropentyl methacrylate
score.any ; 355−93−1
mthd AC50 Top BMC ACC Hitcall
exp5 30 67 28 27 1
spid: 1210314550
QC Score: P
Score
conc (µM)

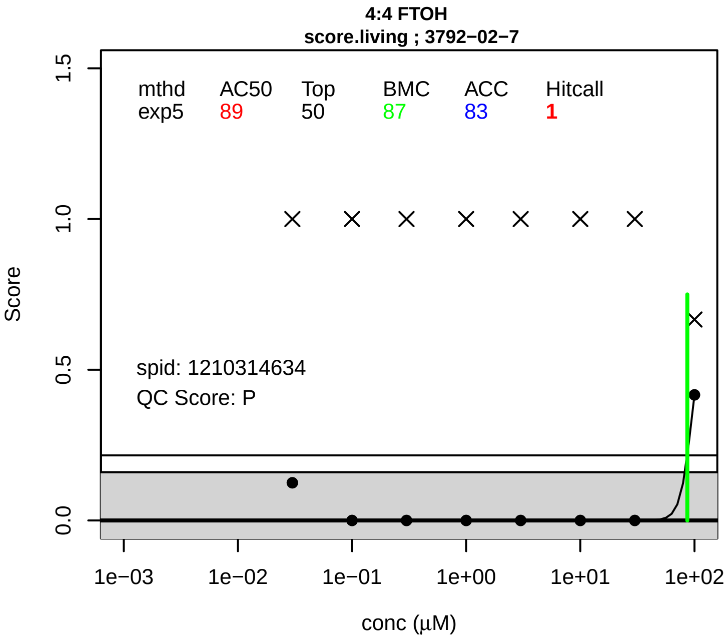
4:4 FTOH
score.living ; 3792−02−7
mthd AC50 Top BMC ACC Hitcall
exp5 89 50 87 83 1
spid: 1210314634
QC Score: P
Score
conc (µM)

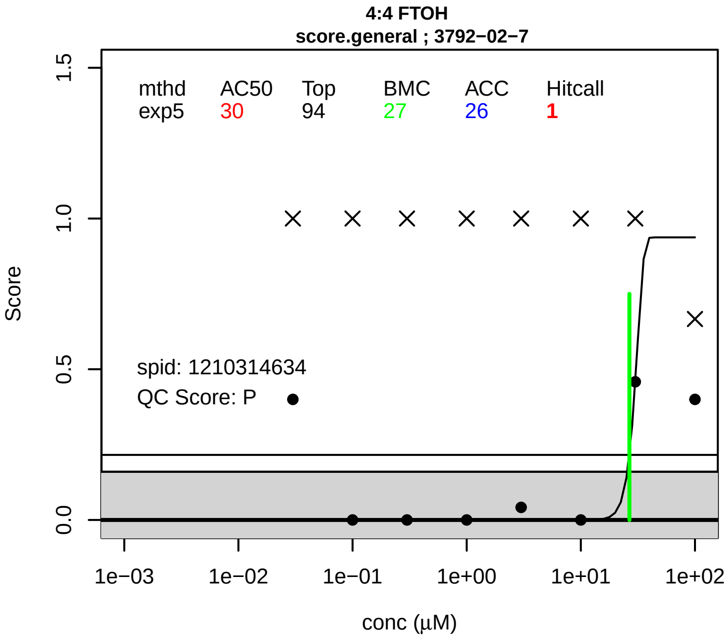
4:4 FTOH
score.general ; 3792−02−7
mthd AC50 Top BMC ACC Hitcall
exp5 30 94 27 26 1
spid: 1210314634
QC Score: P
Score
conc (µM)

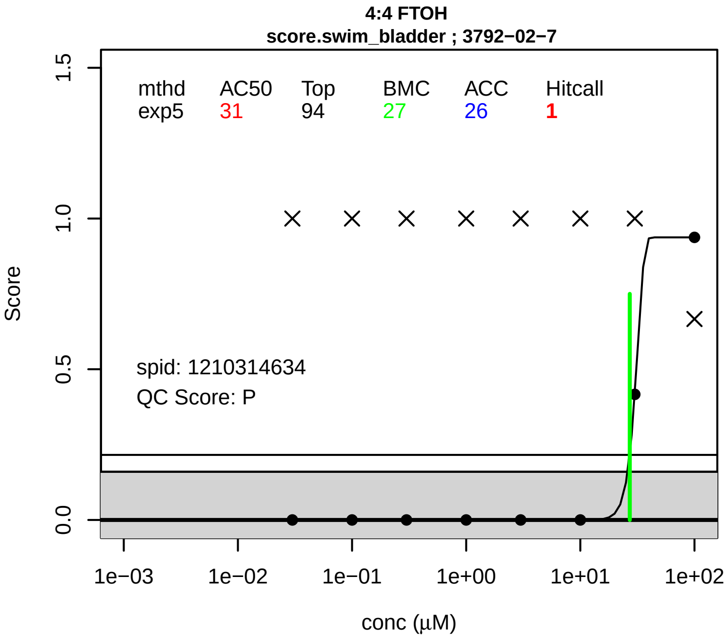
4:4 FTOH
score.swim_bladder ; 3792−02−7
mthd AC50 Top BMC ACC Hitcall
exp5 31 94 27 26 1
spid: 1210314634
QC Score: P
Score
conc (µM)

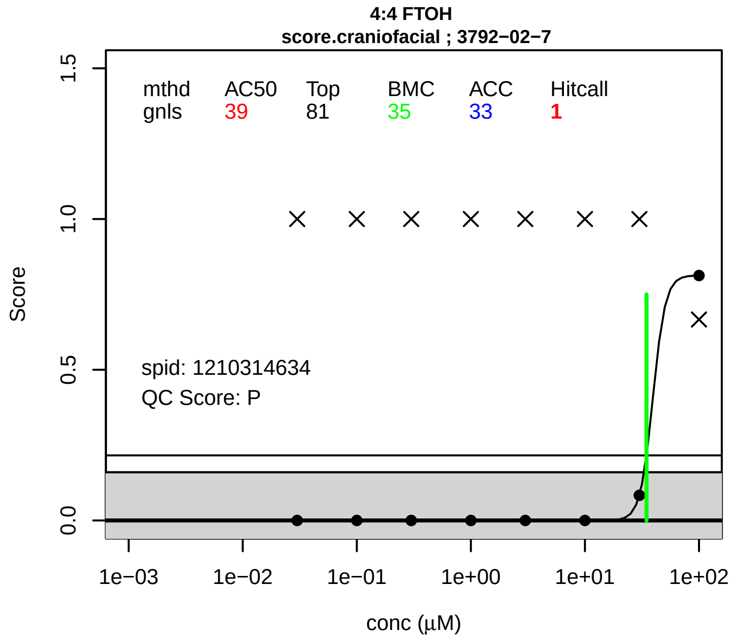
4:4 FTOH
score.craniofacial ; 3792−02−7
mthd gnls
AC50 39
Top 81
BMC 35
ACC 33
Hitcall 1
spid: 1210314634
QC Score: P
Score
conc (µM)

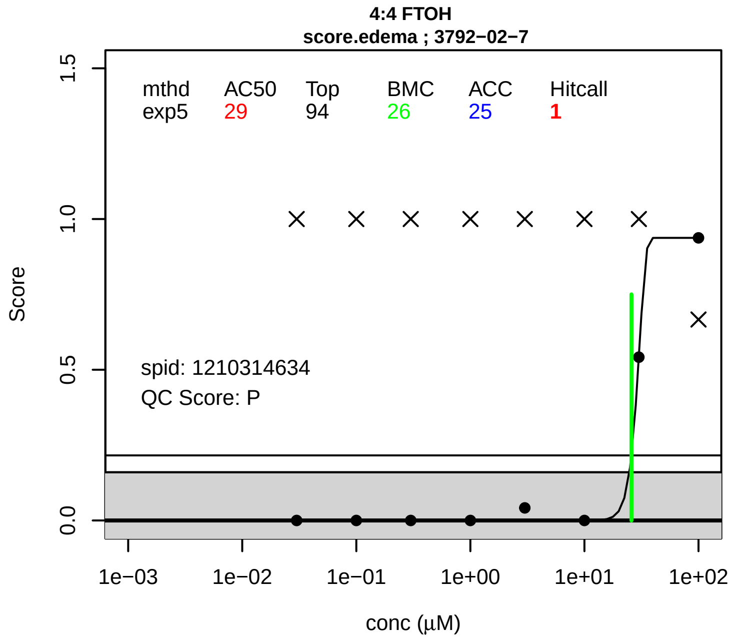
4:4 FTOH
score.edema ; 3792−02−7
mthd exp5
AC50 29
Top 94
BMC 26
ACC 25
Hitcall 1
spid: 1210314634
QC Score: P
Score
conc (µM)

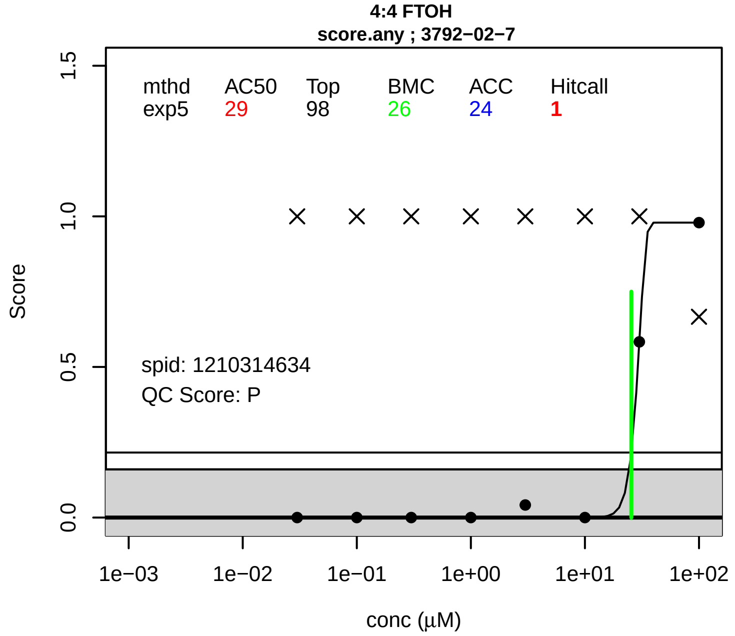
4:4 FTOH
score.any ; 3792−02−7
mthd exp5
AC50 29
Top 98
BMC 26
ACC 24
Hitcall 1
spid: 1210314634
QC Score: P
Score
conc (µM)

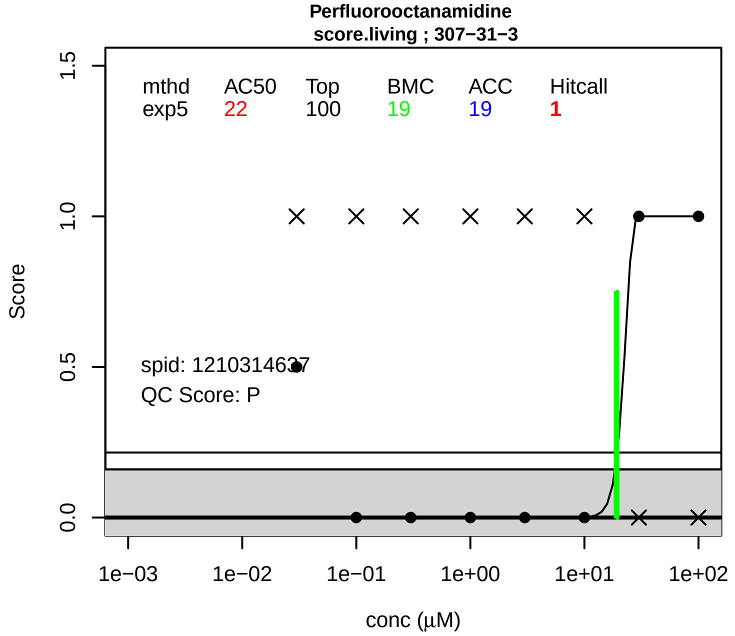
Perfluorooctanamidine
score.living ; 307−31−3
mthd exp5
AC50 22
Top 100
BMC 19
ACC 19
Hitcall 1
spid: 1210314637
QC Score: P
Score
conc (µM)

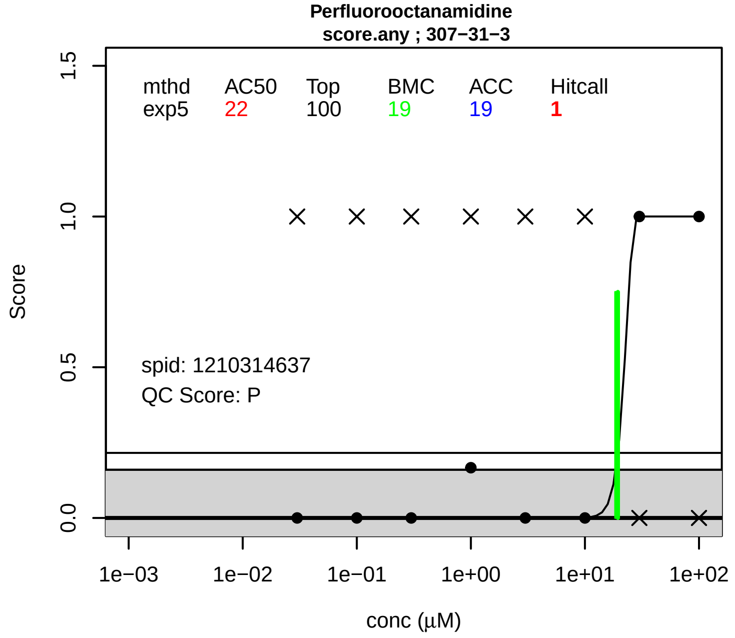
Perfluorooctanamidine
score.any ; 307−31−3
mthd exp5
AC50 22
Top 100
BMC 19
ACC 19
Hitcall 1
spid: 1210314637
QC Score: P
Score
conc (µM)

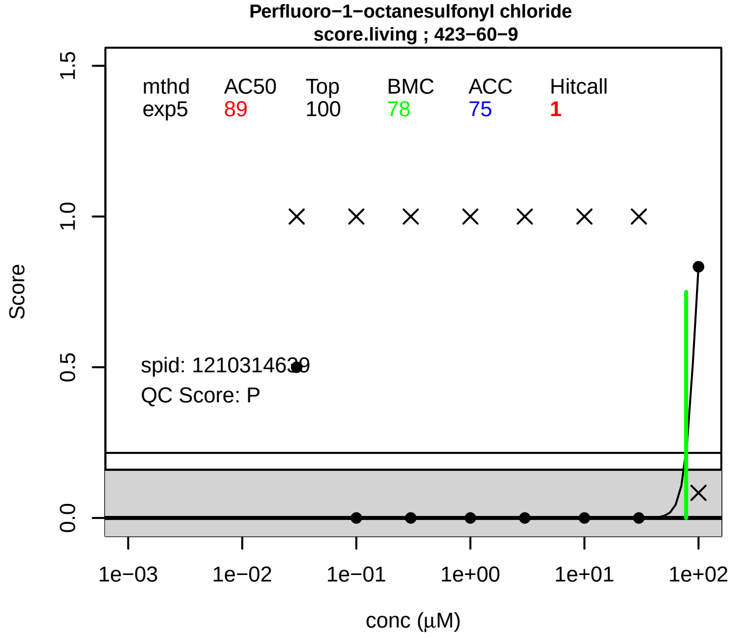
Perfluoro−1−octanesulfonyl chloride
score.living ; 423−60−9
mthd exp5
AC50 89
Top 100
BMC 78
ACC 75
Hitcall 1
spid: 1210314639
QC Score: P
Score
conc (µM)

Perfluoro−1−octanesulfonyl chloride
score.general ; 423−60−9

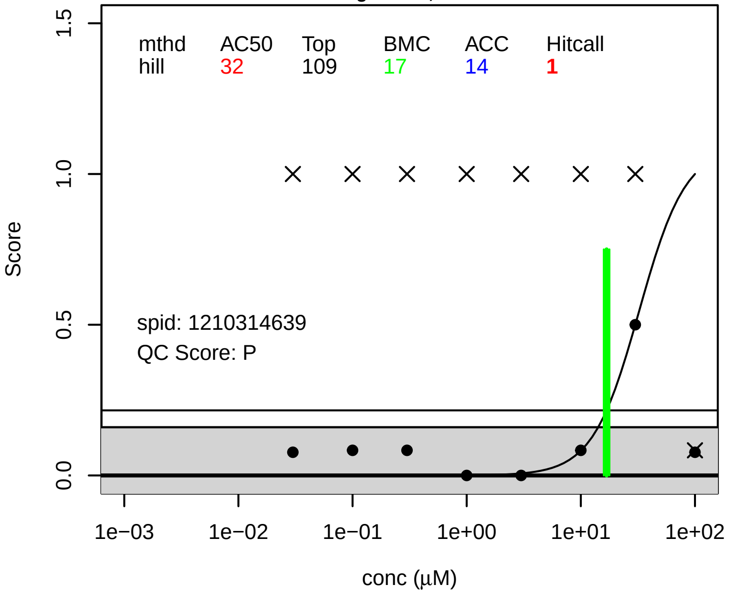

Perfluoro−1−octanesulfonyl chloride
score.swim_bladder ; 423−60−9

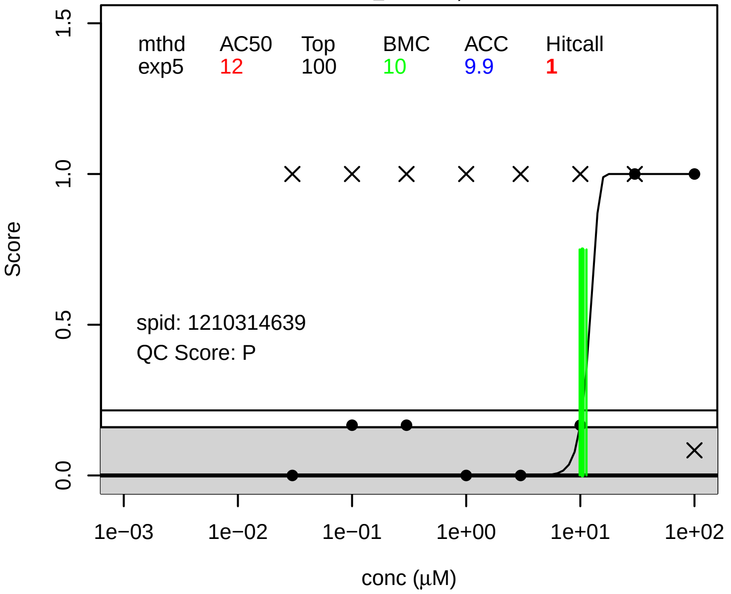

Perfluoro−1−octanesulfonyl chloride
score.edema ; 423−60−9

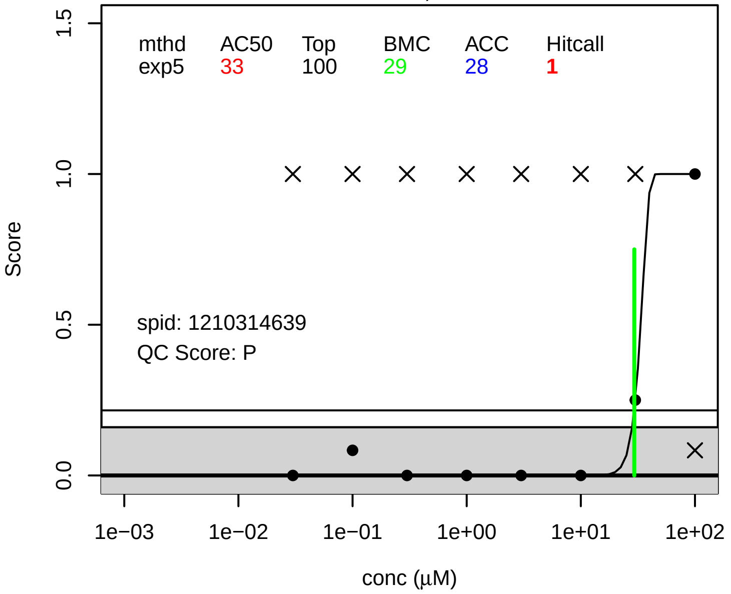

Perfluoro−1−octanesulfonyl chloride
score.spine ; 423−60−9

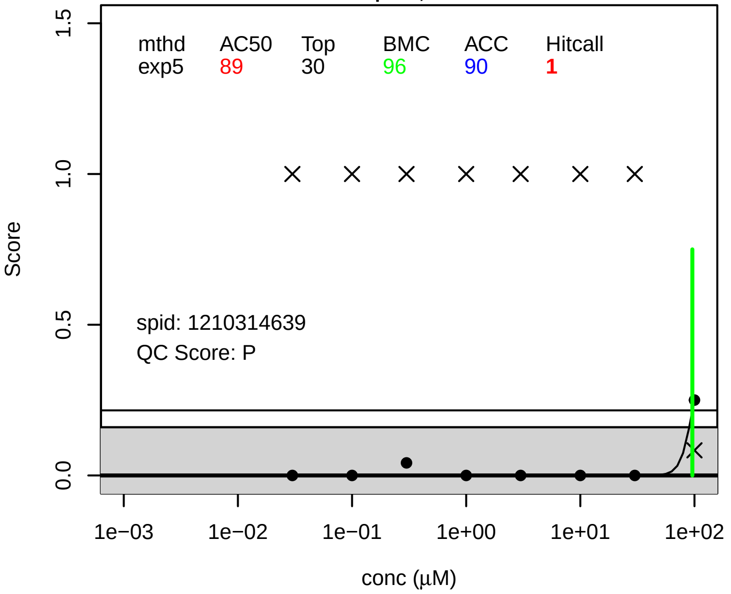

Perfluoro−1−octanesulfonyl chloride
score.position ; 423−60−9

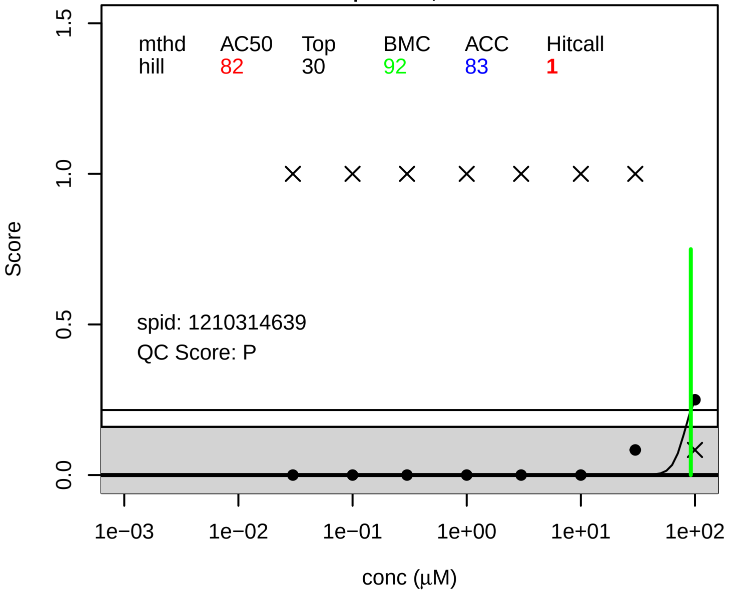

Perfluoro−1−octanesulfonyl chloride
score.any ; 423−60−9

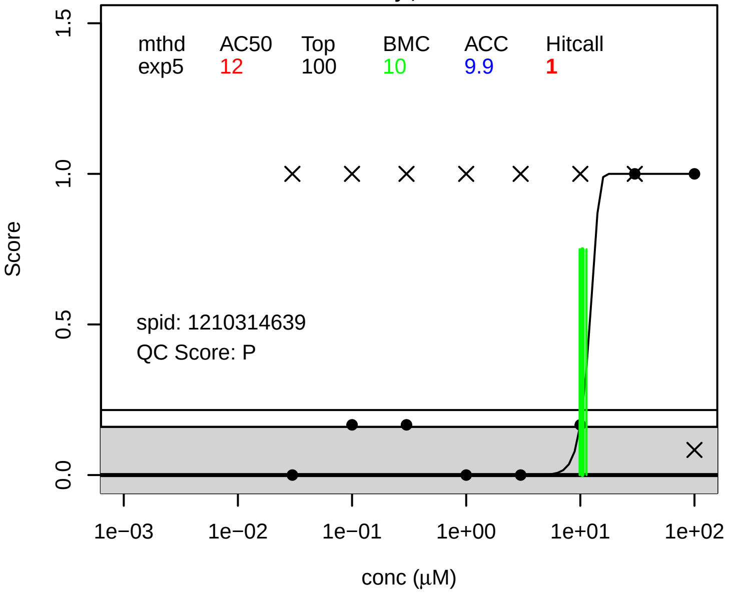

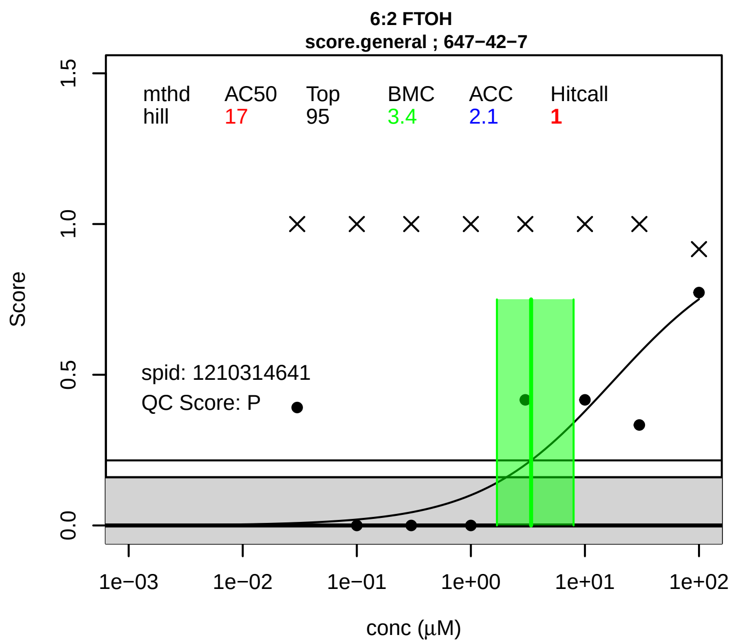
6:2 FTOH
score.general ; 647−42−7
mthd AC50 Top BMC ACC Hitcall
hill 17 95 3.4 2.1 1
spid: 1210314641
QC Score: P
Score
0.0 0.5 1.0 1.5
1e−03 1e−02 1e−01 1e+00 1e+01 1e+02
conc (µM)

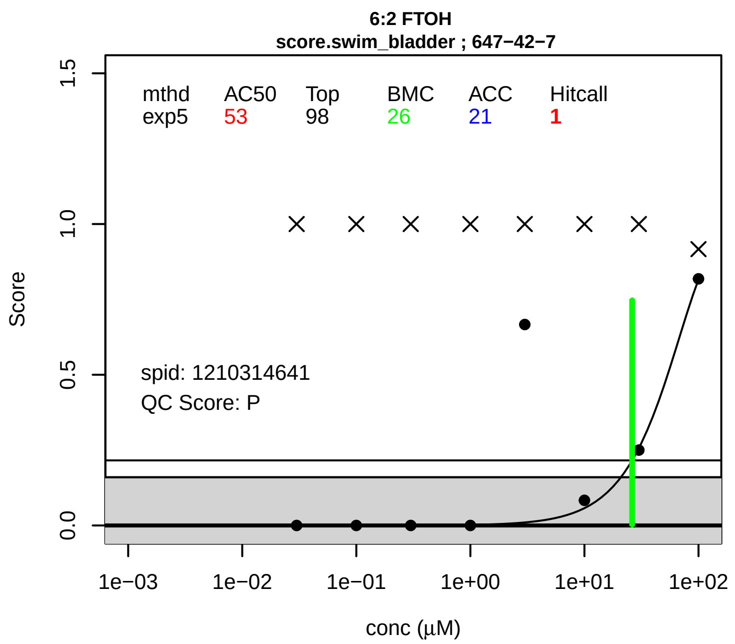
6:2 FTOH
score.swim_bladder ; 647−42−7
mthd AC50 Top BMC ACC Hitcall
exp5 53 98 26 21 1
spid: 1210314641
QC Score: P
Score
0.0 0.5 1.0 1.5
1e−03 1e−02 1e−01 1e+00 1e+01 1e+02
conc (µM)

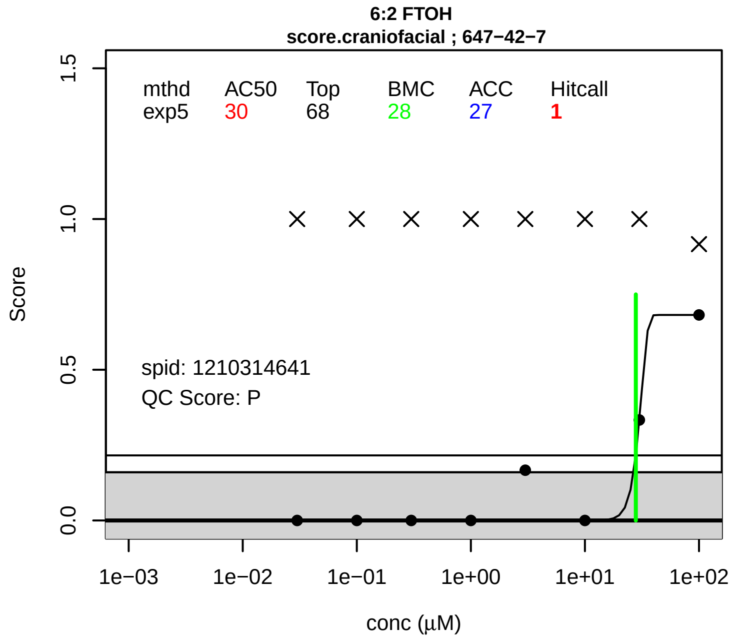
6:2 FTOH
score.craniofacial ; 647−42−7
mthd AC50 Top BMC ACC Hitcall
exp5 30 68 28 27 1
spid: 1210314641
QC Score: P
Score
0.0 0.5 1.0 1.5
1e−03 1e−02 1e−01 1e+00 1e+01 1e+02
conc (µM)

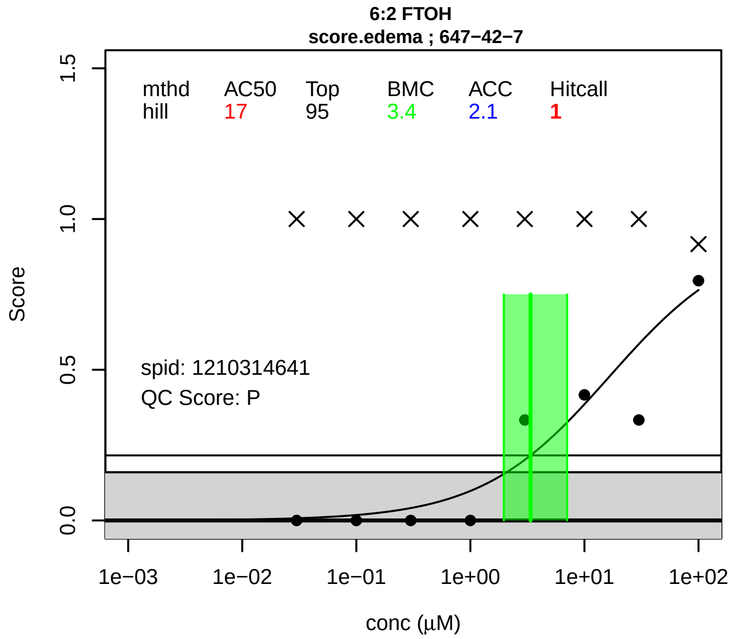
6:2 FTOH
score.edema ; 647−42−7
mthd AC50 Top BMC ACC Hitcall
hill 17 95 3.4 2.1 1
spid: 1210314641
QC Score: P
Score
0.0 0.5 1.0 1.5
1e−03 1e−02 1e−01 1e+00 1e+01 1e+02
conc (µM)

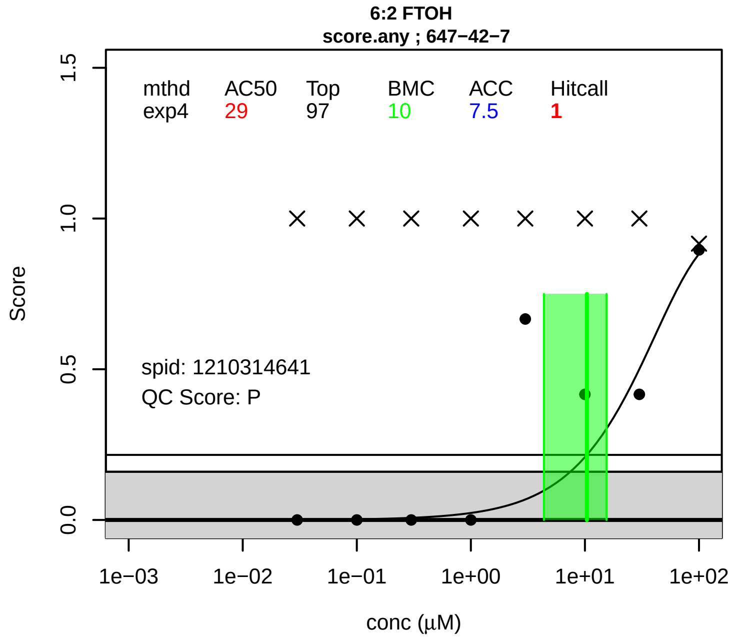
6:2 FTOH
score.any ; 647−42−7
mthd AC50 Top BMC ACC Hitcall
exp4 29 97 10 7.5 1
spid: 1210314641
QC Score: P
Score
0.0 0.5 1.0 1.5
1e−03 1e−02 1e−01 1e+00 1e+01 1e+02
conc (µM)

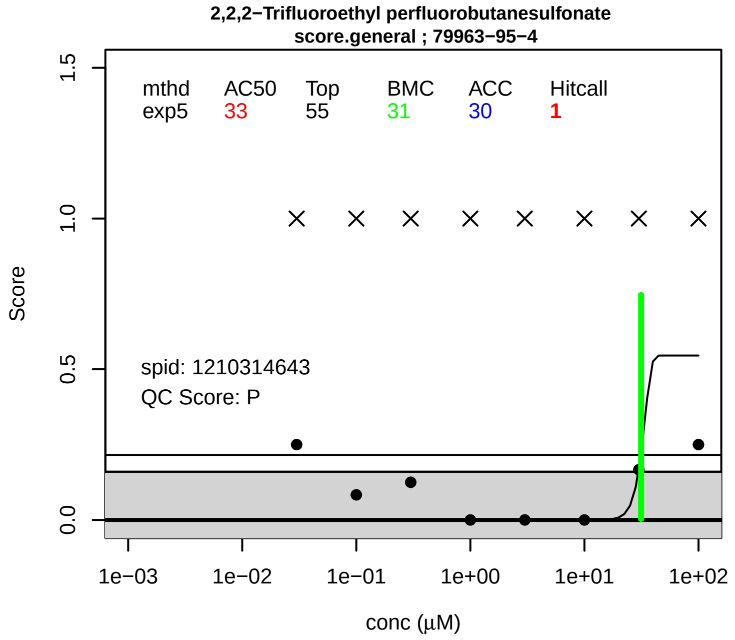
2,2,2−Trifluoroethyl perfluorobutanesulfonate
score.general ; 79963−95−4
mthd AC50 Top BMC ACC Hitcall
exp5 33 55 31 30 1
spid: 1210314643
QC Score: P
Score
0.0 0.5 1.0 1.5
1e−03 1e−02 1e−01 1e+00 1e+01 1e+02
conc (µM)

2,2,2−Trifluoroethyl perfluorobutanesulfonate
score.swim_bladder ; 79963−95−4

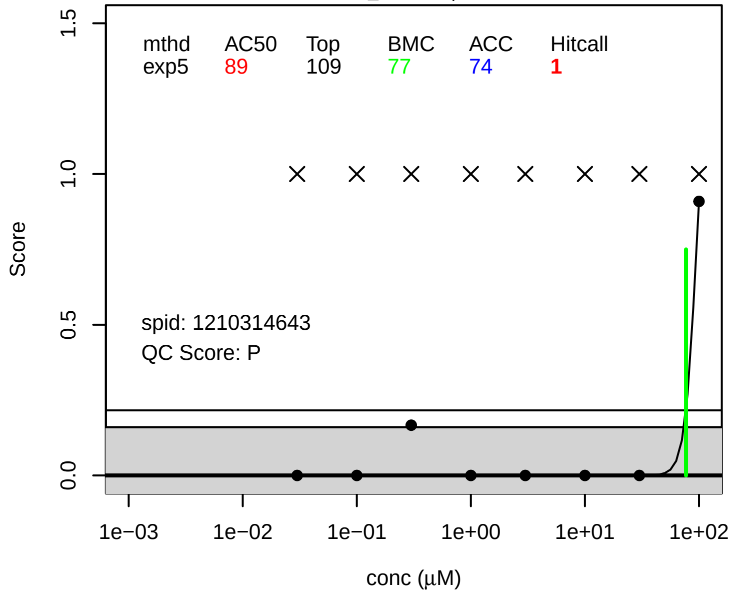

2,2,2−Trifluoroethyl perfluorobutanesulfonate
score.edema ; 79963−95−4

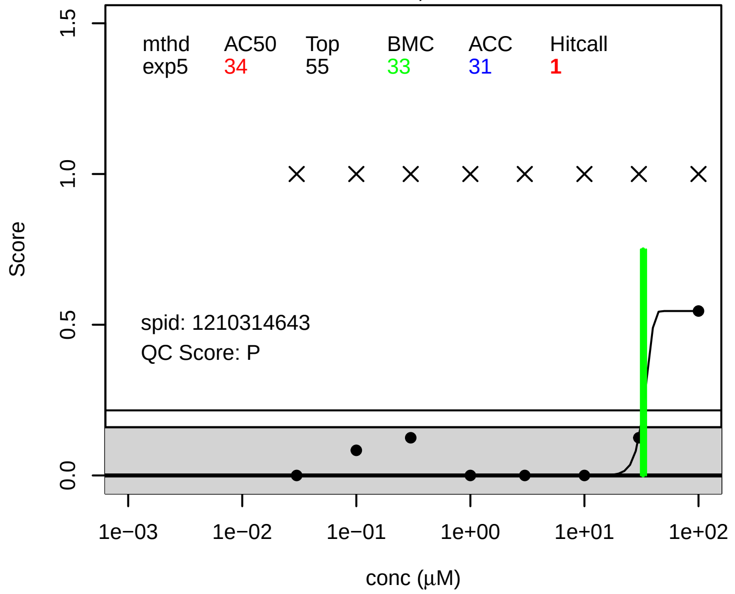

2,2,2−Trifluoroethyl perfluorobutanesulfonate
score.blood_pooling ; 79963−95−4

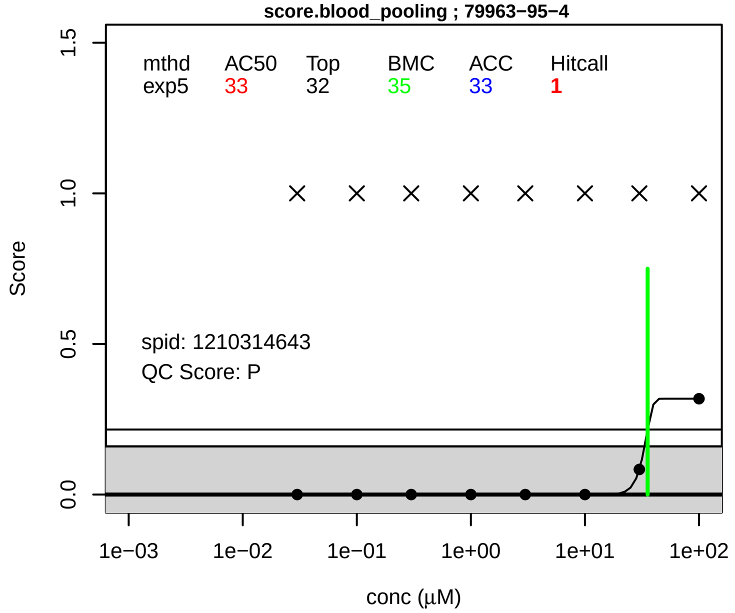

2,2,2−Trifluoroethyl perfluorobutanesulfonate
score.any ; 79963−95−4

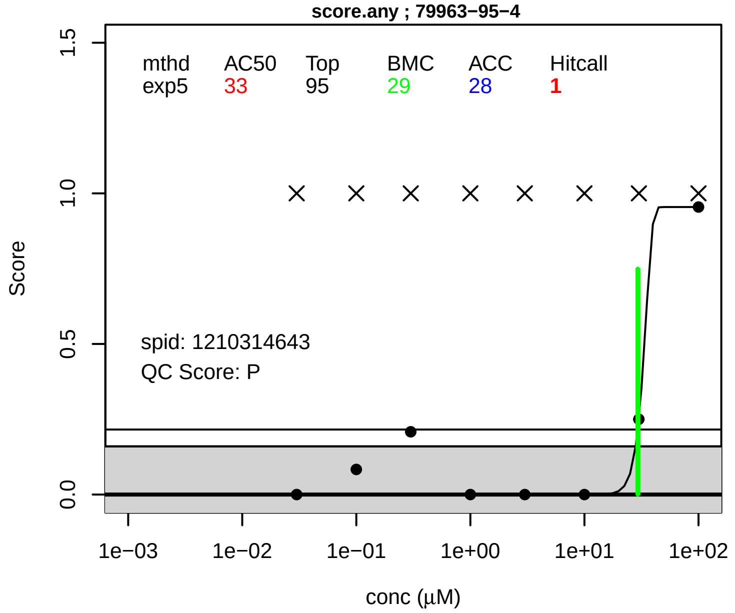

N−MeFOSA
score.general ; 31506−32−8

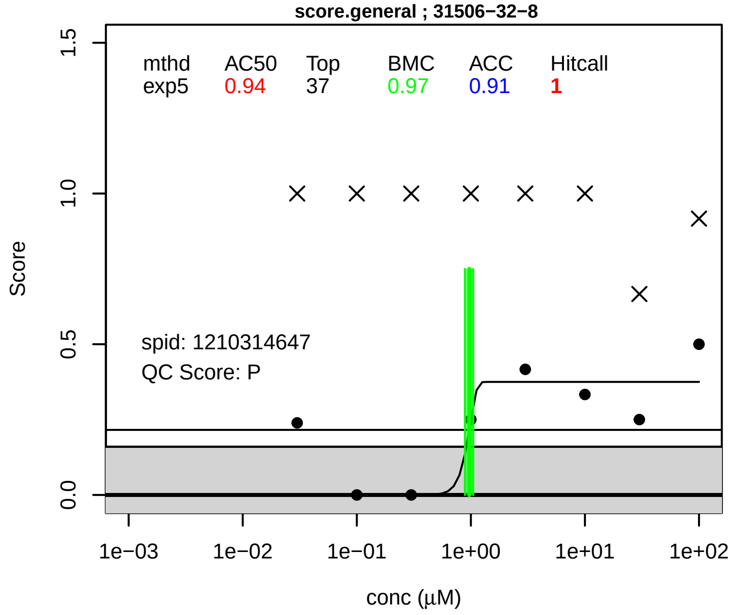

N−MeFOSA
score.swim_bladder ; 31506−32−8

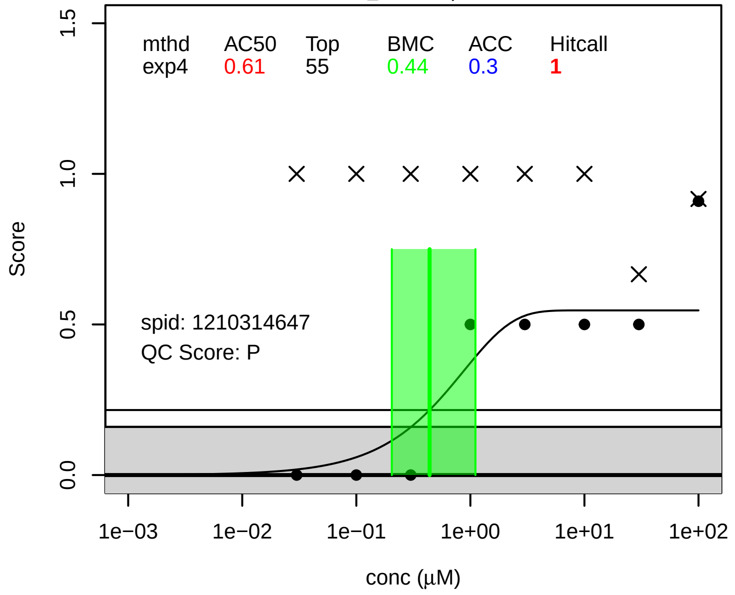

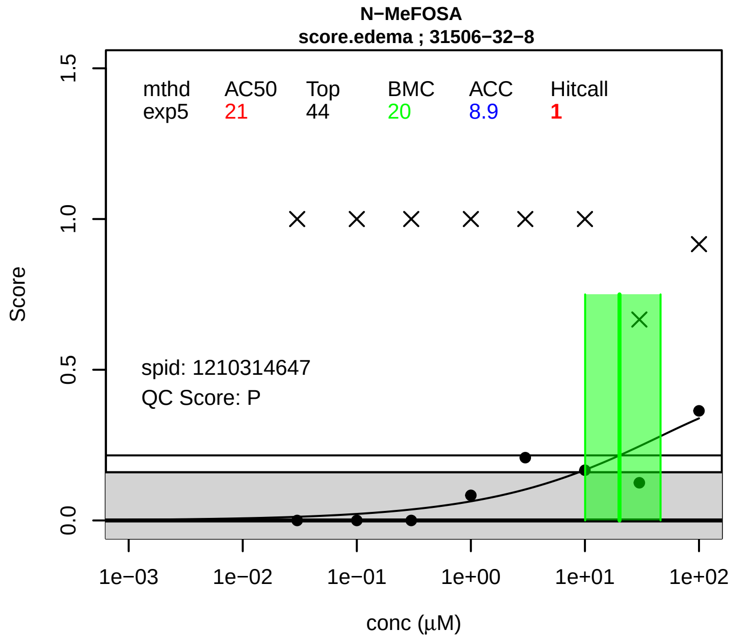
N−MeFOSA
score.edema ; 31506−32−8
mthd AC50 Top BMC ACC Hitcall
exp5 21 44 20 8.9 1
spid: 1210314647
QC Score: P
Score
conc (µM)

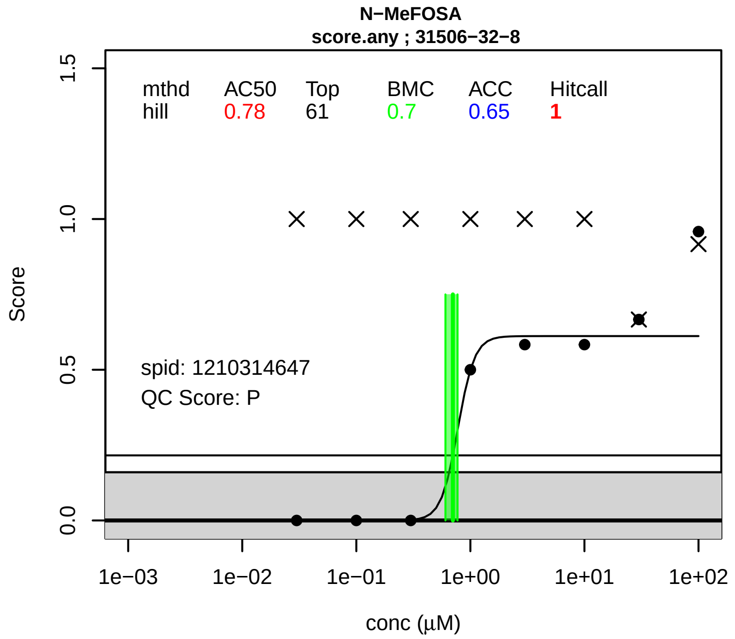
N−MeFOSA
score.any ; 31506−32−8
mthd AC50 Top BMC ACC Hitcall
hill 0.78 61 0.7 0.65 1
spid: 1210314647
QC Score: P
Score
conc (µM)

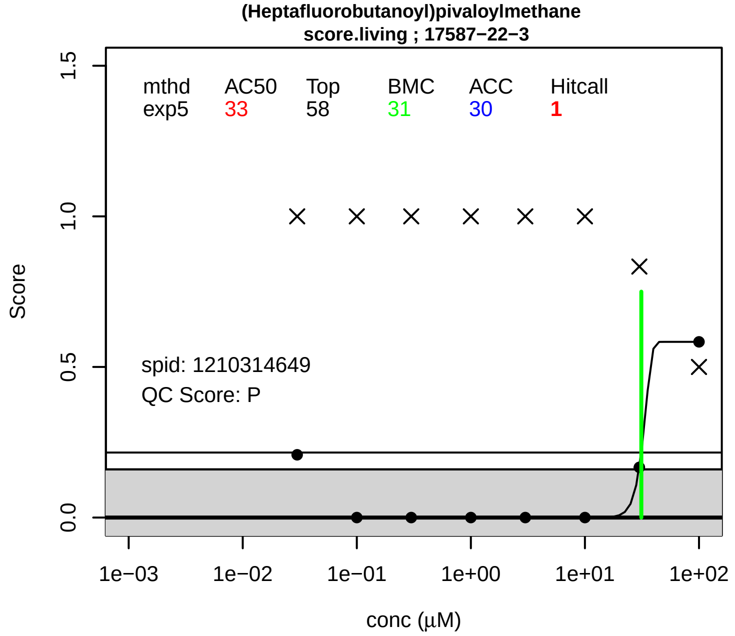
(Heptafluorobutanoyl)pivaloylmethane
score.living ; 17587−22−3
mthd AC50 Top BMC ACC Hitcall
exp5 33 58 31 30 1
spid: 1210314649
QC Score: P
Score
conc (µM)

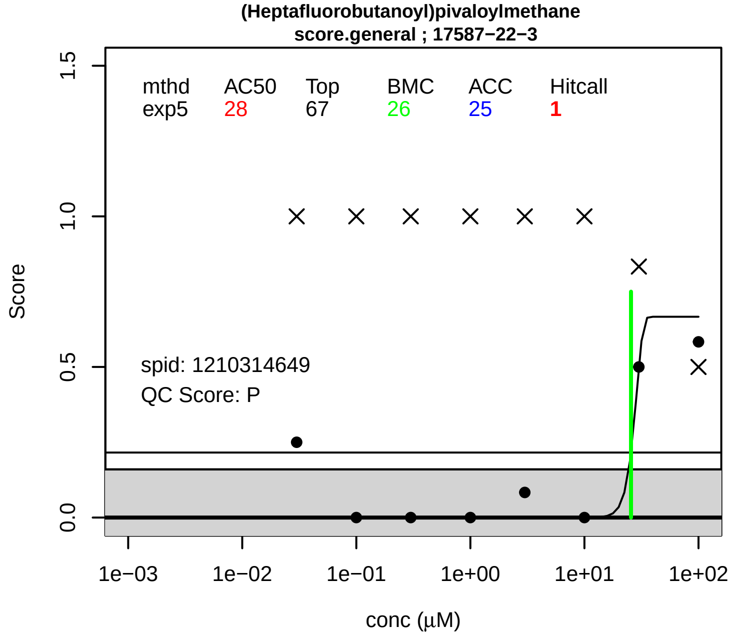
(Heptafluorobutanoyl)pivaloylmethane
score.general ; 17587−22−3
mthd AC50 Top BMC ACC Hitcall
exp5 28 67 26 25 1
spid: 1210314649
QC Score: P
Score
conc (µM)

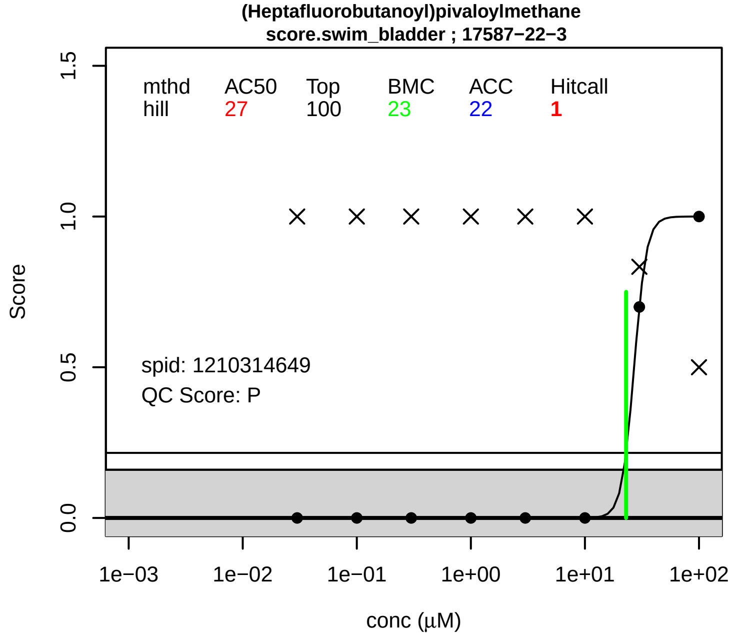
(Heptafluorobutanoyl)pivaloylmethane
score.swim_bladder ; 17587−22−3
mthd AC50 Top BMC ACC Hitcall
hill 27 100 23 22 1
spid: 1210314649
QC Score: P
Score
conc (µM)

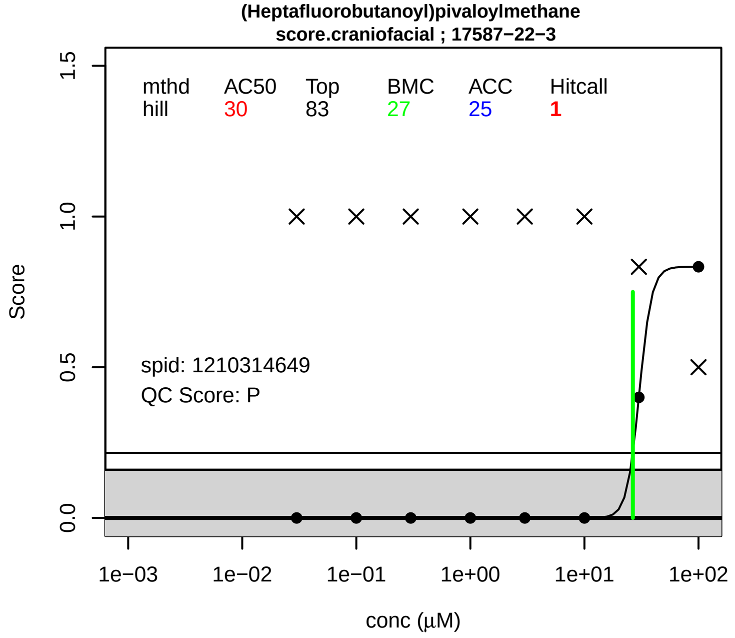
(Heptafluorobutanoyl)pivaloylmethane
score.craniofacial ; 17587−22−3
mthd AC50 Top BMC ACC Hitcall
hill 30 83 27 25 1
spid: 1210314649
QC Score: P
Score
conc (µM)

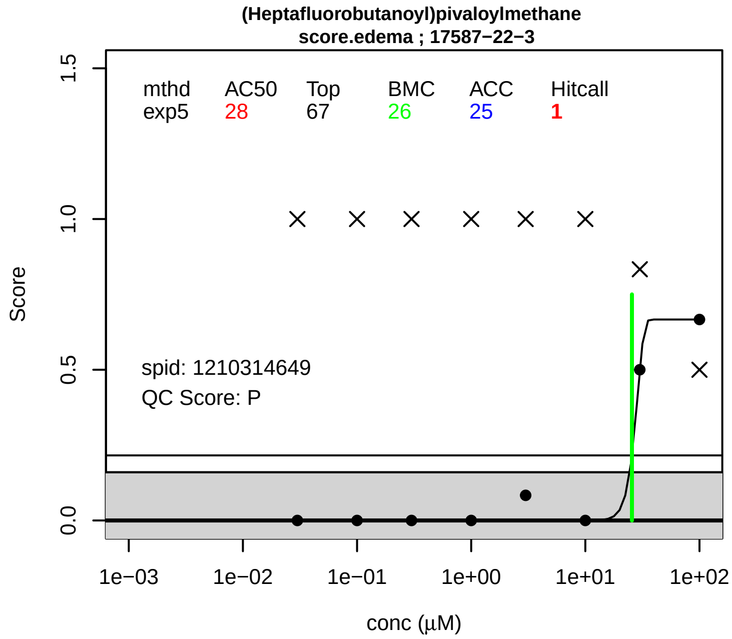
(Heptafluorobutanoyl)pivaloylmethane
score.edema ; 17587−22−3
mthd AC50 Top BMC ACC Hitcall
exp5 28 67 26 25 1
spid: 1210314649
QC Score: P
Score
conc (µM)
1.5
1.0
0.5
0.0
1e−03 1e−02 1e−01 1e+00 1e+01 1e+02

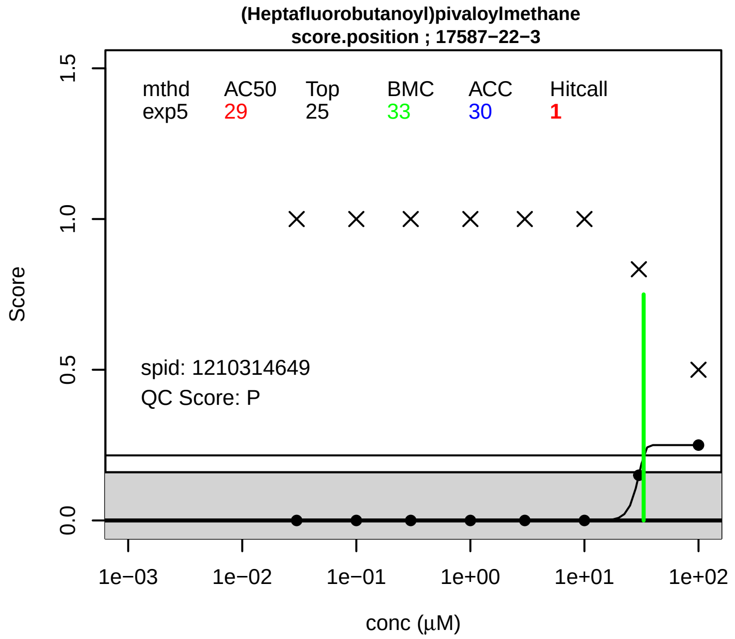
(Heptafluorobutanoyl)pivaloylmethane
score.position ; 17587−22−3
mthd AC50 Top BMC ACC Hitcall
exp5 29 25 33 30 1
spid: 1210314649
QC Score: P
Score
conc (µM)
1.5
1.0
0.5
0.0
1e−03 1e−02 1e−01 1e+00 1e+01 1e+02

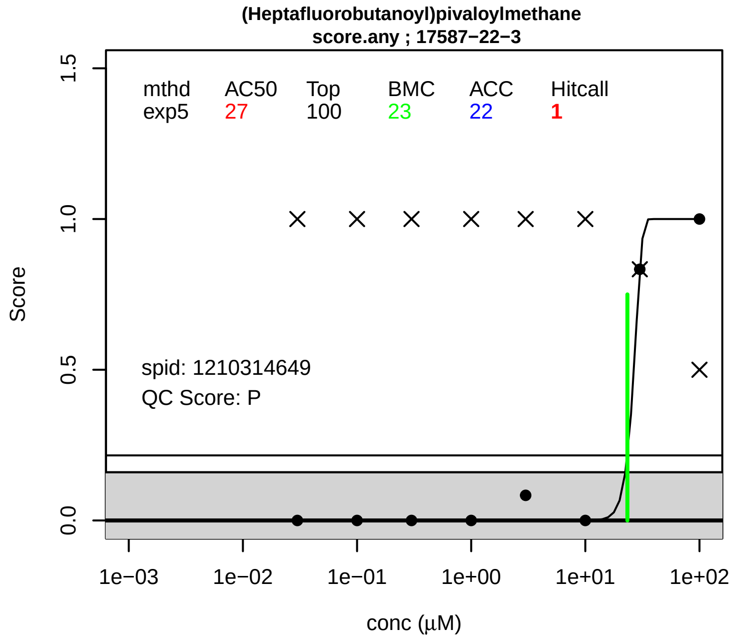
(Heptafluorobutanoyl)pivaloylmethane
score.any ; 17587−22−3
mthd AC50 Top BMC ACC Hitcall
exp5 27 100 23 22 1
spid: 1210314649
QC Score: P
Score
conc (µM)
1.5
1.0
0.5
0.0
1e−03 1e−02 1e−01 1e+00 1e+01 1e+02

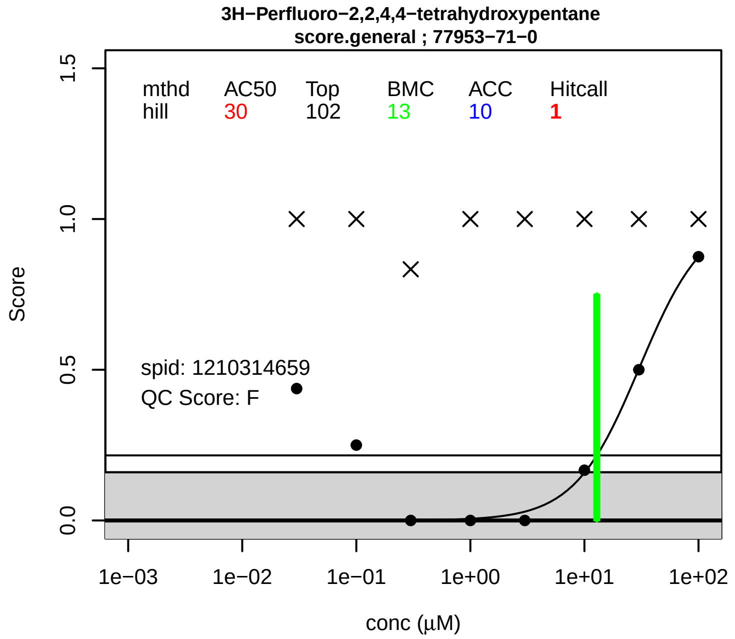
3H−Perfluoro−2,2,4,4−tetrahydroxypentane
score.general ; 77953−71−0
mthd AC50 Top BMC ACC Hitcall
hill 30 102 13 10 1
spid: 1210314659
QC Score: F
Score
conc (µM)
1.5
1.0
0.5
0.0
1e−03 1e−02 1e−01 1e+00 1e+01 1e+02

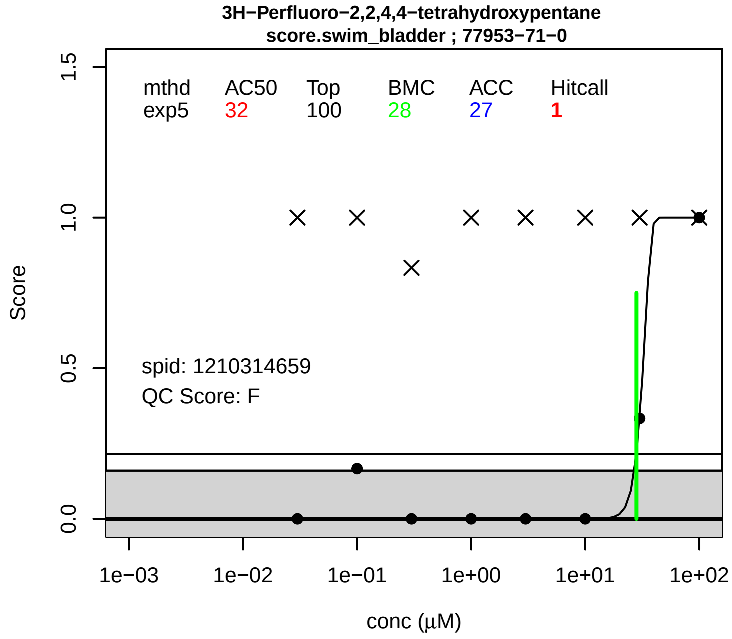
3H−Perfluoro−2,2,4,4−tetrahydroxypentane
score.swim_bladder ; 77953−71−0
mthd AC50 Top BMC ACC Hitcall
exp5 32 100 28 27 1
spid: 1210314659
QC Score: F
Score
conc (µM)
1.5
1.0
0.5
0.0
1e−03 1e−02 1e−01 1e+00 1e+01 1e+02

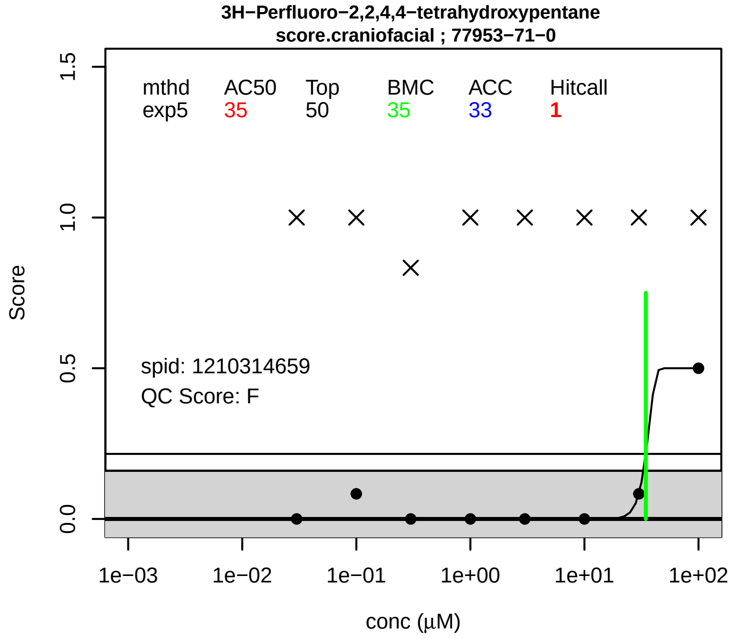
3H−Perfluoro−2,2,4,4−tetrahydroxypentane
score.craniofacial ; 77953−71−0
mthd AC50 Top BMC ACC Hitcall
exp5 35 50 35 33 1
spid: 1210314659
QC Score: F
Score
conc (µM)
1.5
1.0
0.5
0.0
1e−03 1e−02 1e−01 1e+00 1e+01 1e+02

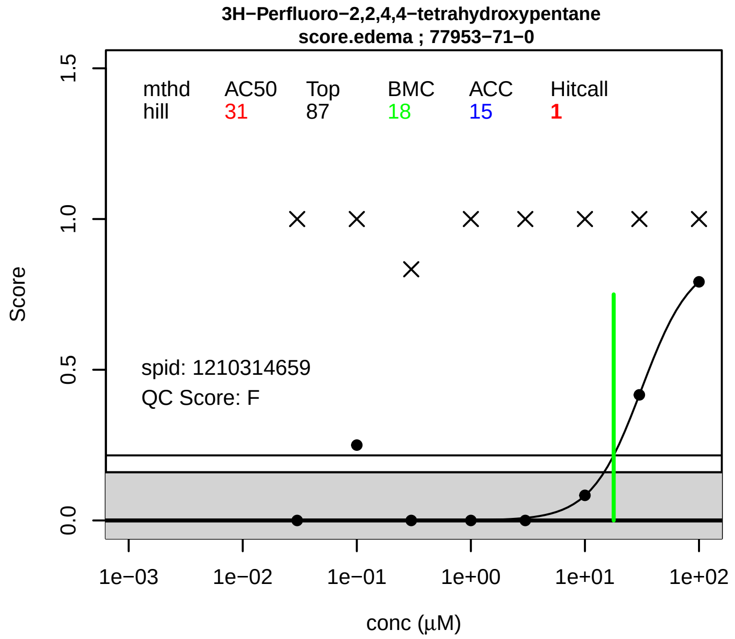
3H−Perfluoro−2,2,4,4−tetrahydroxypentane
score.edema ; 77953−71−0
mthd AC50 Top BMC ACC Hitcall
hill 31 87 18 15 1
spid: 1210314659
QC Score: F
Score
0.0 0.5 1.0 1.5
1e−03 1e−02 1e−01 1e+00 1e+01 1e+02
conc (µM)

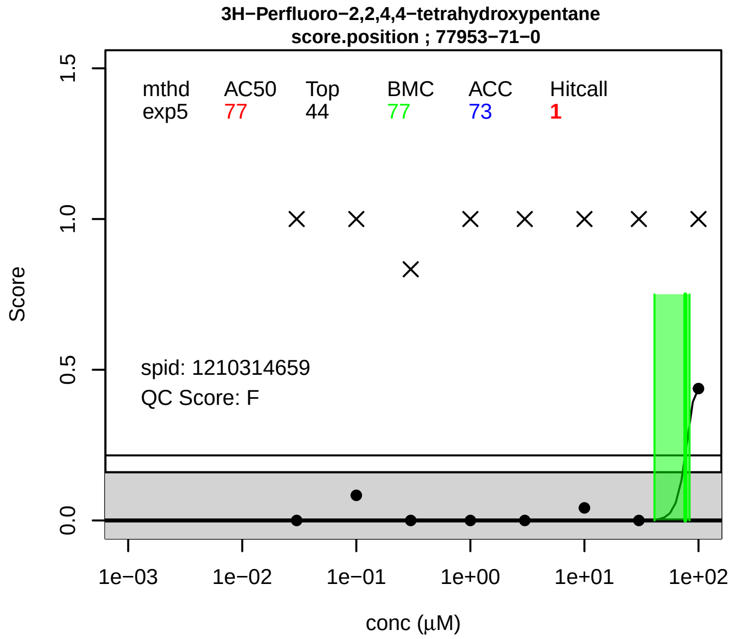
3H−Perfluoro−2,2,4,4−tetrahydroxypentane
score.position ; 77953−71−0
mthd AC50 Top BMC ACC Hitcall
exp5 77 44 77 73 1
spid: 1210314659
QC Score: F
Score
0.0 0.5 1.0 1.5
1e−03 1e−02 1e−01 1e+00 1e+01 1e+02
conc (µM)

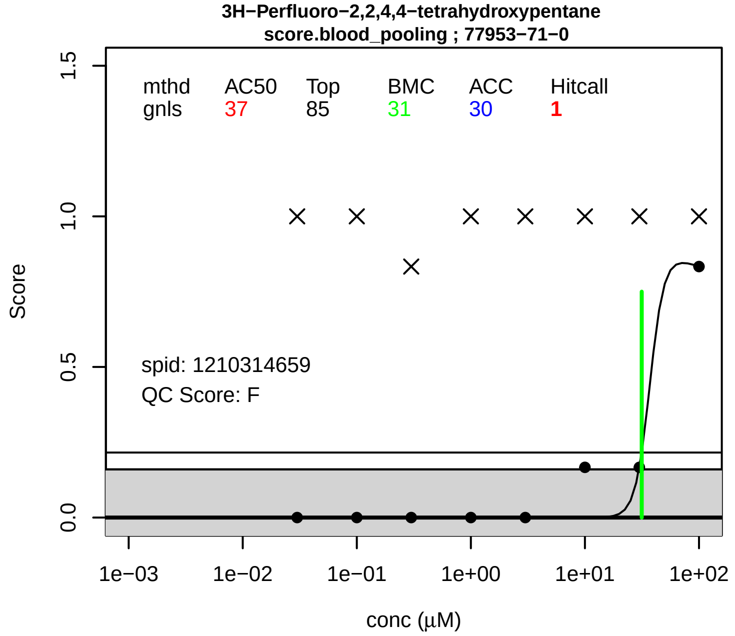
3H−Perfluoro−2,2,4,4−tetrahydroxypentane
score.blood_pooling ; 77953−71−0
mthd AC50 Top BMC ACC Hitcall
gnls 37 85 31 30 1
spid: 1210314659
QC Score: F
Score
0.0 0.5 1.0 1.5
1e−03 1e−02 1e−01 1e+00 1e+01 1e+02
conc (µM)

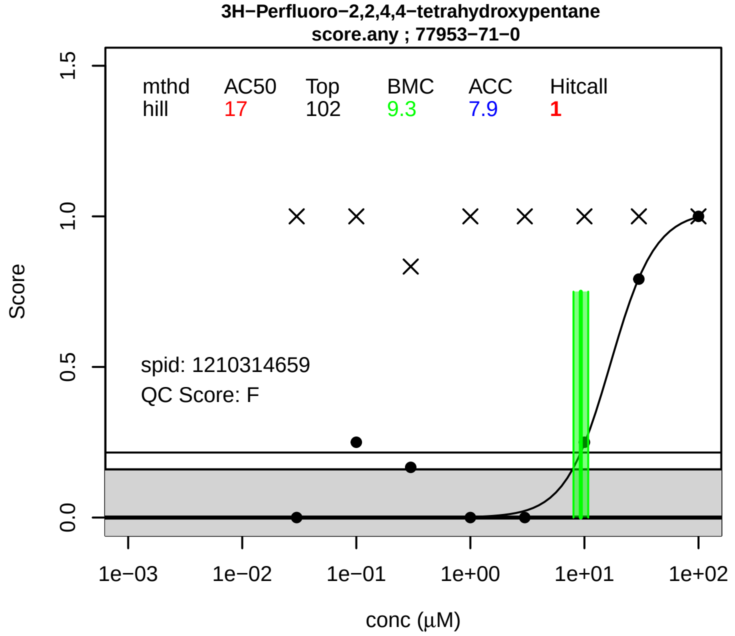
3H−Perfluoro−2,2,4,4−tetrahydroxypentane
score.any ; 77953−71−0
mthd AC50 Top BMC ACC Hitcall
hill 17 102 9.3 7.9 1
spid: 1210314659
QC Score: F
Score
0.0 0.5 1.0 1.5
1e−03 1e−02 1e−01 1e+00 1e+01 1e+02
conc (µM)

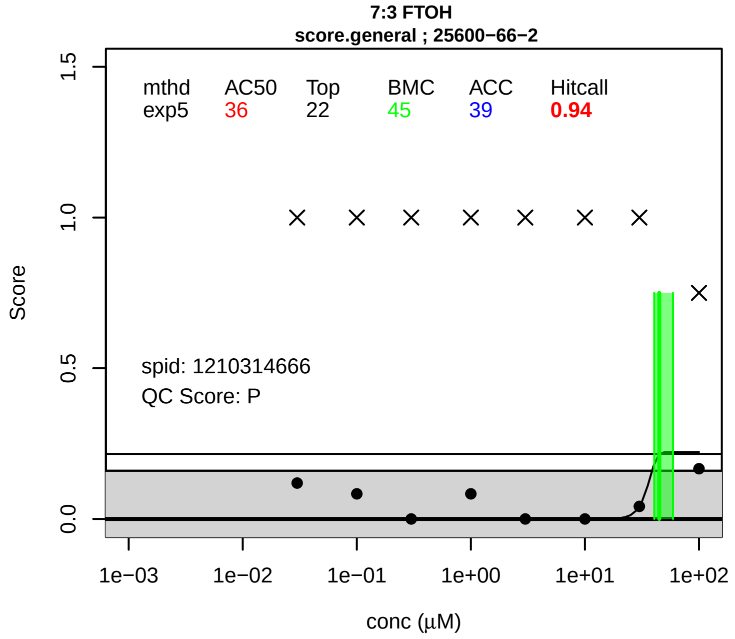
7:3 FTOH
score.general ; 25600−66−2
mthd AC50 Top BMC ACC Hitcall
exp5 36 22 45 39 0.94
spid: 1210314666
QC Score: P
Score
0.0 0.5 1.0 1.5
1e−03 1e−02 1e−01 1e+00 1e+01 1e+02
conc (µM)

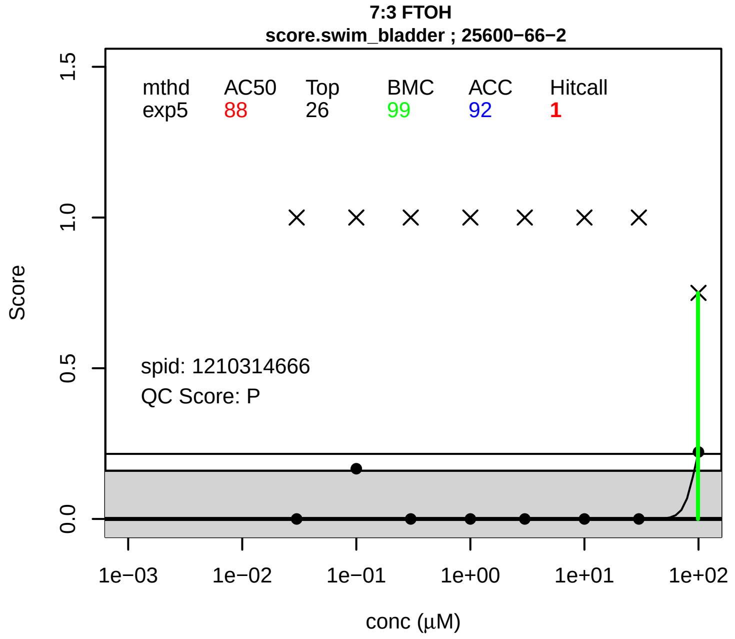
7:3 FTOH
score.swim_bladder ; 25600−66−2
mthd AC50 Top BMC ACC Hitcall
exp5 88 26 99 92 1
spid: 1210314666
QC Score: P
Score
0.0 0.5 1.0 1.5
1e−03 1e−02 1e−01 1e+00 1e+01 1e+02
conc (µM)

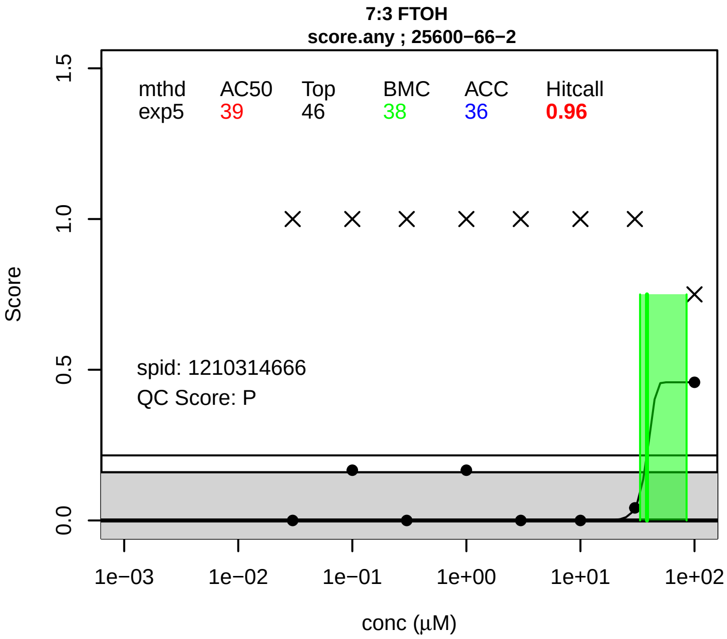
7:3 FTOH
score.any ; 25600−66−2
mthd AC50 Top BMC ACC Hitcall
exp5 39 46 38 36 0.96
spid: 1210314666
QC Score: P
Score
conc (µM)

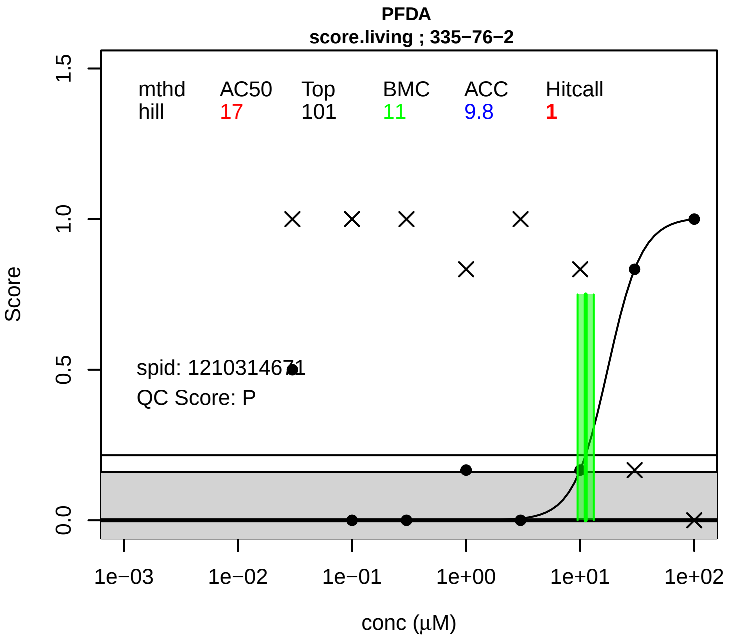
PFDA
score.living ; 335−76−2
mthd AC50 Top BMC ACC Hitcall
hill 17 101 11 9.8 1
spid: 1210314671
QC Score: P
Score
conc (µM)

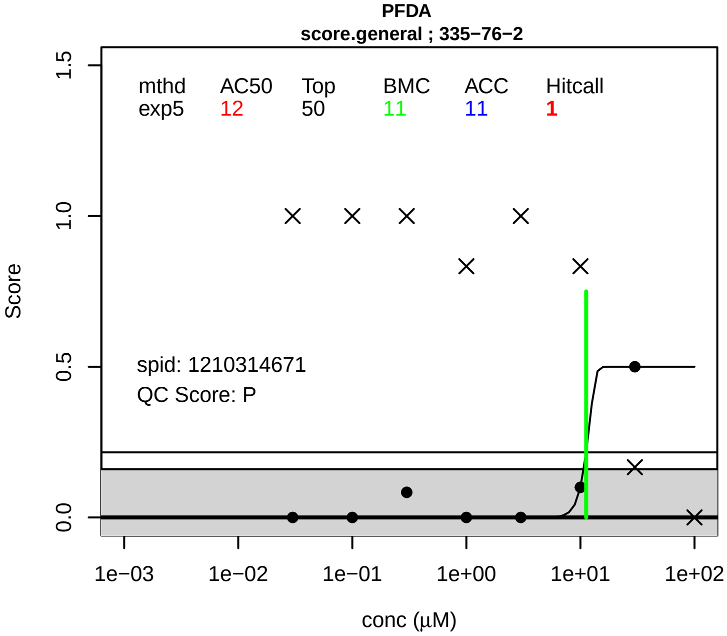
PFDA
score.general ; 335−76−2
mthd AC50 Top BMC ACC Hitcall
exp5 12 50 11 11 1
spid: 1210314671
QC Score: P
Score
conc (µM)

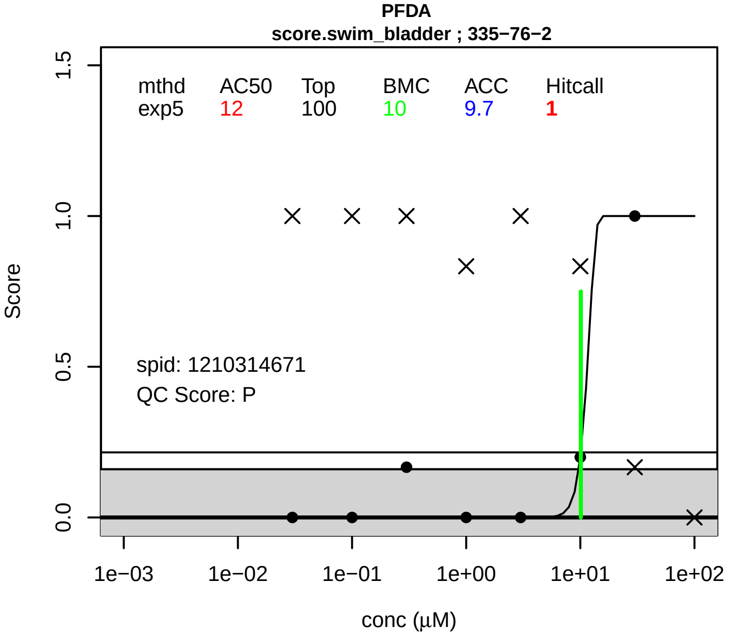
PFDA
score.swim_bladder ; 335−76−2
mthd AC50 Top BMC ACC Hitcall
exp5 12 100 10 9.7 1
spid: 1210314671
QC Score: P
Score
conc (µM)

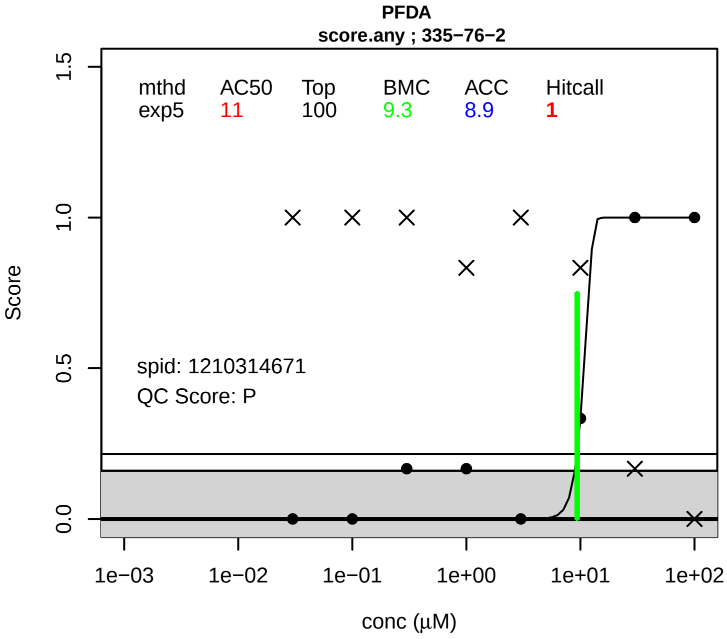
PFDA
score.any ; 335−76−2
mthd AC50 Top BMC ACC Hitcall
exp5 11 100 9.3 8.9 1
spid: 1210314671
QC Score: P
Score
conc (µM)

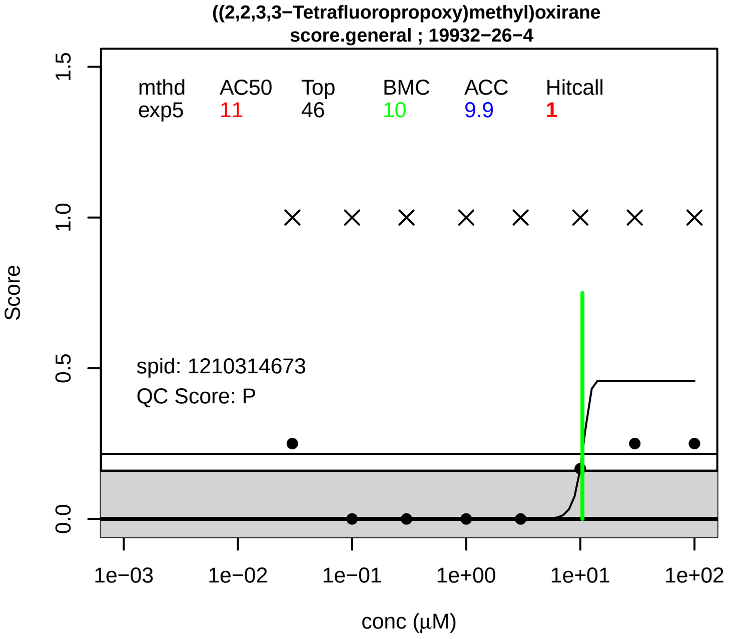
((2,2,3,3−Tetrafluoropropoxy)methyl)oxirane
score.general ; 19932−26−4
mthd AC50 Top BMC ACC Hitcall
exp5 11 46 10 9.9 1
spid: 1210314673
QC Score: P
Score
conc (µM)

((2,2,3,3−Tetrafluoropropoxy)methyl)oxirane
score.swim_bladder ; 19932−26−4

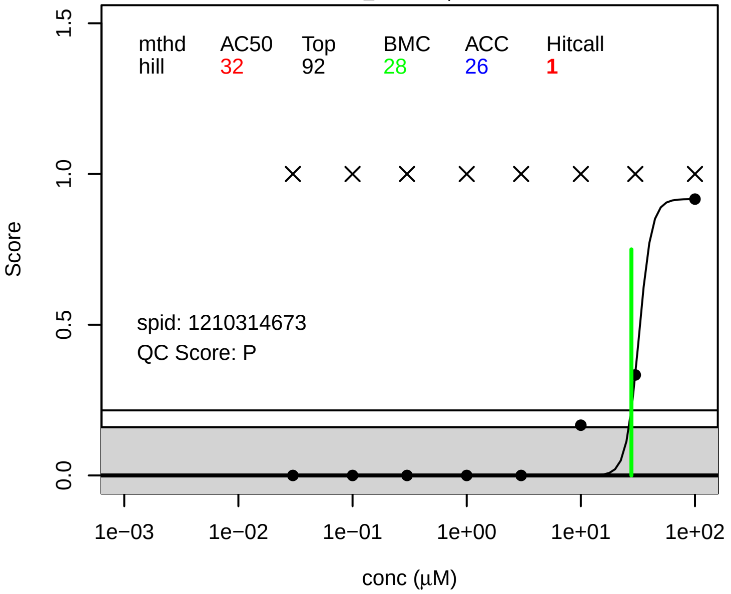

((2,2,3,3−Tetrafluoropropoxy)methyl)oxirane
score.edema ; 19932−26−4

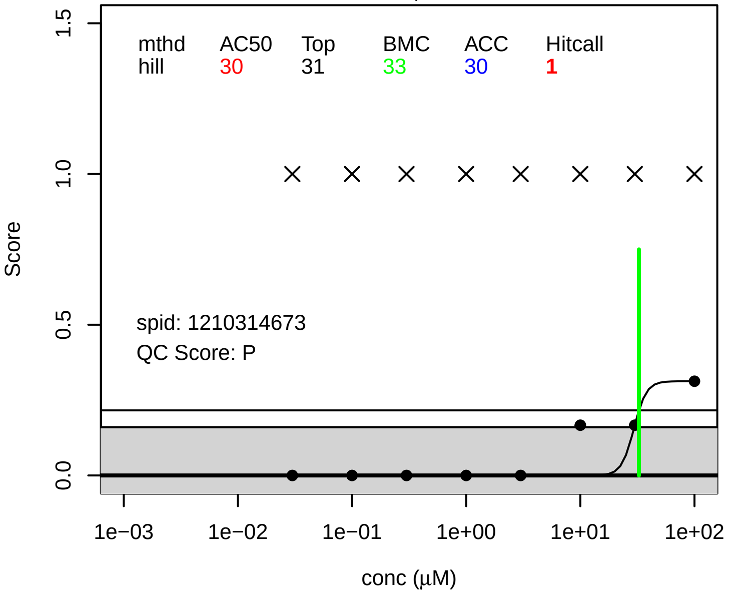

((2,2,3,3−Tetrafluoropropoxy)methyl)oxirane
score.any ; 19932−26−4

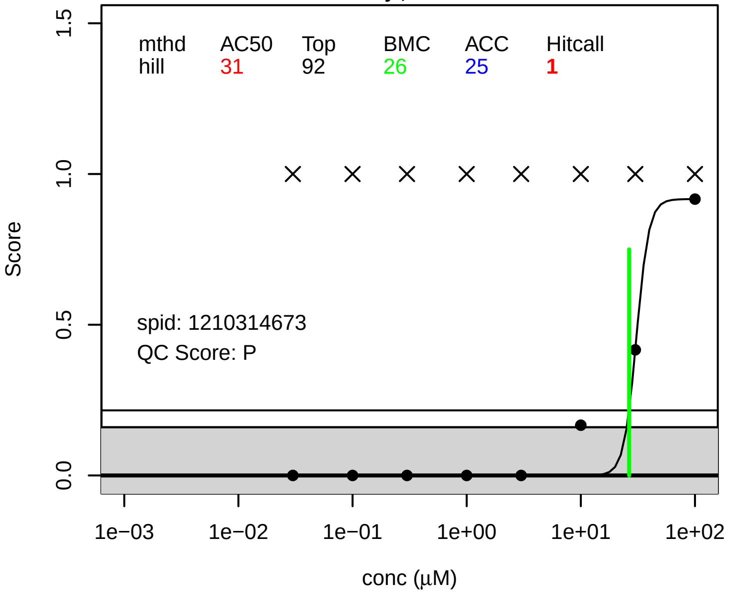

3:3 FTCA
score.any ; 356−02−5

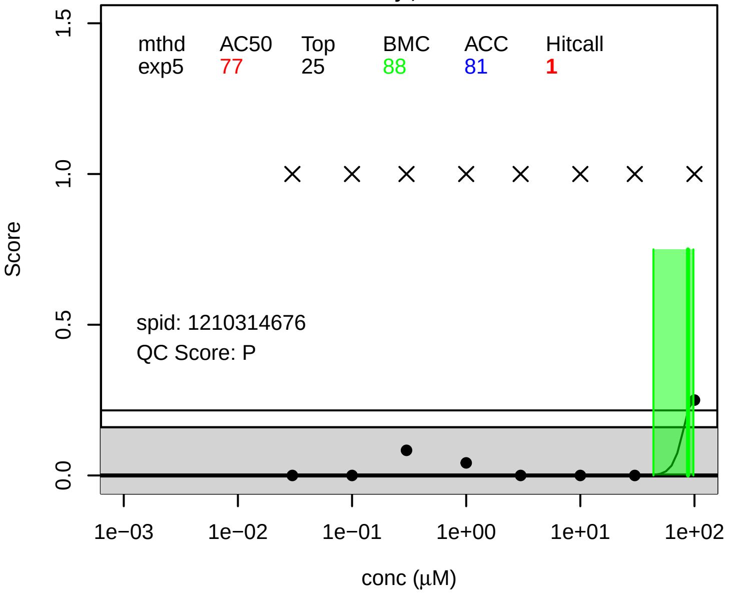

5:3 PFOA
score.living ; 914637−49−3

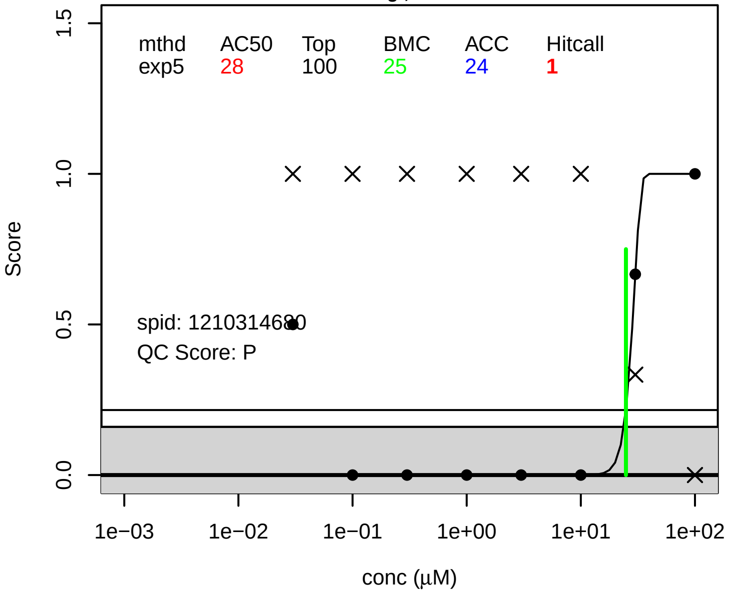

5:3 PFOA
score.general ; 914637−49−3

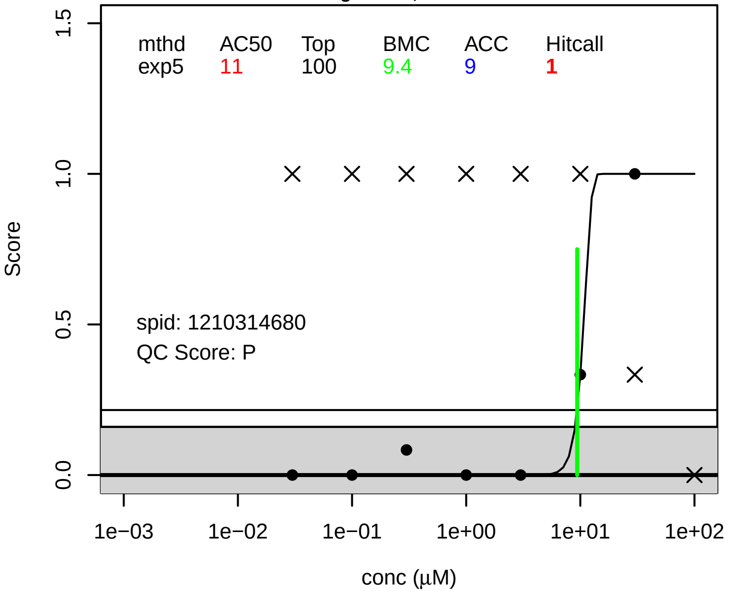

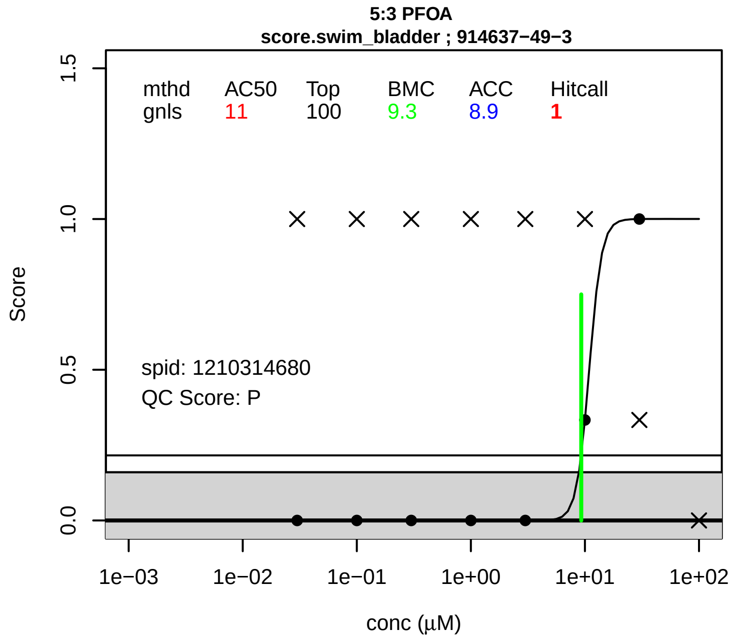
5:3 PFOA
score.swim_bladder ; 914637−49−3
mthd AC50 Top BMC ACC Hitcall
gnls 11 100 9.3 8.9 1
spid: 1210314680
QC Score: P
Score
conc (µM)

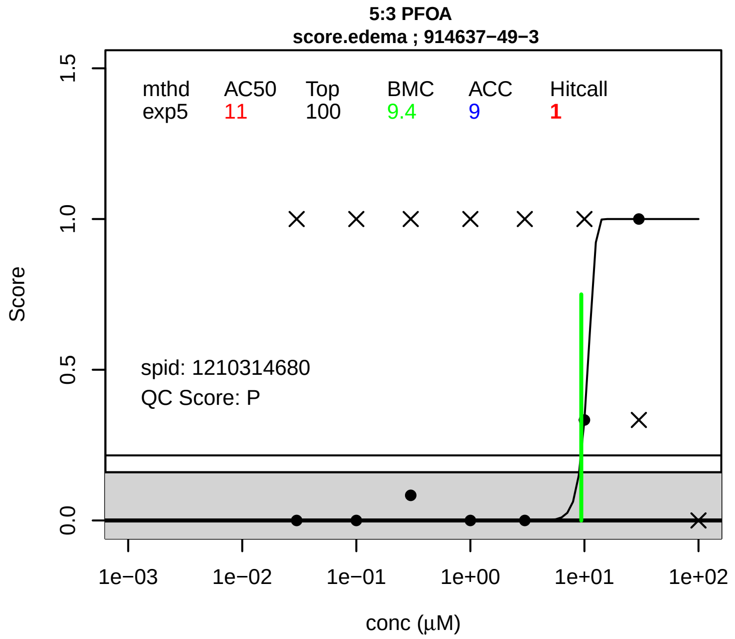
5:3 PFOA
score.edema ; 914637−49−3
mthd AC50 Top BMC ACC Hitcall
exp5 11 100 9.4 9 1
spid: 1210314680
QC Score: P
Score
conc (µM)

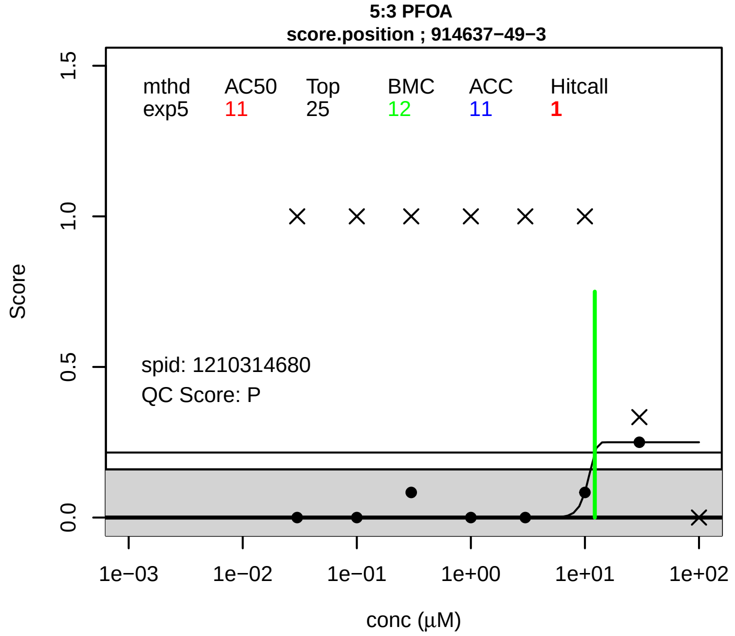
5:3 PFOA
score.position ; 914637−49−3
mthd AC50 Top BMC ACC Hitcall
exp5 11 25 12 11 1
spid: 1210314680
QC Score: P
Score
conc (µM)

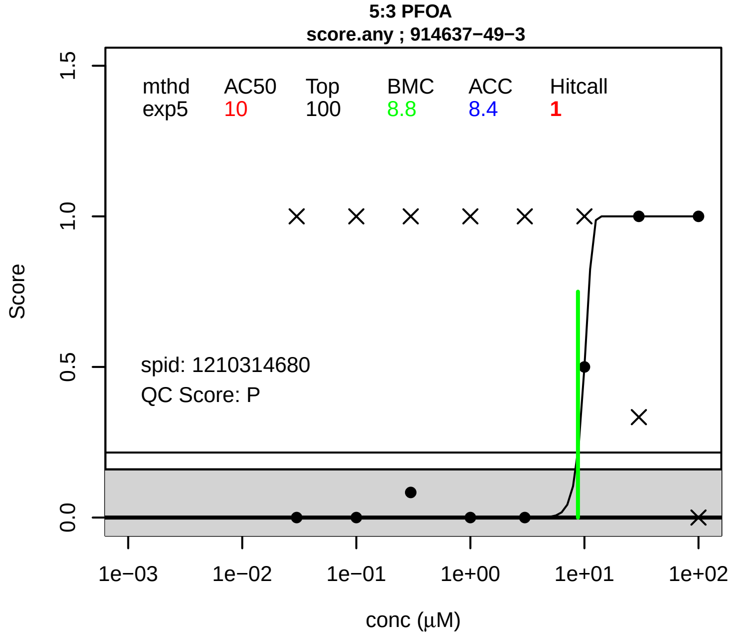
5:3 PFOA
score.any ; 914637−49−3
mthd AC50 Top BMC ACC Hitcall
exp5 10 100 8.8 8.4 1
spid: 1210314680
QC Score: P
Score
conc (µM)

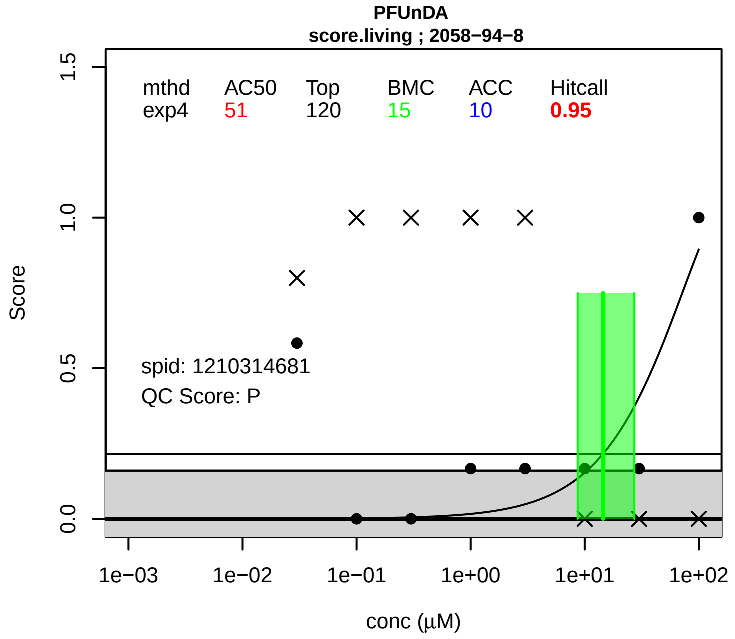
PFUnDA
score.living ; 2058−94−8
mthd AC50 Top BMC ACC Hitcall
exp4 51 120 15 10 0.95
spid: 1210314681
QC Score: P
Score
conc (µM)

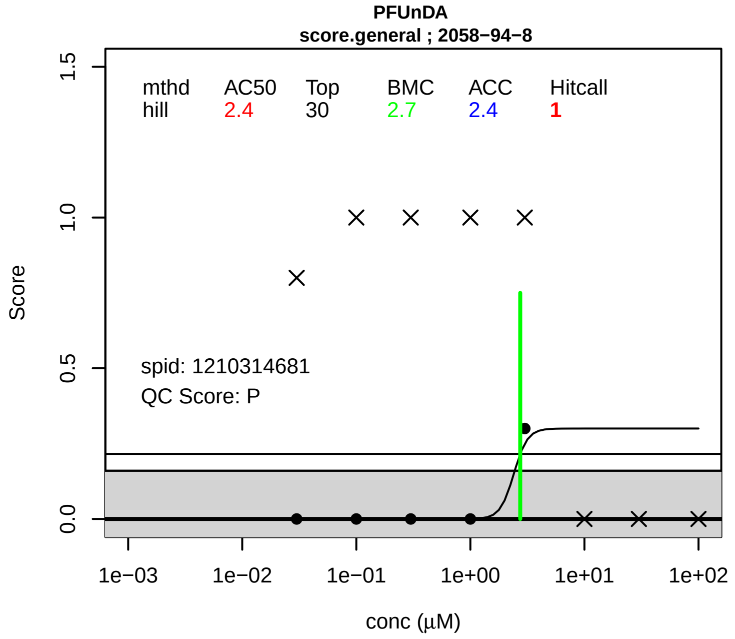
PFUnDA
score.general ; 2058−94−8
mthd AC50 Top BMC ACC Hitcall
hill 2.4 30 2.7 2.4 1
spid: 1210314681
QC Score: P
Score
conc (µM)

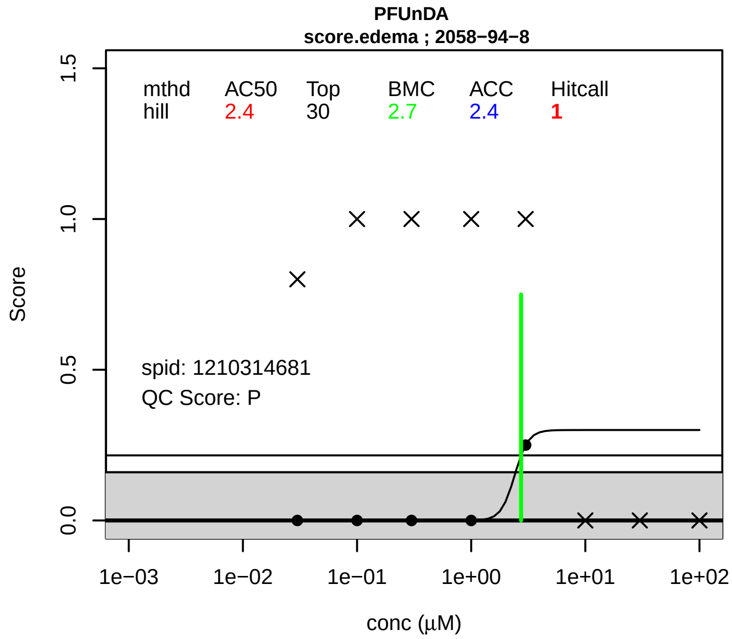
PFUnDA
score.edema ; 2058−94−8
mthd AC50 Top BMC ACC Hitcall
hill 2.4 30 2.7 2.4 1
spid: 1210314681
QC Score: P
Score
conc (µM)

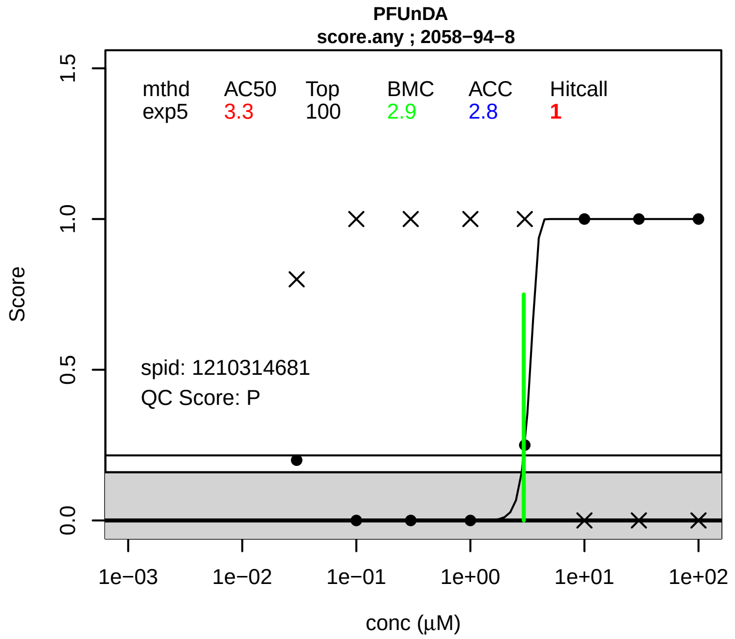
PFUnDA
score.any ; 2058−94−8
mthd AC50 Top BMC ACC Hitcall
exp5 3.3 100 2.9 2.8 1
spid: 1210314681
QC Score: P
Score
conc (µM)

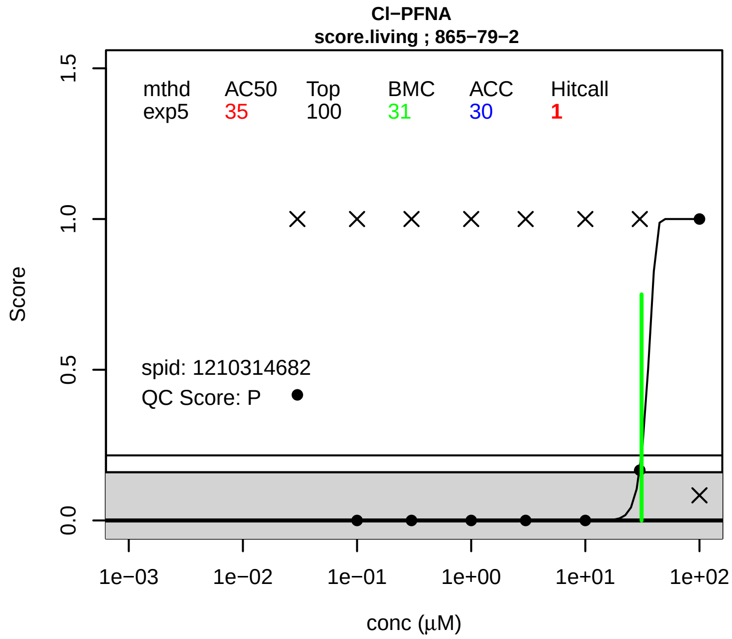
Cl−PFNA
score.living ; 865−79−2
mthd AC50 Top BMC ACC Hitcall
exp5 35 100 31 30 1
spid: 1210314682
QC Score: P
Score
conc (µM)

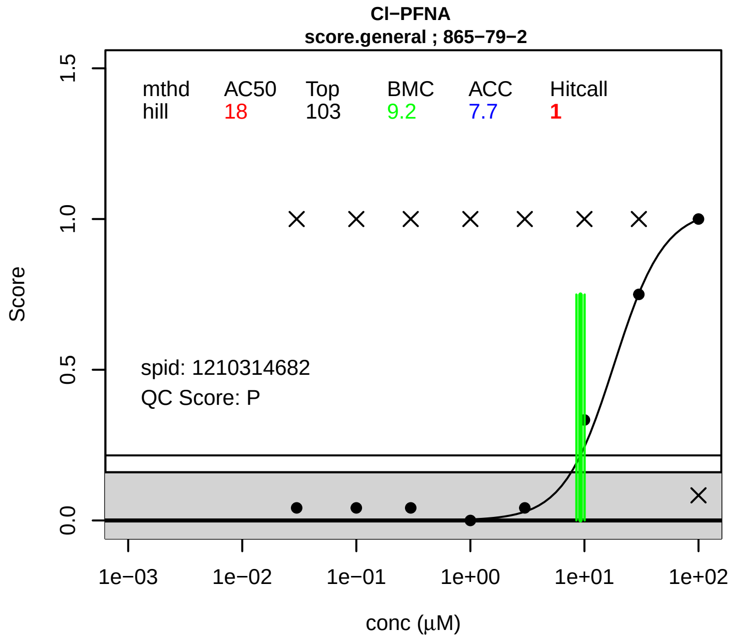
Cl−PFNA
score.general ; 865−79−2
mthd AC50 Top BMC ACC Hitcall
hill 18 103 9.2 7.7 1
spid: 1210314682
QC Score: P
Score
conc (µM)

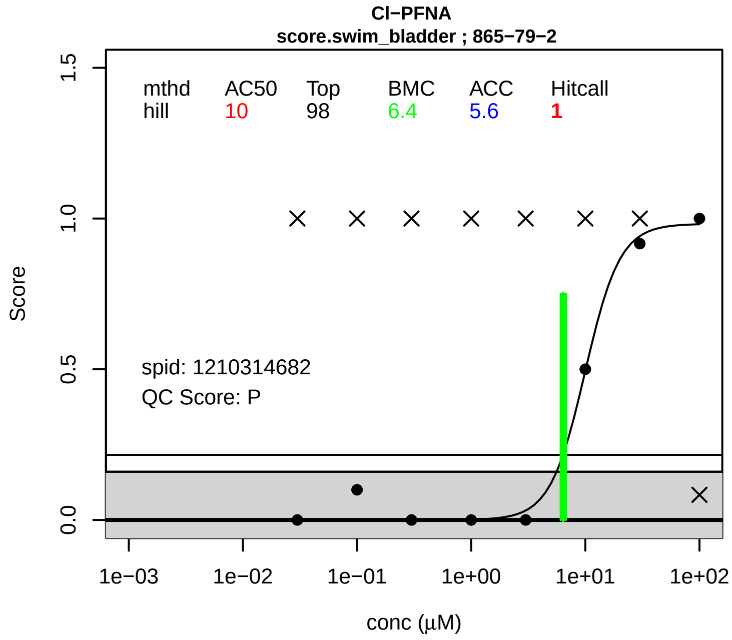
Cl−PFNA
score.swim_bladder ; 865−79−2
mthd AC50 Top BMC ACC Hitcall
hill 10 98 6.4 5.6 1
spid: 1210314682
QC Score: P
Score
conc (µM)

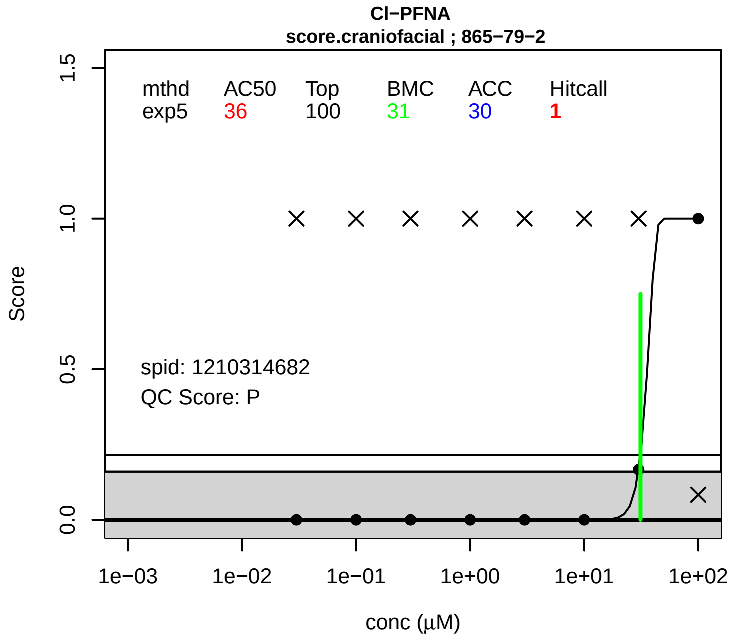
Cl−PFNA
score.craniofacial ; 865−79−2
mthd AC50 Top BMC ACC Hitcall
exp5 36 100 31 30 1
spid: 1210314682
QC Score: P
Score
conc (µM)

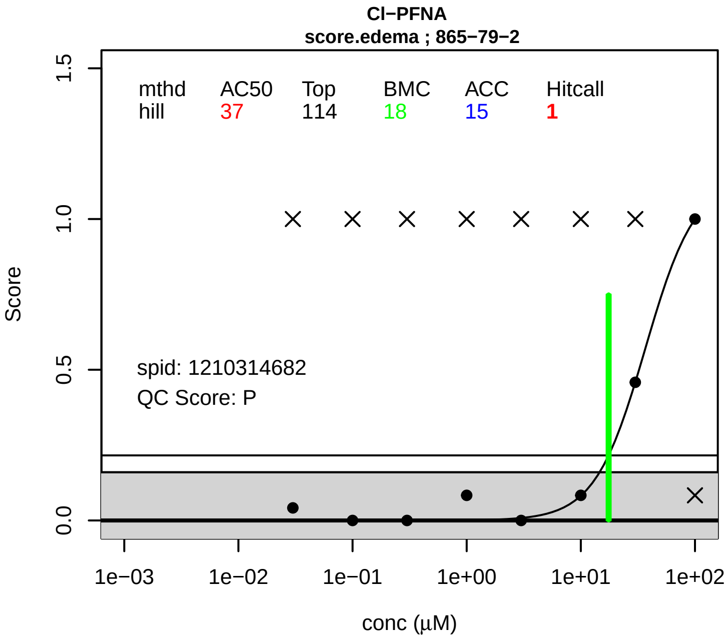
Cl−PFNA
score.edema ; 865−79−2
mthd hill
AC50 37
Top 114
BMC 18
ACC 15
Hitcall 1
spid: 1210314682
QC Score: P
Score
conc (µM)

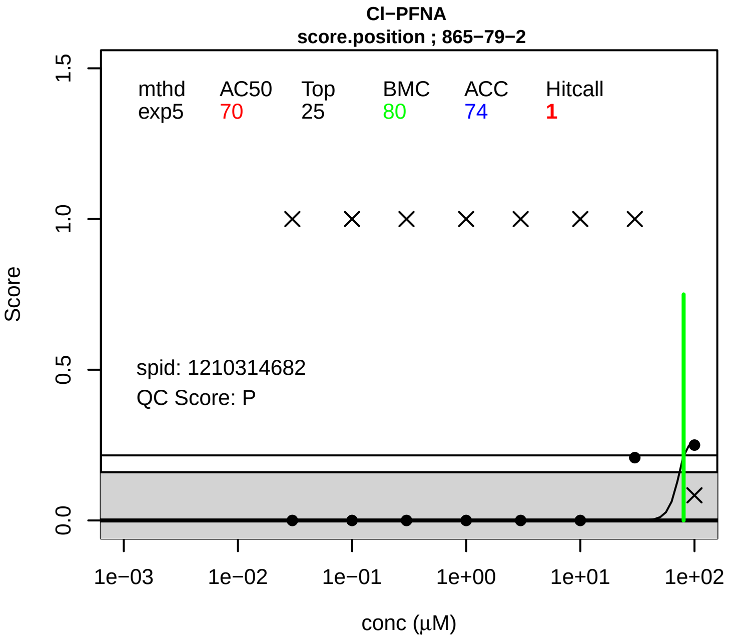
Cl−PFNA
score.position ; 865−79−2
mthd exp5
AC50 70
Top 25
BMC 80
ACC 74
Hitcall 1
spid: 1210314682
QC Score: P
Score
conc (µM)

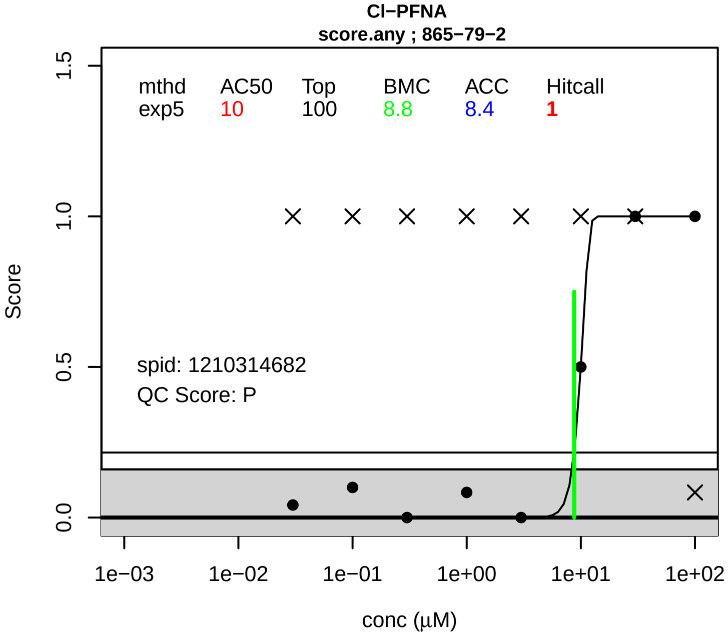
Cl−PFNA
score.any ; 865−79−2
mthd exp5
AC50 10
Top 100
BMC 8.8
ACC 8.4
Hitcall 1
spid: 1210314682
QC Score: P
Score
conc (µM)

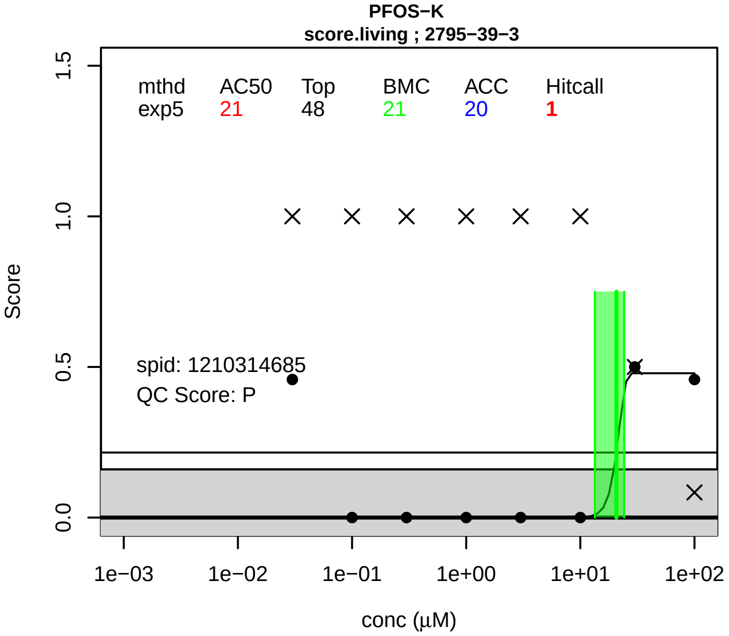
PFOS−K
score.living ; 2795−39−3
mthd exp5
AC50 21
Top 48
BMC 21
ACC 20
Hitcall 1
spid: 1210314685
QC Score: P
Score
conc (µM)

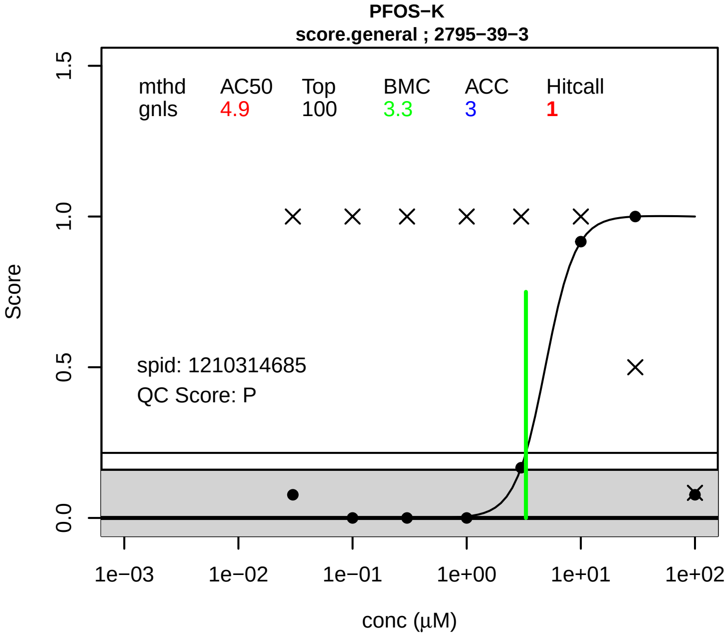
PFOS−K
score.general ; 2795−39−3
mthd gnls
AC50 4.9
Top 100
BMC 3.3
ACC 3
Hitcall 1
spid: 1210314685
QC Score: P
Score
conc (µM)

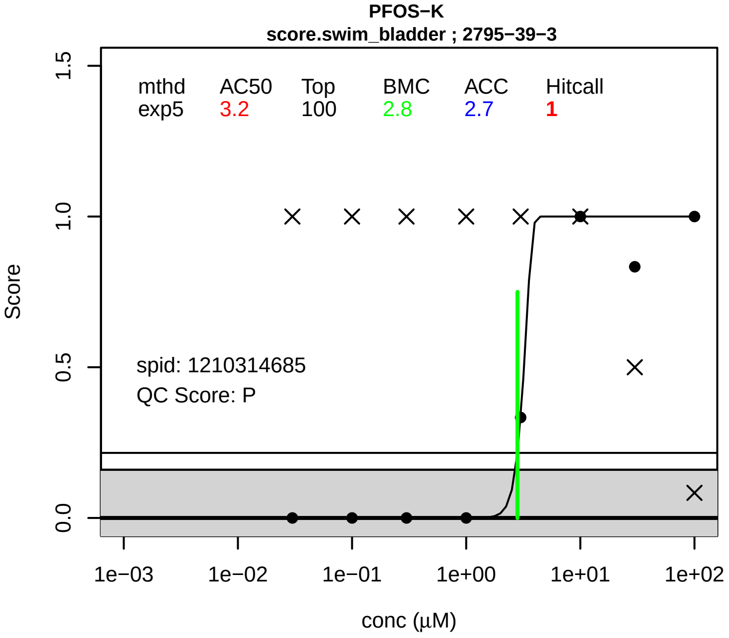
PFOS−K
score.swim_bladder ; 2795−39−3
mthd exp5
AC50 3.2
Top 100
BMC 2.8
ACC 2.7
Hitcall 1
spid: 1210314685
QC Score: P
Score
conc (µM)

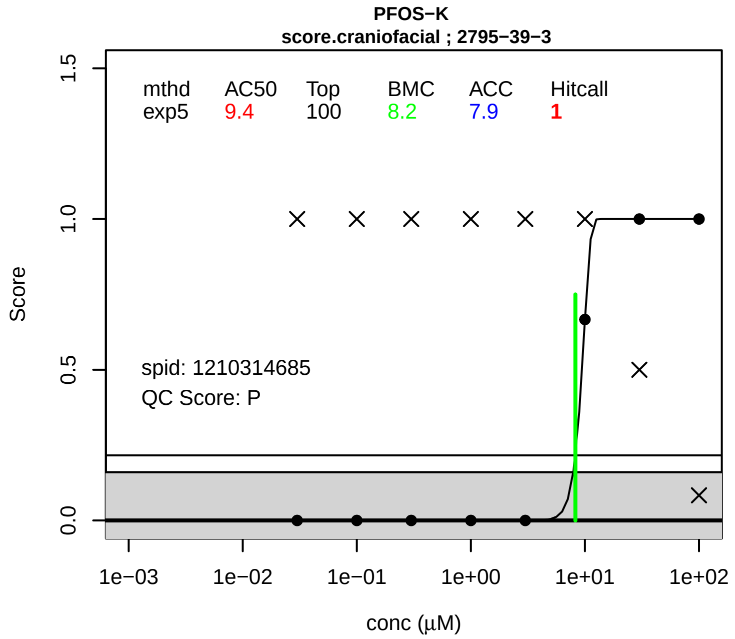
PFOS−K
score.craniofacial ; 2795−39−3
mthd exp5
AC50 9.4
Top 100
BMC 8.2
ACC 7.9
Hitcall 1
spid: 1210314685
QC Score: P
Score
0.0 0.5 1.0 1.5
1e−03 1e−02 1e−01 1e+00 1e+01 1e+02
conc (µM)

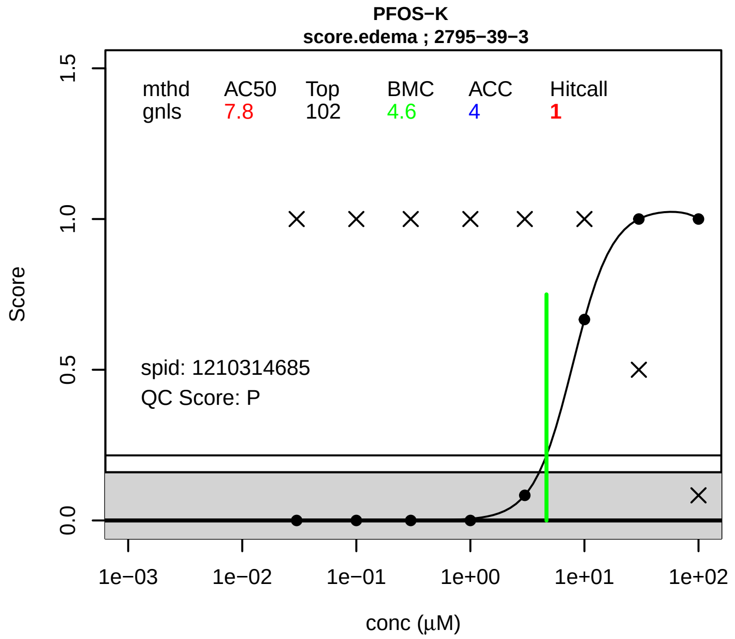
PFOS−K
score.edema ; 2795−39−3
mthd gnls
AC50 7.8
Top 102
BMC 4.6
ACC 4
Hitcall 1
spid: 1210314685
QC Score: P
Score
0.0 0.5 1.0 1.5
1e−03 1e−02 1e−01 1e+00 1e+01 1e+02
conc (µM)

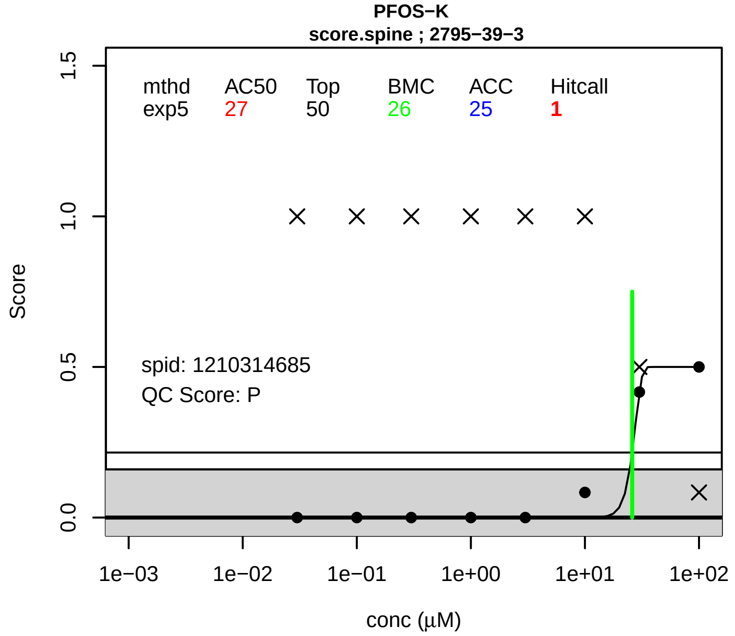
PFOS−K
score.spine ; 2795−39−3
mthd exp5
AC50 27
Top 50
BMC 26
ACC 25
Hitcall 1
spid: 1210314685
QC Score: P
Score
0.0 0.5 1.0 1.5
1e−03 1e−02 1e−01 1e+00 1e+01 1e+02
conc (µM)

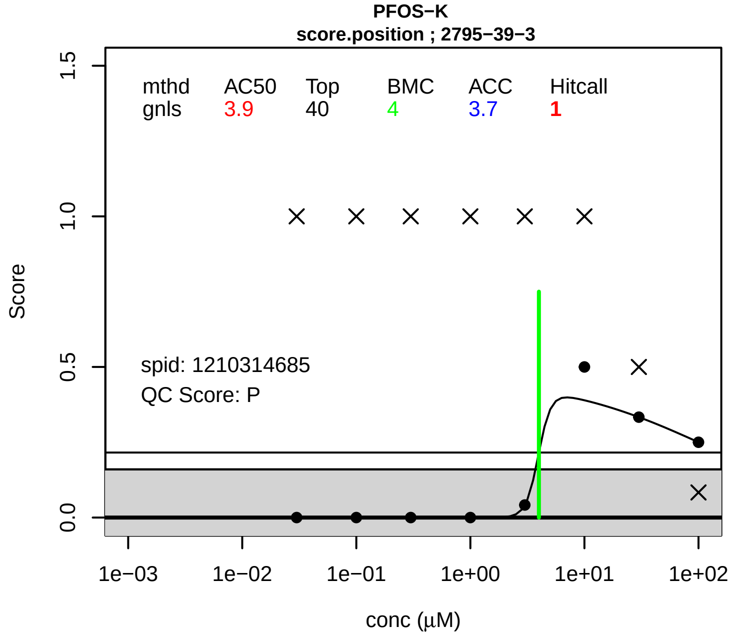
PFOS−K
score.position ; 2795−39−3
mthd gnls
AC50 3.9
Top 40
BMC 4
ACC 3.7
Hitcall 1
spid: 1210314685
QC Score: P
Score
0.0 0.5 1.0 1.5
1e−03 1e−02 1e−01 1e+00 1e+01 1e+02
conc (µM)

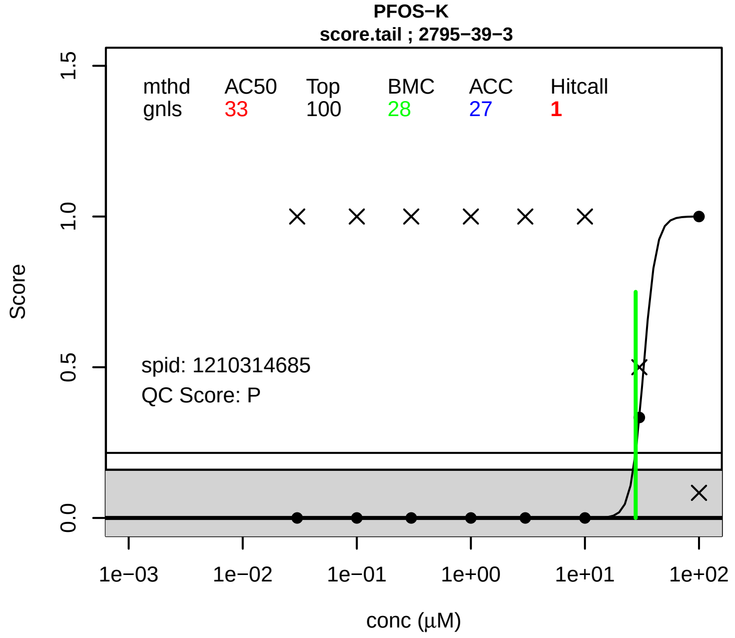
PFOS−K
score.tail ; 2795−39−3
mthd gnls
AC50 33
Top 100
BMC 28
ACC 27
Hitcall 1
spid: 1210314685
QC Score: P
Score
0.0 0.5 1.0 1.5
1e−03 1e−02 1e−01 1e+00 1e+01 1e+02
conc (µM)

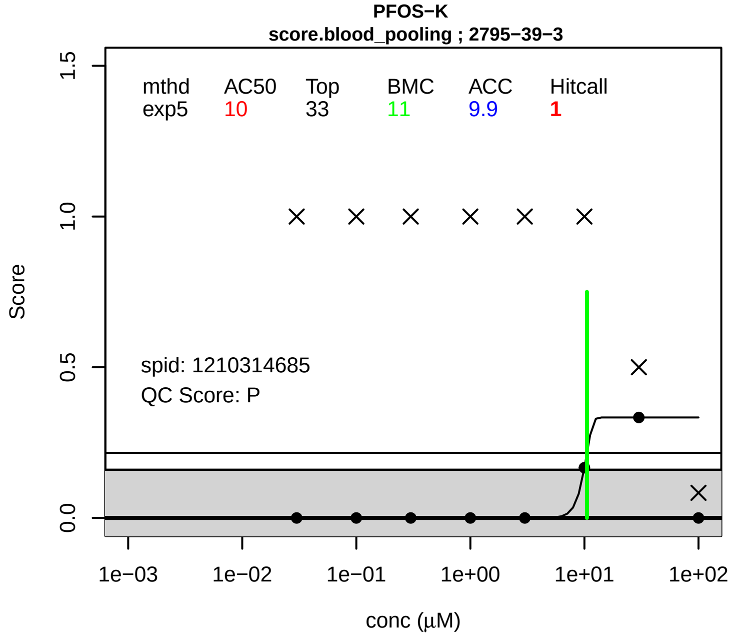
PFOS−K
score.blood_pooling ; 2795−39−3
mthd exp5
AC50 10
Top 33
BMC 11
ACC 9.9
Hitcall 1
spid: 1210314685
QC Score: P
Score
0.0 0.5 1.0 1.5
1e−03 1e−02 1e−01 1e+00 1e+01 1e+02
conc (µM)

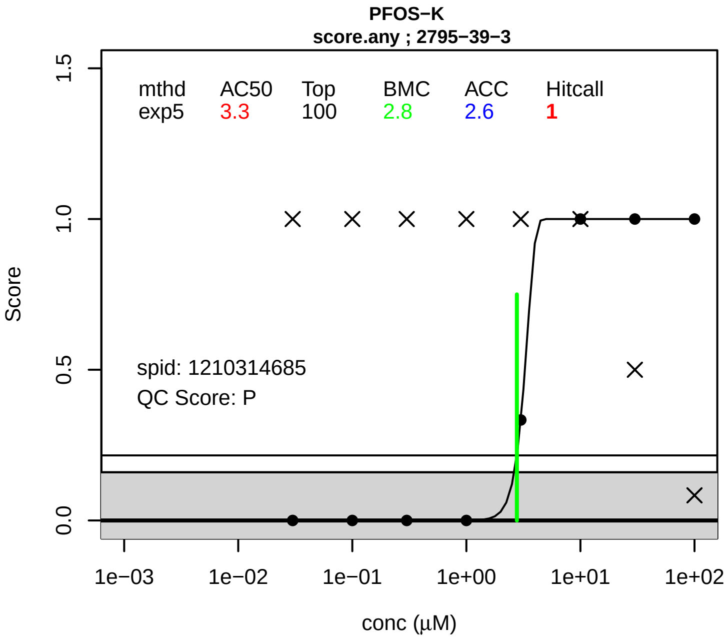
PFOS−K
score.any ; 2795−39−3
mthd AC50 Top BMC ACC Hitcall
exp5 3.3 100 2.8 2.6 1
spid: 1210314685
QC Score: P
Score
conc (µM)

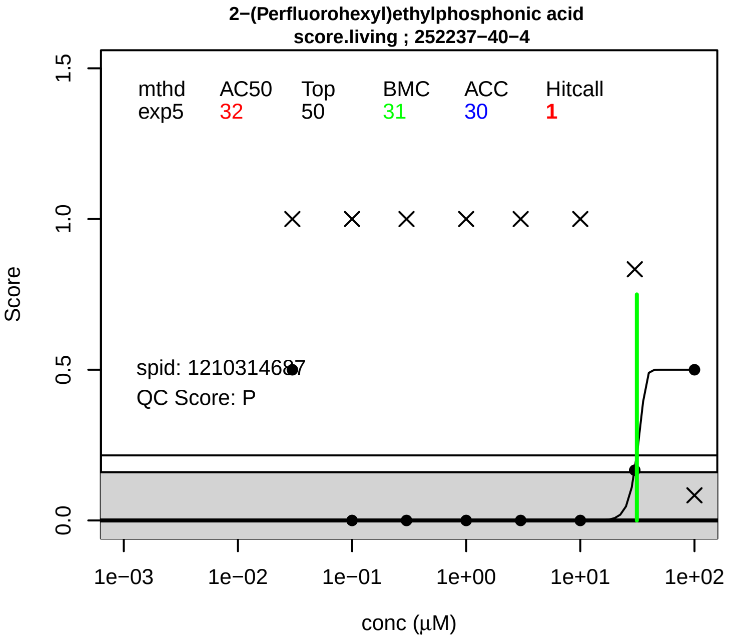
2−(Perfluorohexyl)ethylphosphonic acid
score.living ; 252237−40−4
mthd AC50 Top BMC ACC Hitcall
exp5 32 50 31 30 1
spid: 1210314687
QC Score: P
Score
conc (µM)

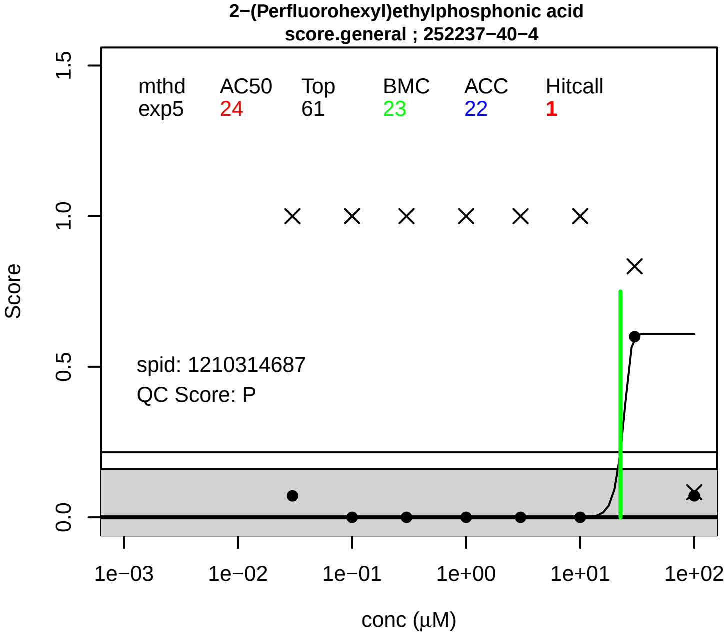
2−(Perfluorohexyl)ethylphosphonic acid
score.general ; 252237−40−4
mthd AC50 Top BMC ACC Hitcall
exp5 24 61 23 22 1
spid: 1210314687
QC Score: P
Score
conc (µM)

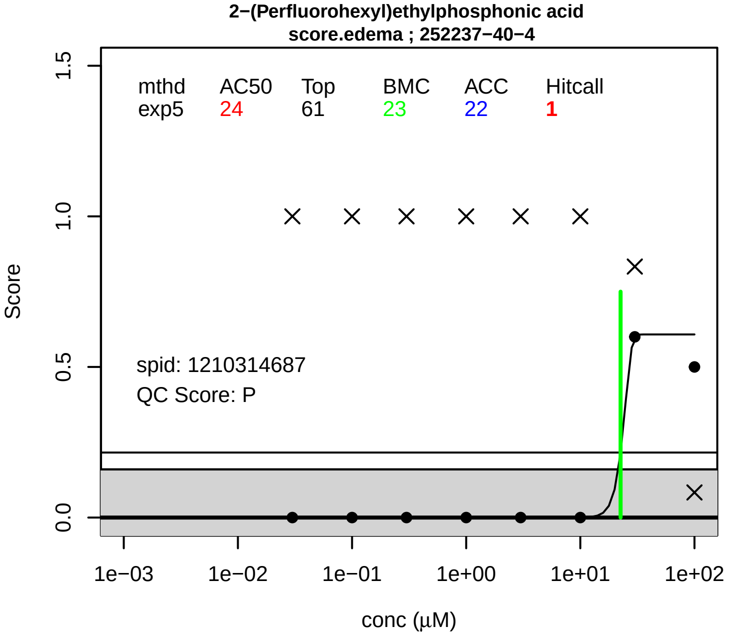
2−(Perfluorohexyl)ethylphosphonic acid
score.edema ; 252237−40−4
mthd AC50 Top BMC ACC Hitcall
exp5 24 61 23 22 1
spid: 1210314687
QC Score: P
Score
conc (µM)

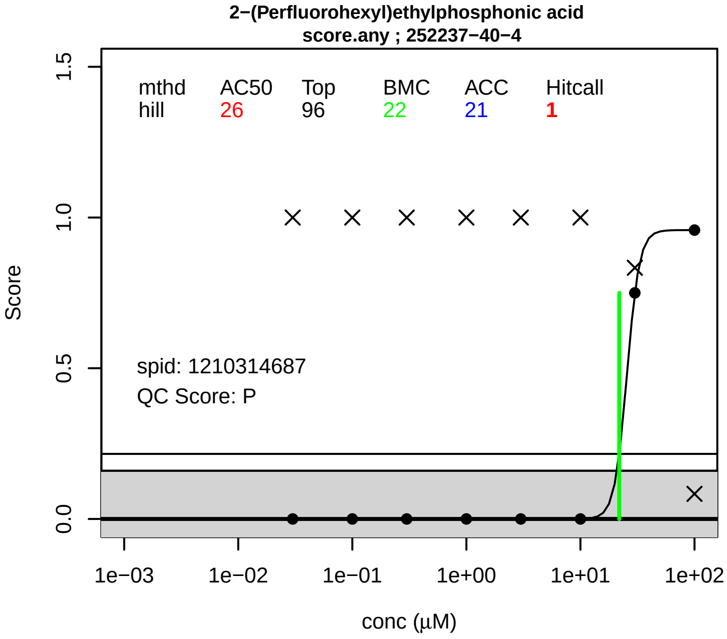
2−(Perfluorohexyl)ethylphosphonic acid
score.any ; 252237−40−4
mthd AC50 Top BMC ACC Hitcall
hill 26 96 22 21 1
spid: 1210314687
QC Score: P
Score
conc (µM)

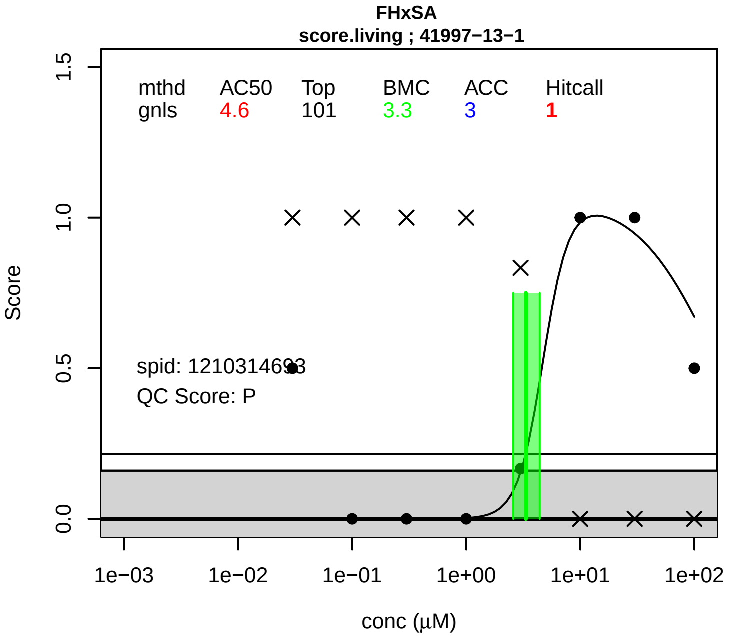
FHxSA
score.living ; 41997−13−1
mthd AC50 Top BMC ACC Hitcall
gnls 4.6 101 3.3 3 1
QC Score: P
Score
conc (µM)

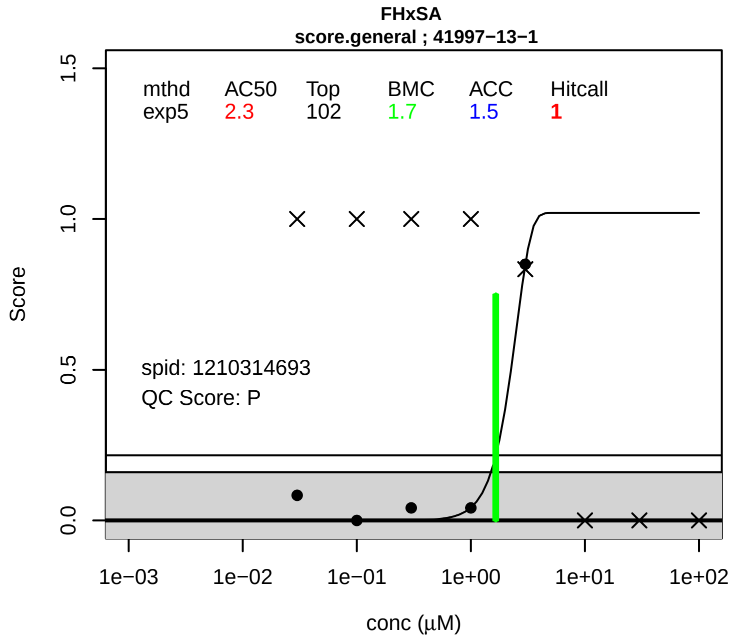
FHxSA
score.general ; 41997−13−1
mthd exp5
AC50 2.3
Top 102
BMC 1.7
ACC 1.5
Hitcall 1
spid: 1210314693
QC Score: P
Score
0.0 0.5 1.0 1.5
1e−03 1e−02 1e−01 1e+00 1e+01 1e+02
conc (µM)

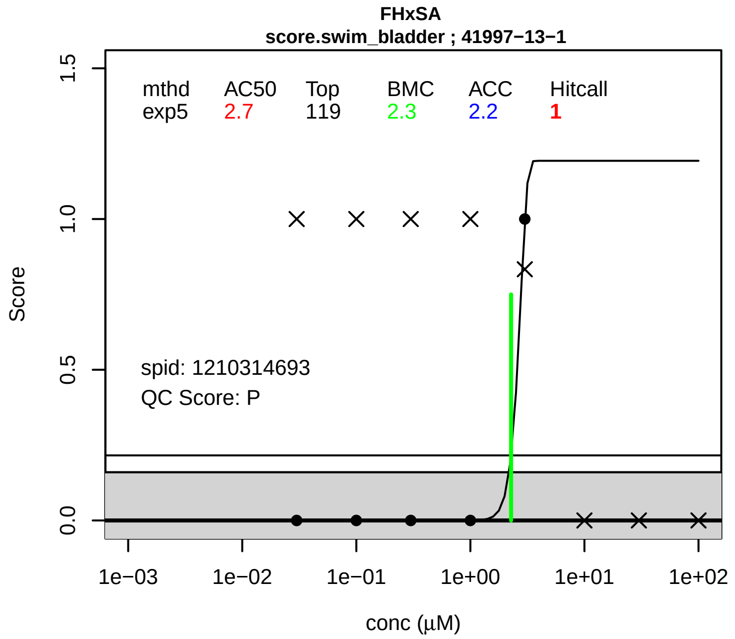
FHxSA
score.swim_bladder ; 41997−13−1
mthd exp5
AC50 2.7
Top 119
BMC 2.3
ACC 2.2
Hitcall 1
spid: 1210314693
QC Score: P
Score
0.0 0.5 1.0 1.5
1e−03 1e−02 1e−01 1e+00 1e+01 1e+02
conc (µM)

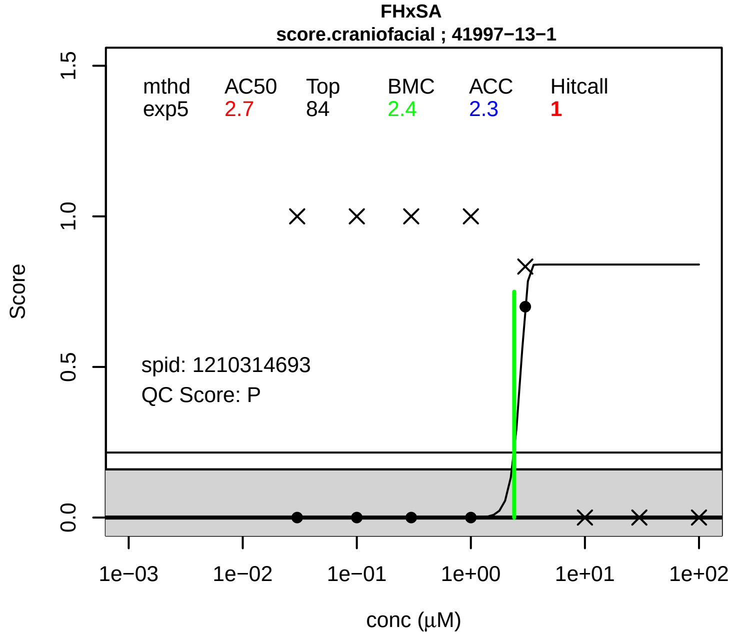
FHxSA
score.craniofacial ; 41997−13−1
mthd exp5
AC50 2.7
Top 84
BMC 2.4
ACC 2.3
Hitcall 1
spid: 1210314693
QC Score: P
Score
0.0 0.5 1.0 1.5
1e−03 1e−02 1e−01 1e+00 1e+01 1e+02
conc (µM)

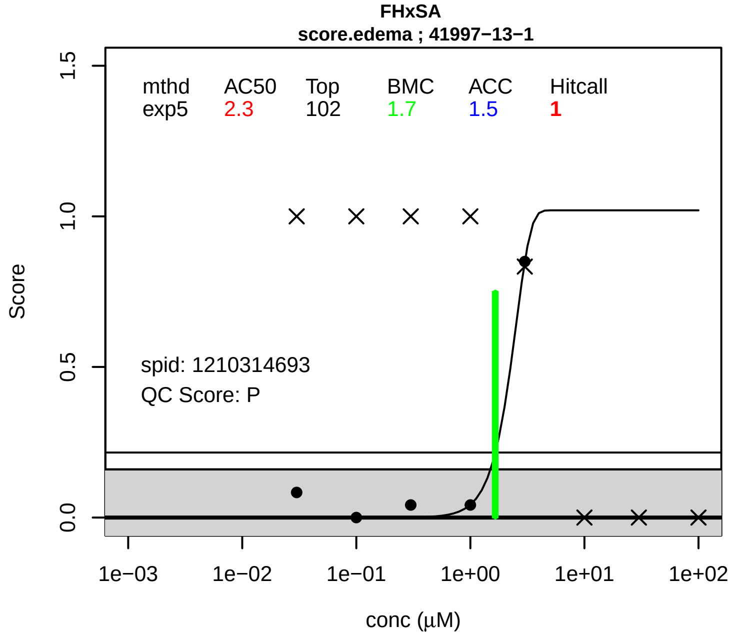
FHxSA
score.edema ; 41997−13−1
mthd exp5
AC50 2.3
Top 102
BMC 1.7
ACC 1.5
Hitcall 1
spid: 1210314693
QC Score: P
Score
0.0 0.5 1.0 1.5
1e−03 1e−02 1e−01 1e+00 1e+01 1e+02
conc (µM)

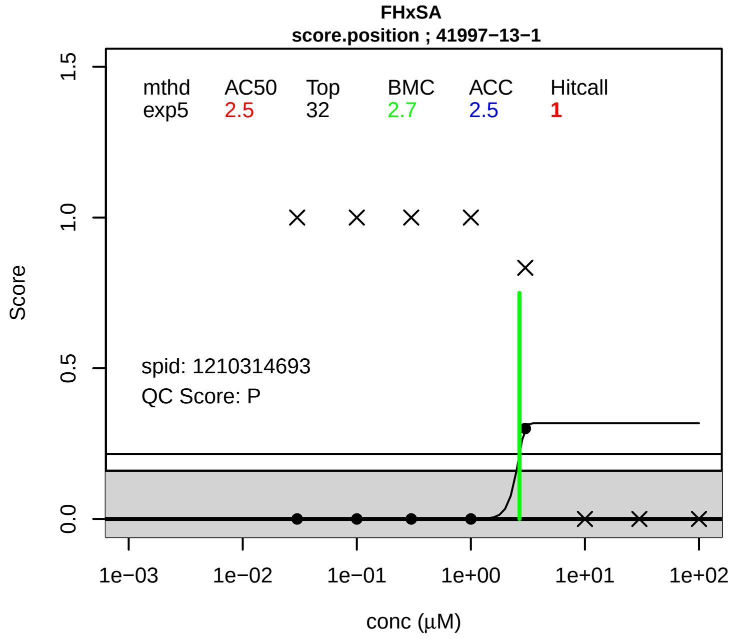
FHxSA
score.position ; 41997−13−1
mthd exp5
AC50 2.5
Top 32
BMC 2.7
ACC 2.5
Hitcall 1
spid: 1210314693
QC Score: P
Score
0.0 0.5 1.0 1.5
1e−03 1e−02 1e−01 1e+00 1e+01 1e+02
conc (µM)

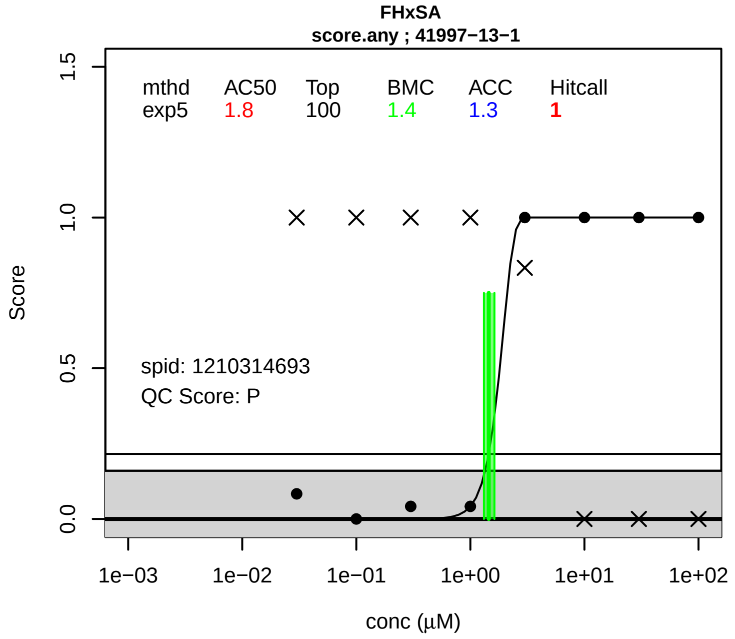
FHxSA
score.any ; 41997−13−1
mthd exp5
AC50 1.8
Top 100
BMC 1.4
ACC 1.3
Hitcall 1
spid: 1210314693
QC Score: P
Score
0.0 0.5 1.0 1.5
1e−03 1e−02 1e−01 1e+00 1e+01 1e+02
conc (µM)

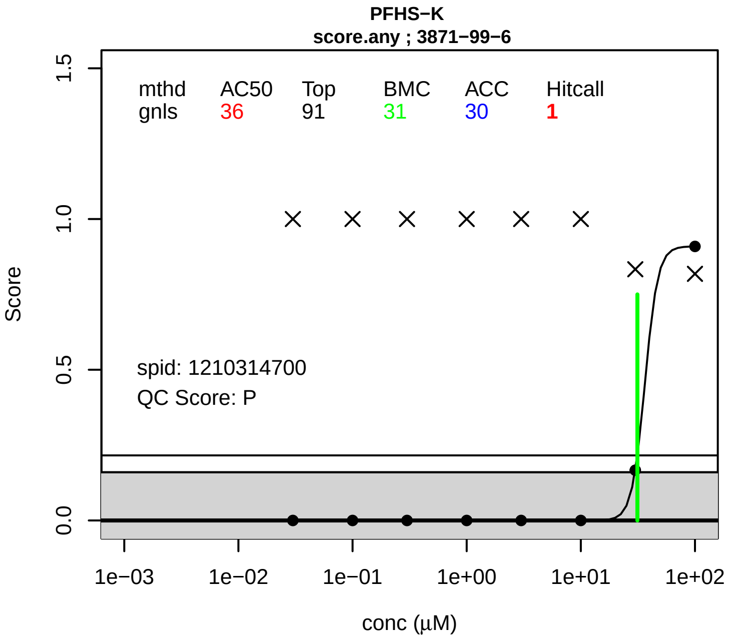
PFHS−K
score.any ; 3871−99−6
mthd AC50 Top BMC ACC Hitcall
gnls 36 91 31 30 1
spid: 1210314700
QC Score: P
Score
conc (µM)

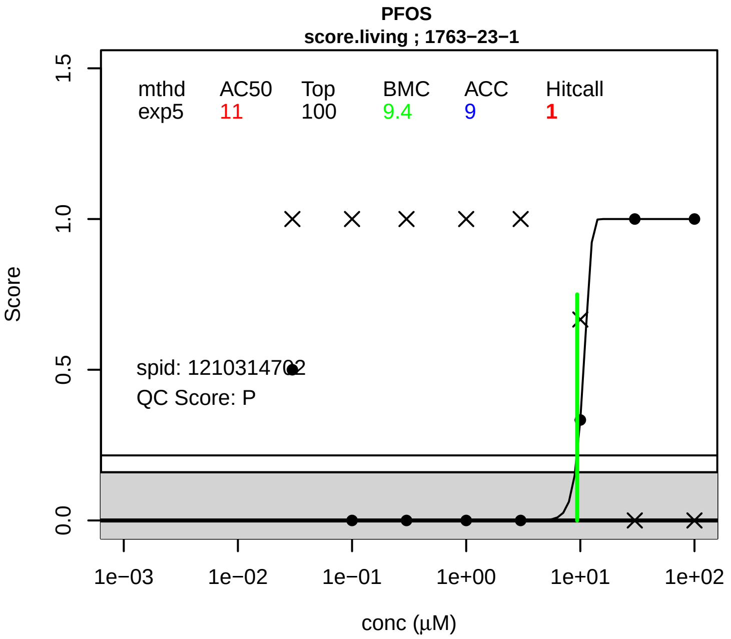
PFOS
score.living ; 1763−23−1
mthd AC50 Top BMC ACC Hitcall
exp5 11 100 9.4 9 1
spid: 1210314702
QC Score: P
Score
conc (µM)

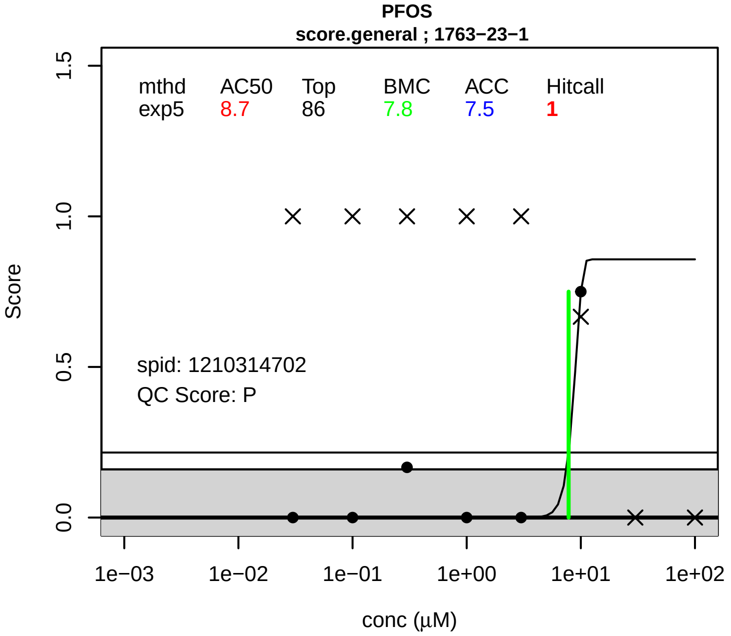
PFOS
score.general ; 1763−23−1
mthd AC50 Top BMC ACC Hitcall
exp5 8.7 86 7.8 7.5 1
spid: 1210314702
QC Score: P
Score
conc (µM)

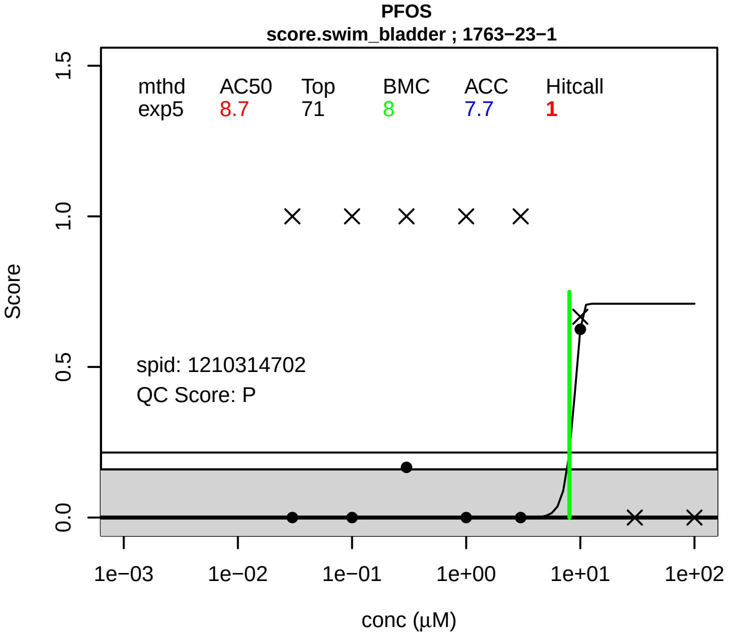
PFOS
score.swim_bladder ; 1763−23−1
mthd AC50 Top BMC ACC Hitcall
exp5 8.7 71 8 7.7 1
spid: 1210314702
QC Score: P
Score
conc (µM)

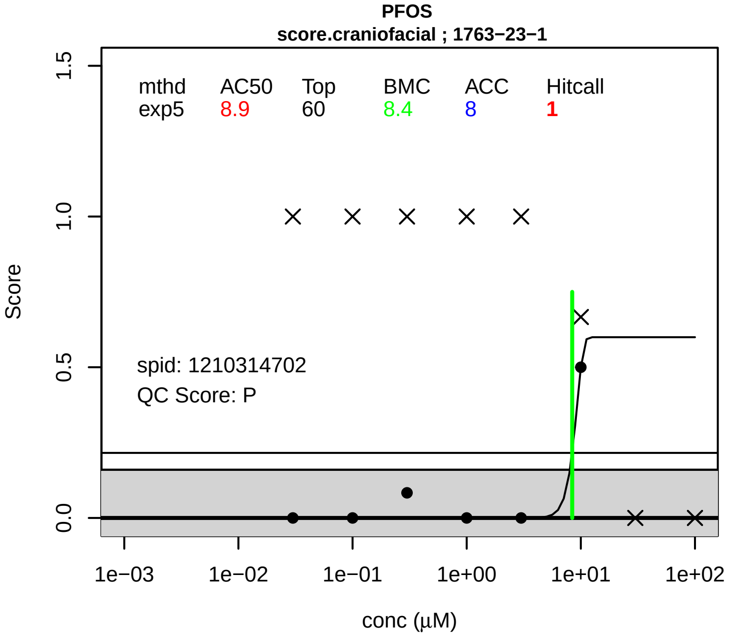
PFOS
score.craniofacial ; 1763−23−1
mthd AC50 Top BMC ACC Hitcall
exp5 8.9 60 8.4 8 1
spid: 1210314702
QC Score: P
Score
conc (µM)

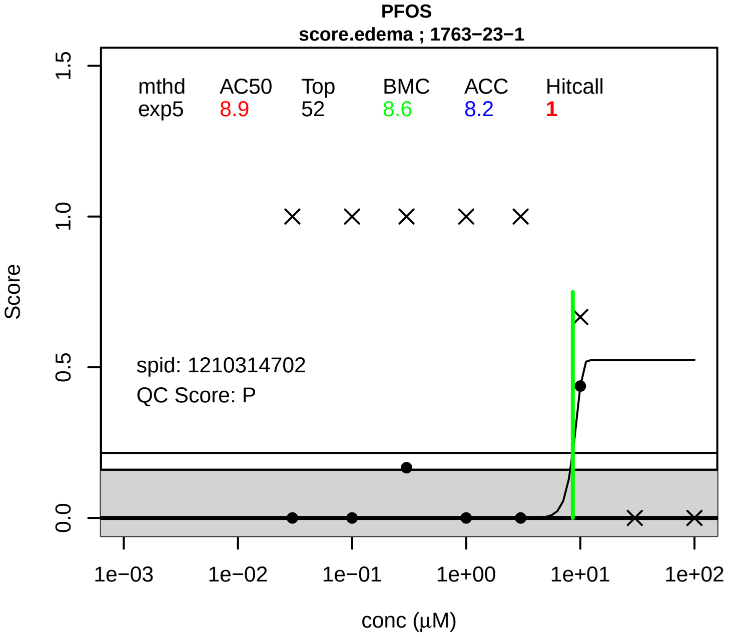
PFOS
score.edema ; 1763−23−1
mthd AC50 Top BMC ACC Hitcall
exp5 8.9 52 8.6 8.2 1
spid: 1210314702
QC Score: P
Score
conc (µM)

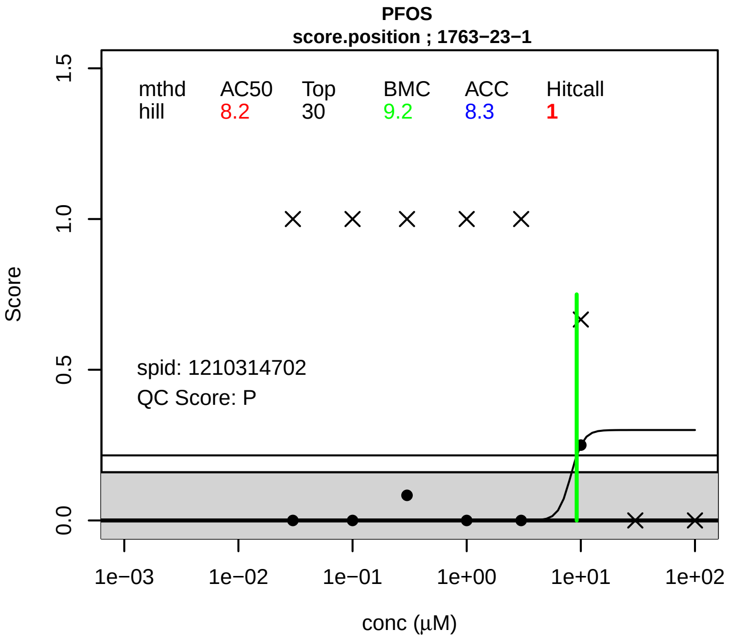
PFOS
score.position ; 1763−23−1
mthd AC50 Top BMC ACC Hitcall
hill 8.2 30 9.2 8.3 1
spid: 1210314702
QC Score: P
Score
conc (µM)

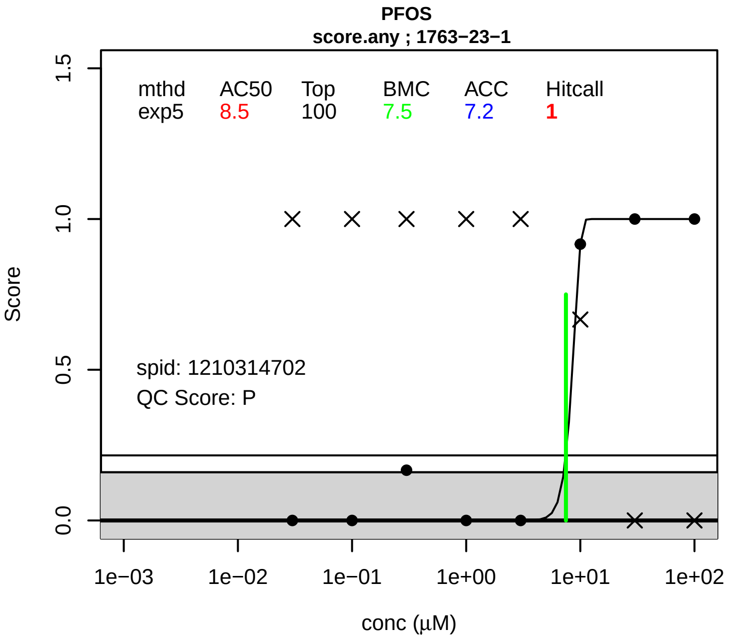
PFOS
score.any ; 1763−23−1
mthd AC50 Top BMC ACC Hitcall
exp5 8.5 100 7.5 7.2 1
spid: 1210314702
QC Score: P
Score
conc (µM)

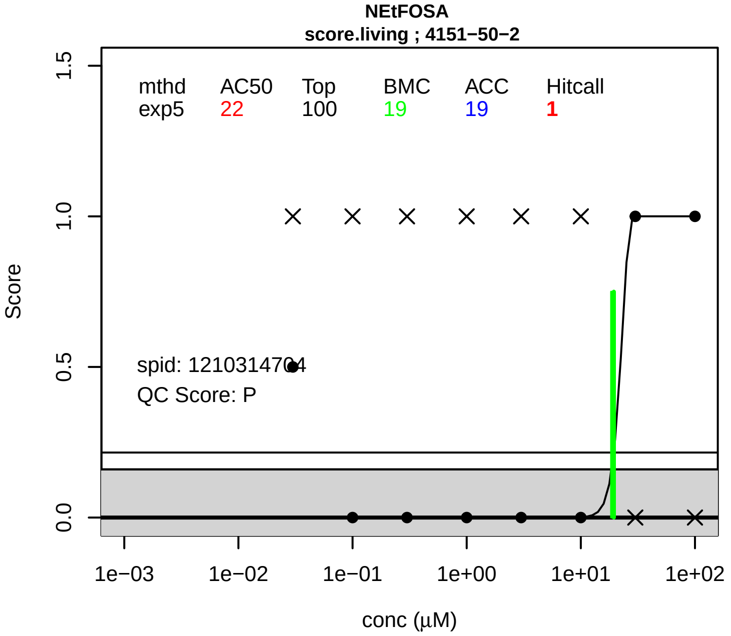
NEtFOSA
score.living ; 4151−50−2
mthd AC50 Top BMC ACC Hitcall
exp5 22 100 19 19 1
spid: 1210314704
QC Score: P
Score
conc (µM)

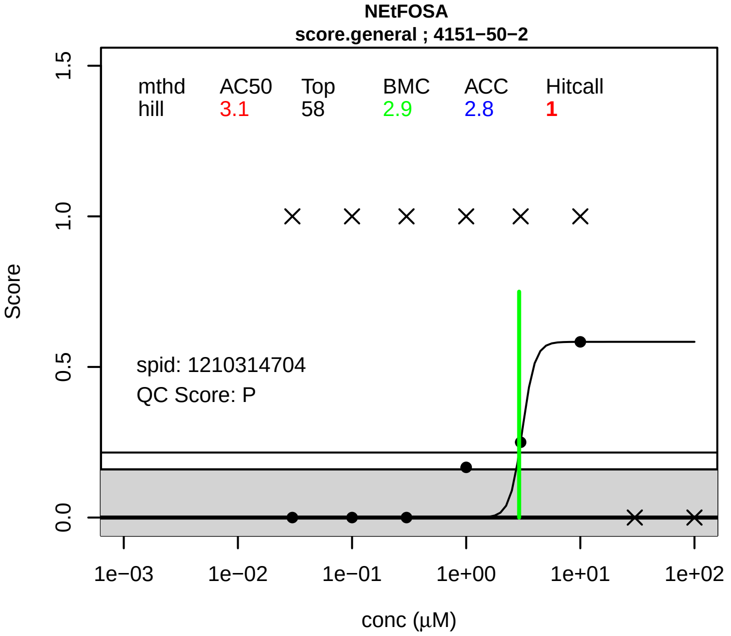
NEtFOSA
score.general ; 4151−50−2
mthd AC50 Top BMC ACC Hitcall
hill 3.1 58 2.9 2.8 1
spid: 1210314704
QC Score: P
Score
conc (µM)

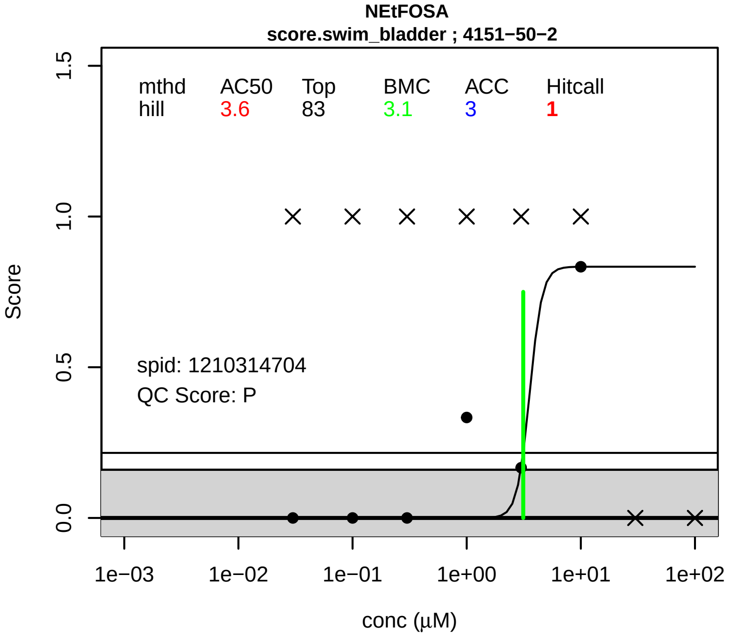
NEtFOSA
score.swim_bladder ; 4151−50−2
mthd AC50 Top BMC ACC Hitcall
hill 3.6 83 3.1 3 1
spid: 1210314704
QC Score: P
Score
conc (µM)

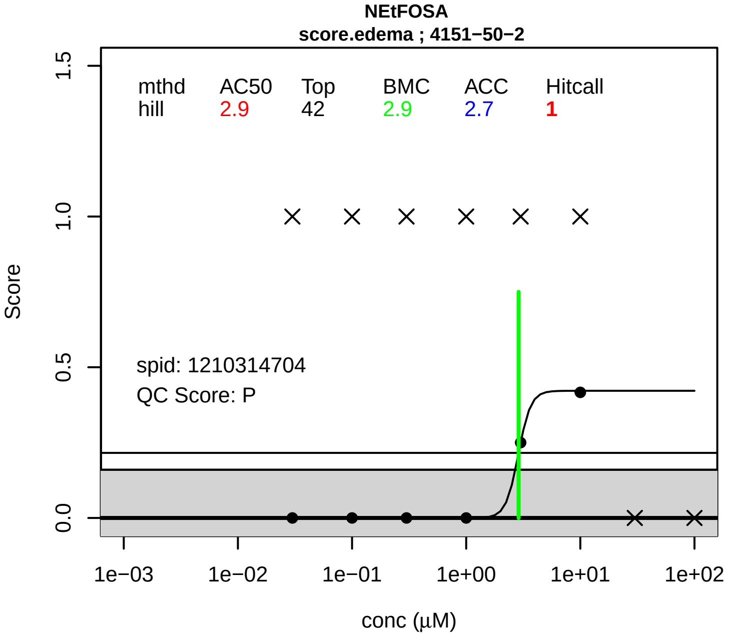
NEtFOSA
score.edema ; 4151−50−2
mthd AC50 Top BMC ACC Hitcall
hill 2.9 42 2.9 2.7 1
spid: 1210314704
QC Score: P
Score
conc (µM)

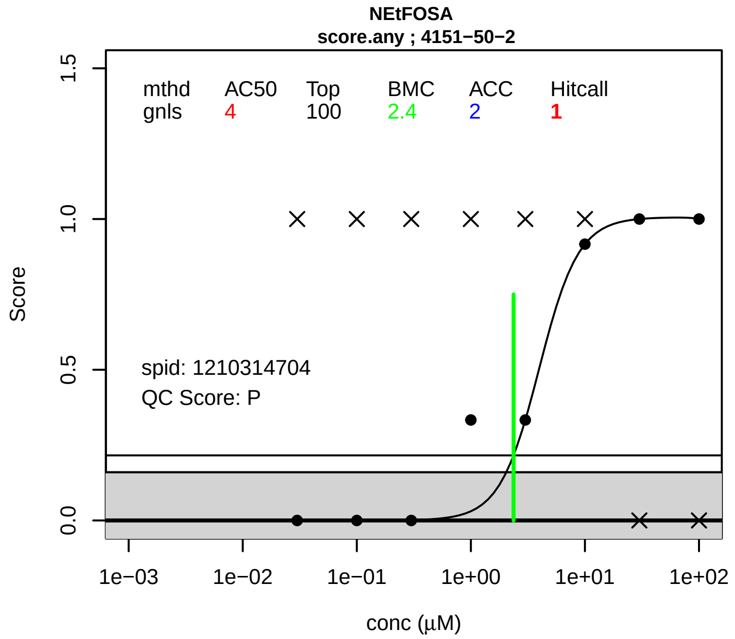
NEtFOSA
score.any ; 4151−50−2
mthd AC50 Top BMC ACC Hitcall
gnls 4 100 2.4 2 1
spid: 1210314704
QC Score: P
Score
conc (µM)
0.0 0.5 1.0 1.5
1e−03 1e−02 1e−01 1e+00 1e+01 1e+02

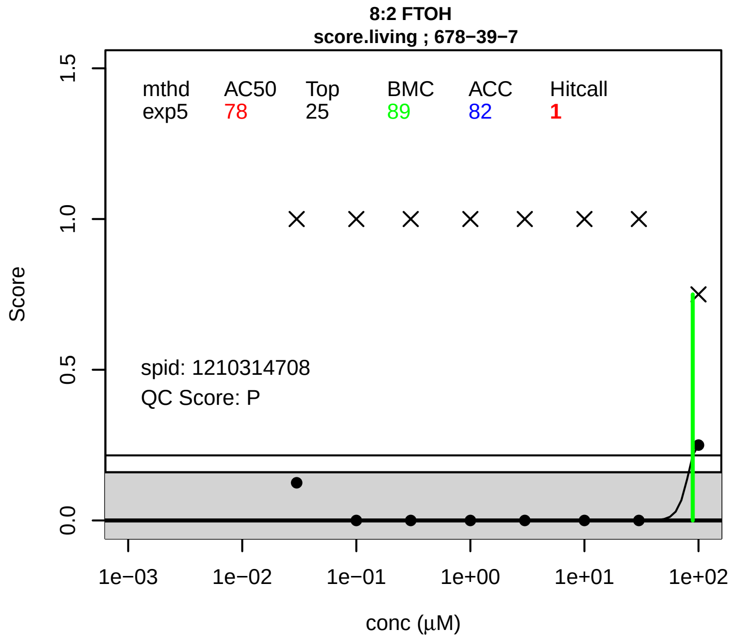
8:2 FTOH
score.living ; 678−39−7
mthd AC50 Top BMC ACC Hitcall
exp5 78 25 89 82 1
spid: 1210314708
QC Score: P
Score
conc (µM)
0.0 0.5 1.0 1.5
1e−03 1e−02 1e−01 1e+00 1e+01 1e+02

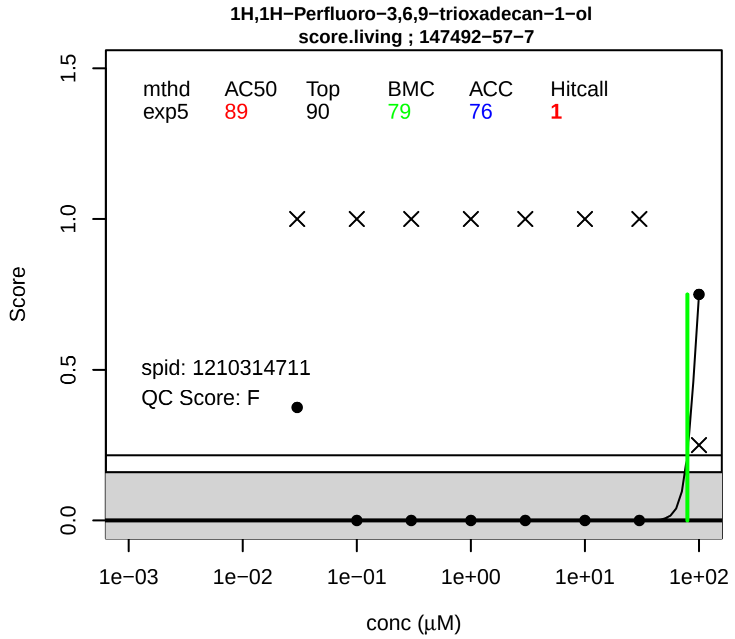
1H,1H−Perfluoro−3,6,9−trioxadecan−1−ol
score.living ; 147492−57−7
mthd AC50 Top BMC ACC Hitcall
exp5 89 90 79 76 1
spid: 1210314711
QC Score: F
Score
conc (µM)
0.0 0.5 1.0 1.5
1e−03 1e−02 1e−01 1e+00 1e+01 1e+02

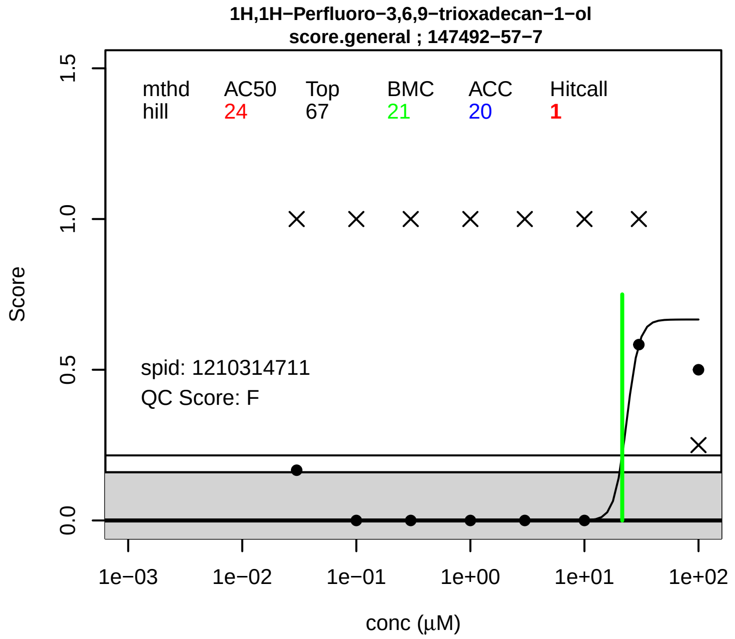
1H,1H−Perfluoro−3,6,9−trioxadecan−1−ol
score.general ; 147492−57−7
mthd AC50 Top BMC ACC Hitcall
hill 24 67 21 20 1
spid: 1210314711
QC Score: F
Score
conc (µM)
0.0 0.5 1.0 1.5
1e−03 1e−02 1e−01 1e+00 1e+01 1e+02

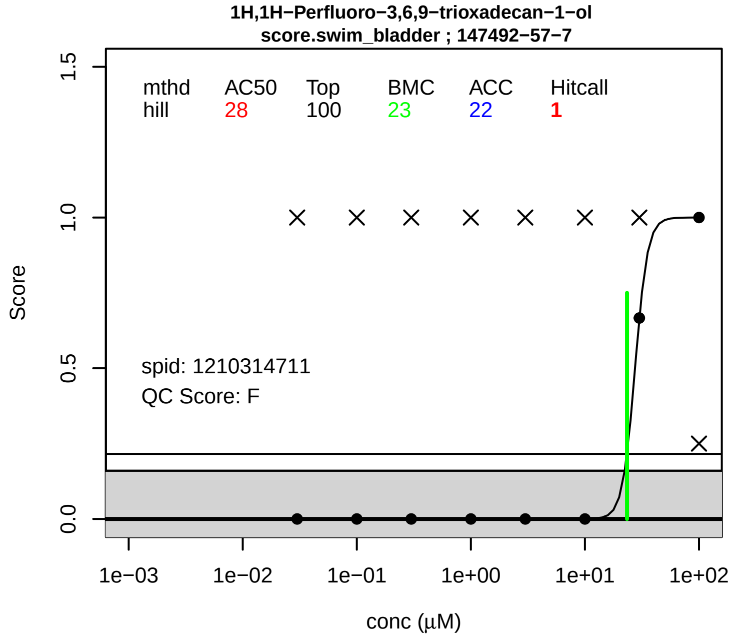
1H,1H−Perfluoro−3,6,9−trioxadecan−1−ol
score.swim_bladder ; 147492−57−7
mthd AC50 Top BMC ACC Hitcall
hill 28 100 23 22 1
spid: 1210314711
QC Score: F
Score
conc (µM)
0.0 0.5 1.0 1.5
1e−03 1e−02 1e−01 1e+00 1e+01 1e+02

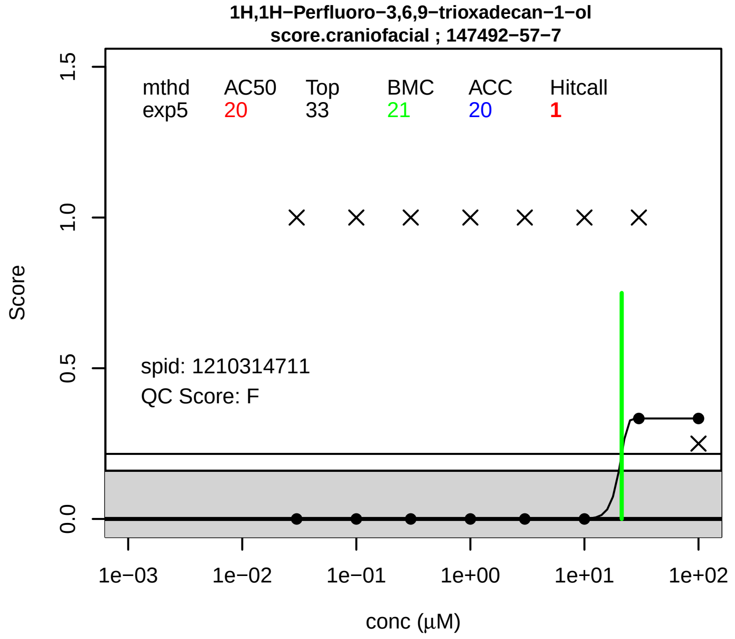
1H,1H−Perfluoro−3,6,9−trioxadecan−1−ol
score.craniofacial ; 147492−57−7
mthd AC50 Top BMC ACC Hitcall
exp5 20 33 21 20 1
spid: 1210314711
QC Score: F
Score
conc (µM)
0.0 0.5 1.0 1.5
1e−03 1e−02 1e−01 1e+00 1e+01 1e+02

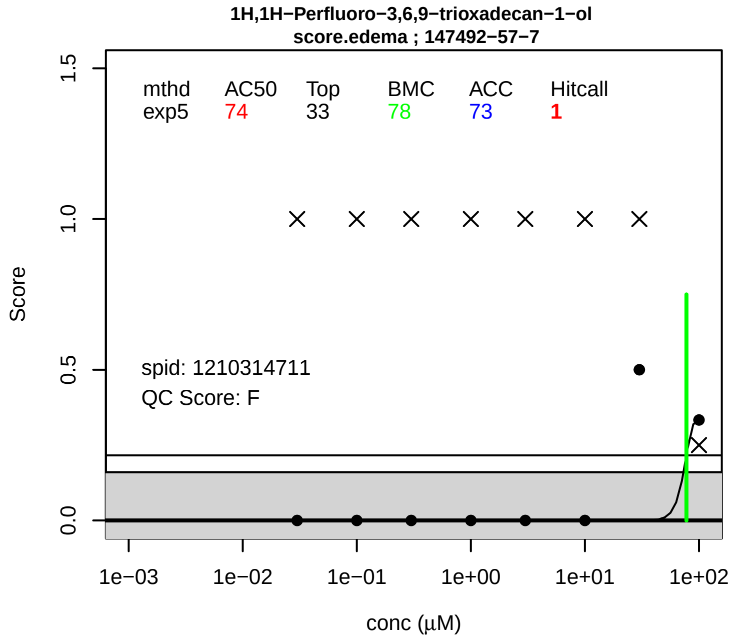
1H,1H−Perfluoro−3,6,9−trioxadecan−1−ol
score.edema ; 147492−57−7
mthd AC50 Top BMC ACC Hitcall
exp5 74 33 78 73 1
spid: 1210314711
QC Score: F
Score
conc (µM)

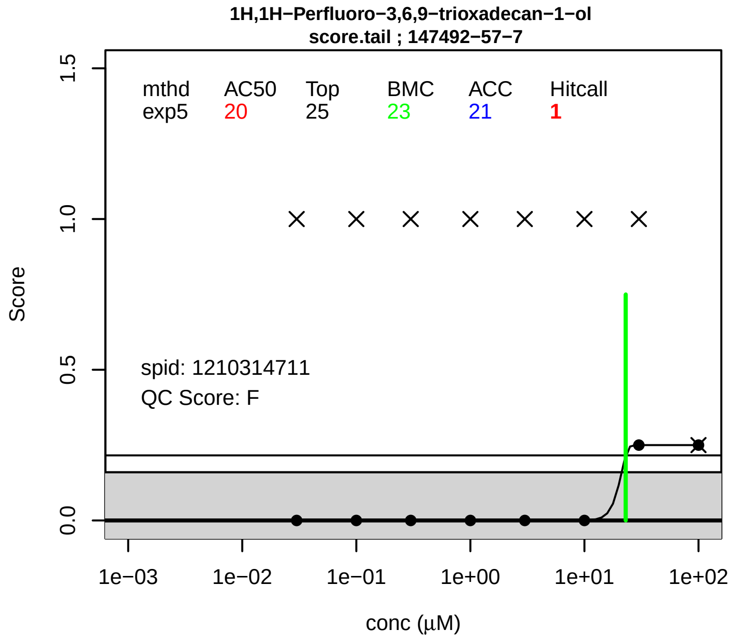
1H,1H−Perfluoro−3,6,9−trioxadecan−1−ol
score.tail ; 147492−57−7
mthd AC50 Top BMC ACC Hitcall
exp5 20 25 23 21 1
spid: 1210314711
QC Score: F
Score
conc (µM)

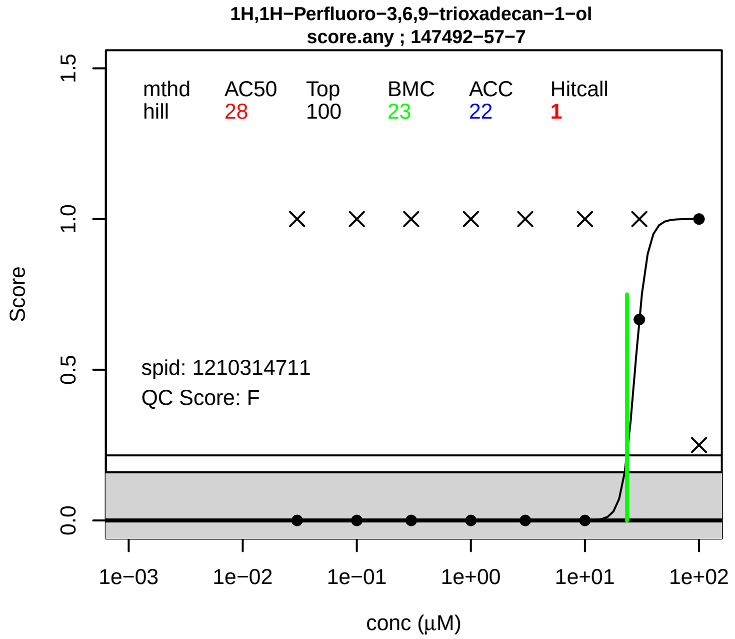
1H,1H−Perfluoro−3,6,9−trioxadecan−1−ol
score.any ; 147492−57−7
mthd AC50 Top BMC ACC Hitcall
hill 28 100 23 22 1
spid: 1210314711
QC Score: F
Score
conc (µM)

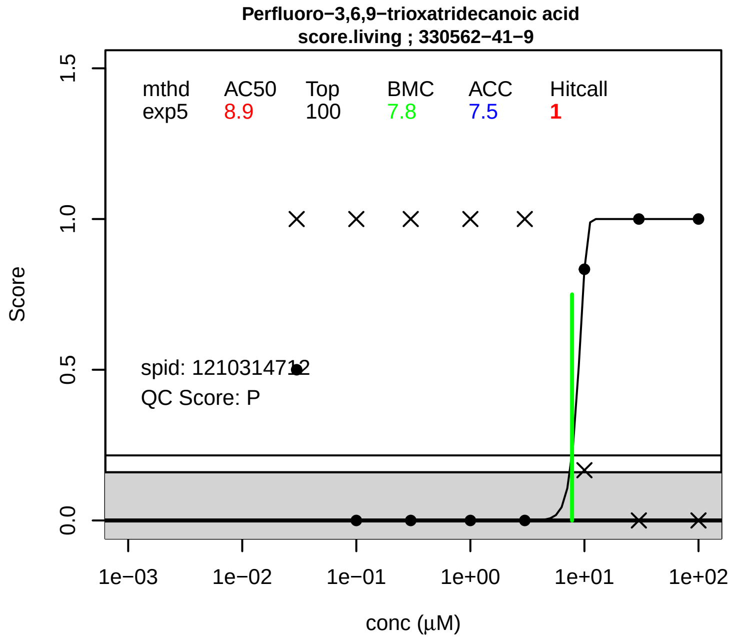
Perfluoro−3,6,9−trioxatridecanoic acid
score.living ; 330562−41−9
mthd AC50 Top BMC ACC Hitcall
exp5 8.9 100 7.8 7.5 1
spid: 1210314712
QC Score: P
Score
conc (µM)

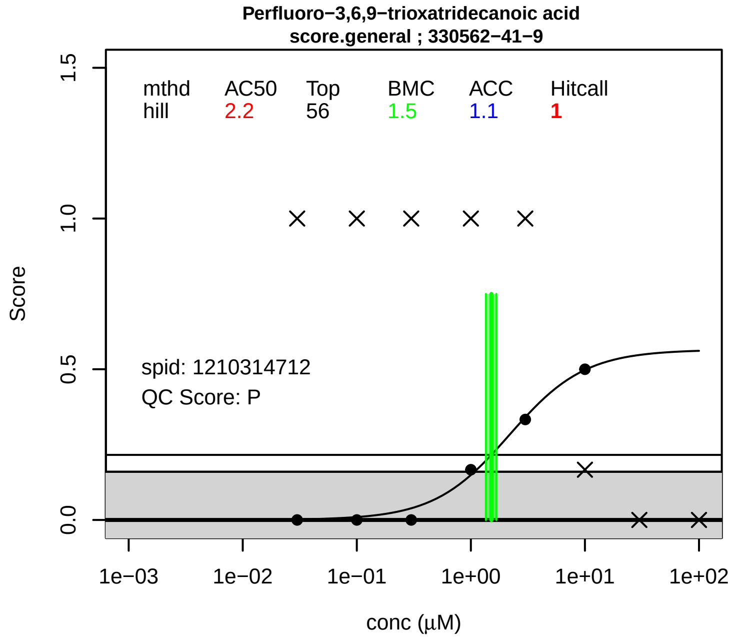
Perfluoro−3,6,9−trioxatridecanoic acid
score.general ; 330562−41−9
mthd AC50 Top BMC ACC Hitcall
hill 2.2 56 1.5 1.1 1
spid: 1210314712
QC Score: P
Score
conc (µM)

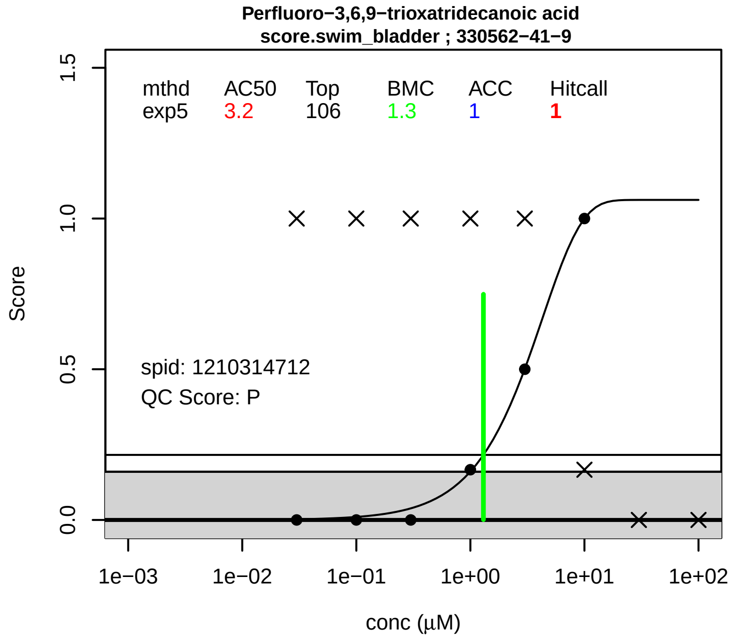
Perfluoro−3,6,9−trioxatridecanoic acid
score.swim_bladder ; 330562−41−9
mthd AC50 Top BMC ACC Hitcall
exp5 3.2 106 1.3 1 1
spid: 1210314712
QC Score: P
Score
conc (µM)

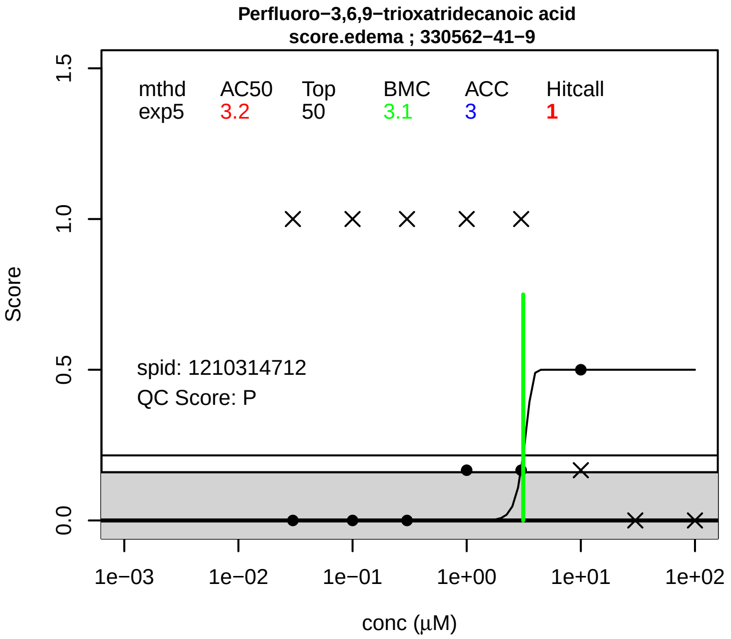
Perfluoro−3,6,9−trioxatridecanoic acid
score.edema ; 330562−41−9
mthd AC50 Top BMC ACC Hitcall
exp5 3.2 50 3.1 3 1
spid: 1210314712
QC Score: P
Score
conc (µM)

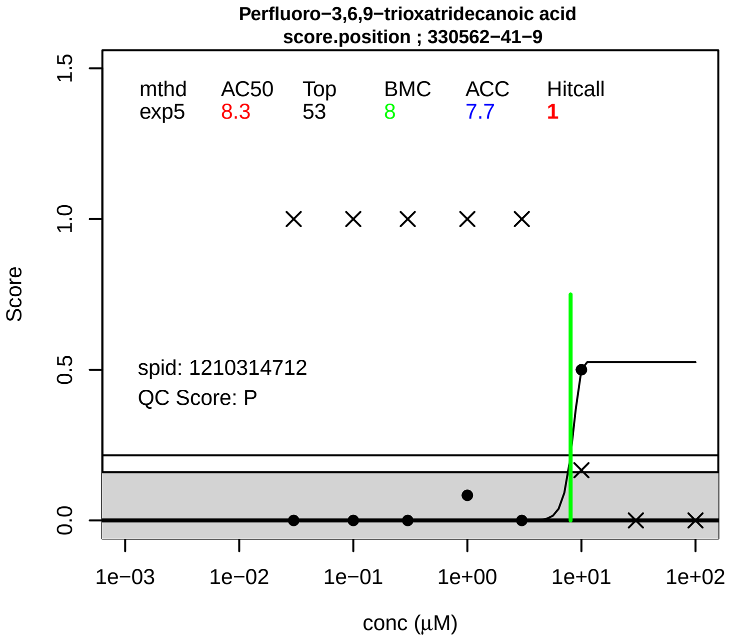
Perfluoro−3,6,9−trioxatridecanoic acid
score.position ; 330562−41−9
mthd AC50 Top BMC ACC Hitcall
exp5 8.3 53 8 7.7 1
spid: 1210314712
QC Score: P
Score
conc (µM)

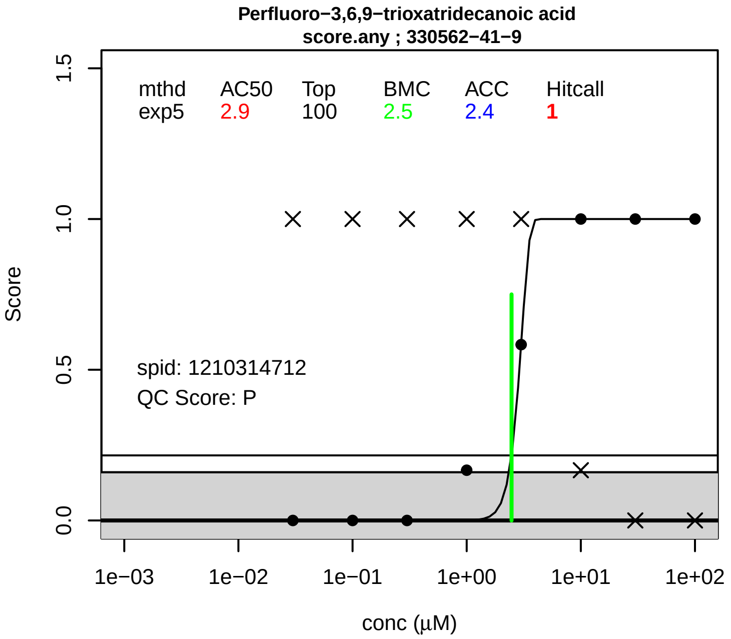
Perfluoro−3,6,9−trioxatridecanoic acid
score.any ; 330562−41−9
mthd AC50 Top BMC ACC Hitcall
exp5 2.9 100 2.5 2.4 1
spid: 1210314712
QC Score: P
Score
conc (µM)

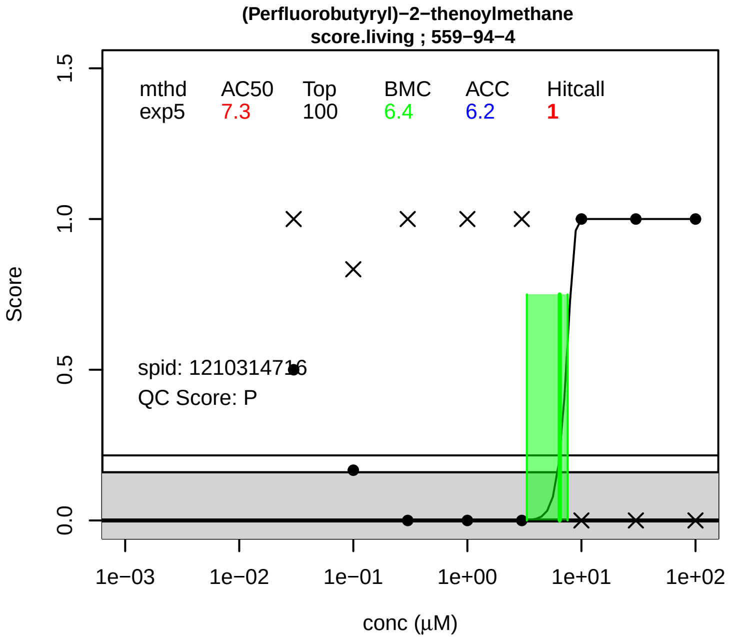
(Perfluorobutyryl)−2−thenoylmethane
score.living ; 559−94−4
mthd AC50 Top BMC ACC Hitcall
exp5 7.3 100 6.4 6.2 1
spid: 1210314716
QC Score: P
Score
conc (µM)

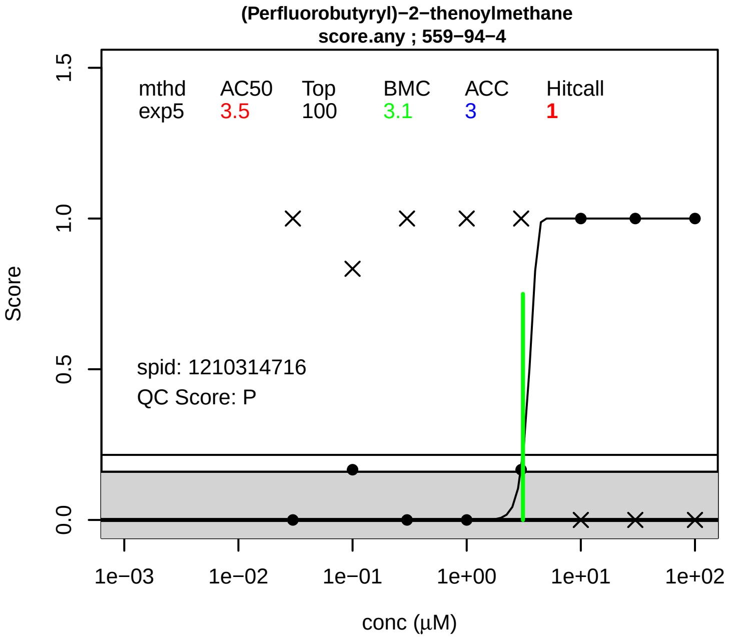
(Perfluorobutyryl)−2−thenoylmethane
score.any ; 559−94−4
mthd AC50 Top BMC ACC Hitcall
exp5 3.5 100 3.1 3 1
spid: 1210314716
QC Score: P
Score
conc (µM)

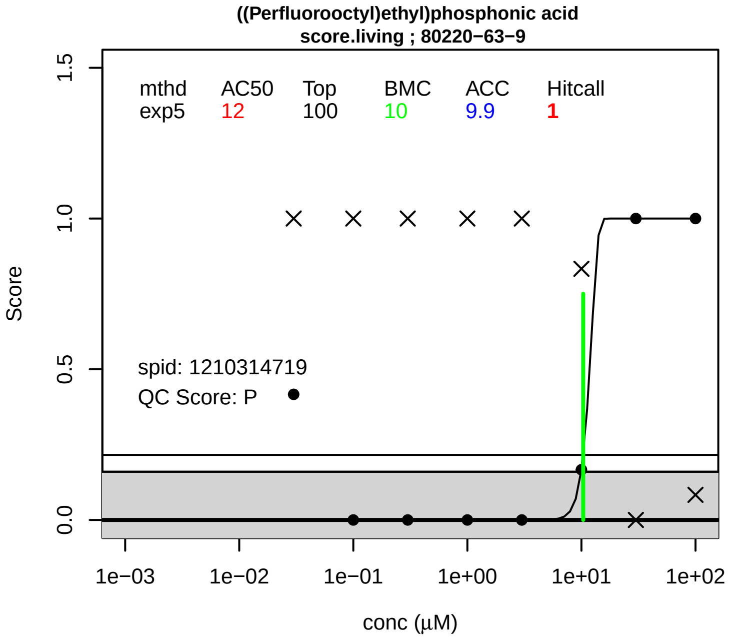
((Perfluorooctyl)ethyl)phosphonic acid
score.living ; 80220−63−9
mthd AC50 Top BMC ACC Hitcall
exp5 12 100 10 9.9 1
spid: 1210314719
QC Score: P
Score
conc (µM)

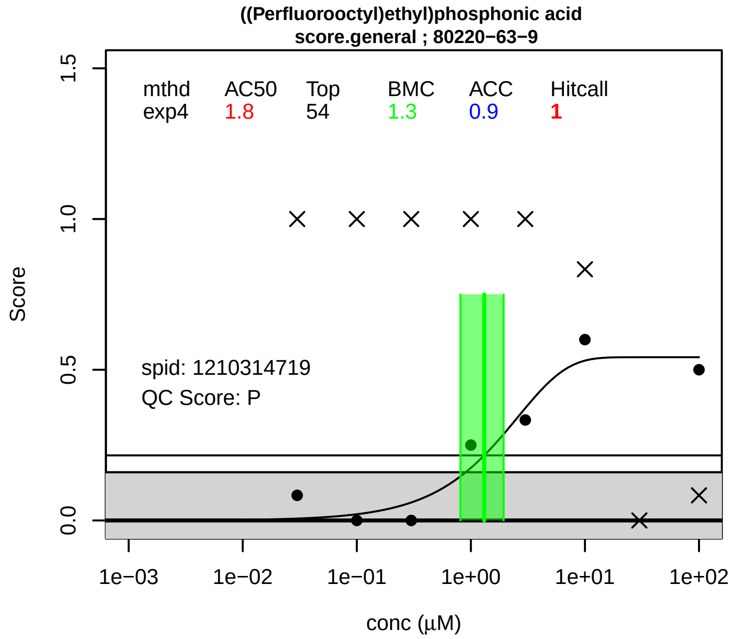
((Perfluorooctyl)ethyl)phosphonic acid
score.general ; 80220−63−9
mthd AC50 Top BMC ACC Hitcall
exp4 1.8 54 1.3 0.9 1
spid: 1210314719
QC Score: P
Score
conc (µM)
1e−03 1e−02 1e−01 1e+00 1e+01 1e+02
0.0 0.5 1.0 1.5

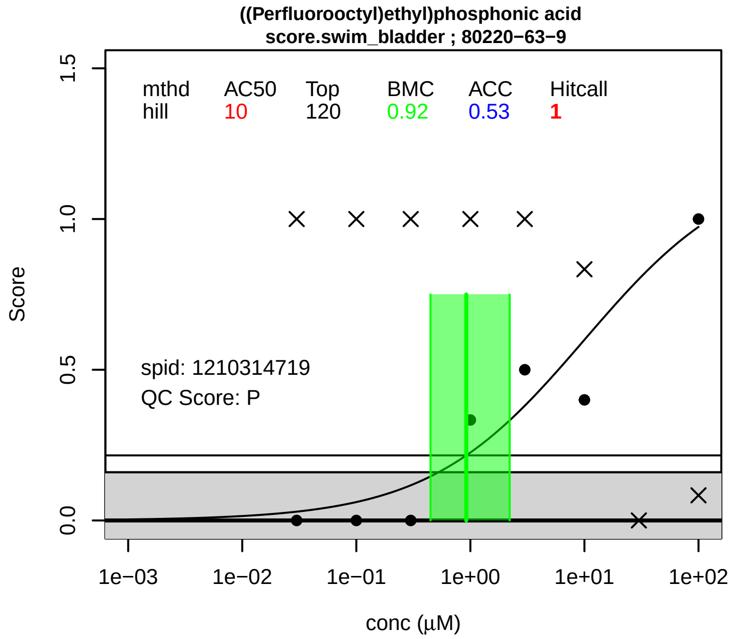
((Perfluorooctyl)ethyl)phosphonic acid
score.swim_bladder ; 80220−63−9
mthd AC50 Top BMC ACC Hitcall
hill 10 120 0.92 0.53 1
spid: 1210314719
QC Score: P
Score
conc (µM)
1e−03 1e−02 1e−01 1e+00 1e+01 1e+02
0.0 0.5 1.0 1.5

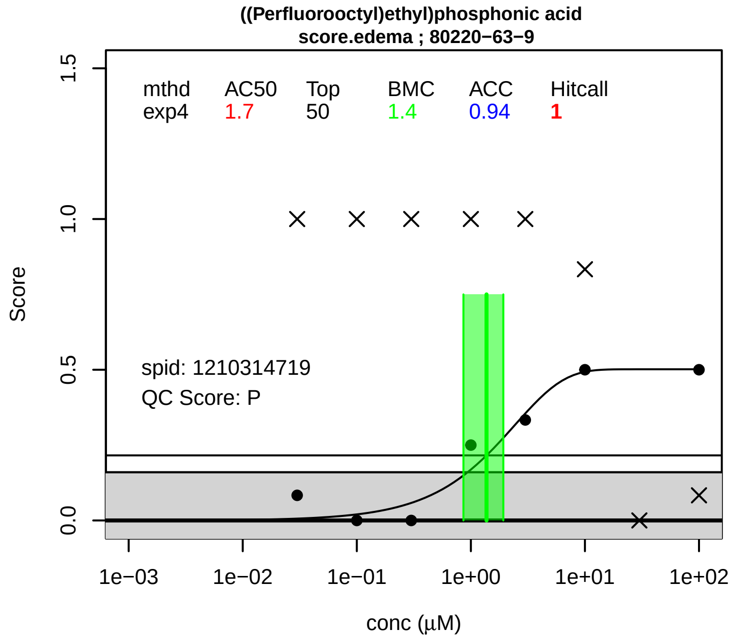
((Perfluorooctyl)ethyl)phosphonic acid
score.edema ; 80220−63−9
mthd AC50 Top BMC ACC Hitcall
exp4 1.7 50 1.4 0.94 1
spid: 1210314719
QC Score: P
Score
conc (µM)
1e−03 1e−02 1e−01 1e+00 1e+01 1e+02
0.0 0.5 1.0 1.5

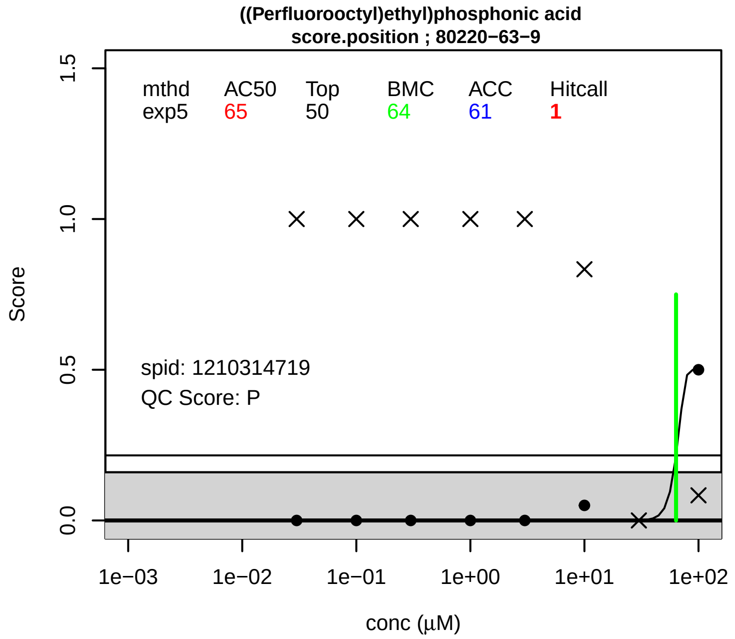
((Perfluorooctyl)ethyl)phosphonic acid
score.position ; 80220−63−9
mthd AC50 Top BMC ACC Hitcall
exp5 65 50 64 61 1
spid: 1210314719
QC Score: P
Score
conc (µM)
1e−03 1e−02 1e−01 1e+00 1e+01 1e+02
0.0 0.5 1.0 1.5

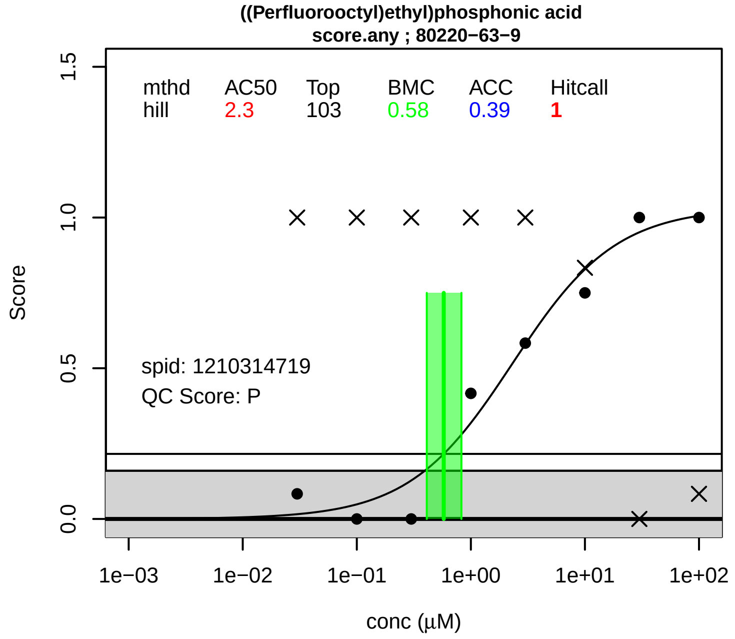
((Perfluorooctyl)ethyl)phosphonic acid
score.any ; 80220−63−9
mthd AC50 Top BMC ACC Hitcall
hill 2.3 103 0.58 0.39 1
spid: 1210314719
QC Score: P
Score
conc (µM)
1e−03 1e−02 1e−01 1e+00 1e+01 1e+02
0.0 0.5 1.0 1.5

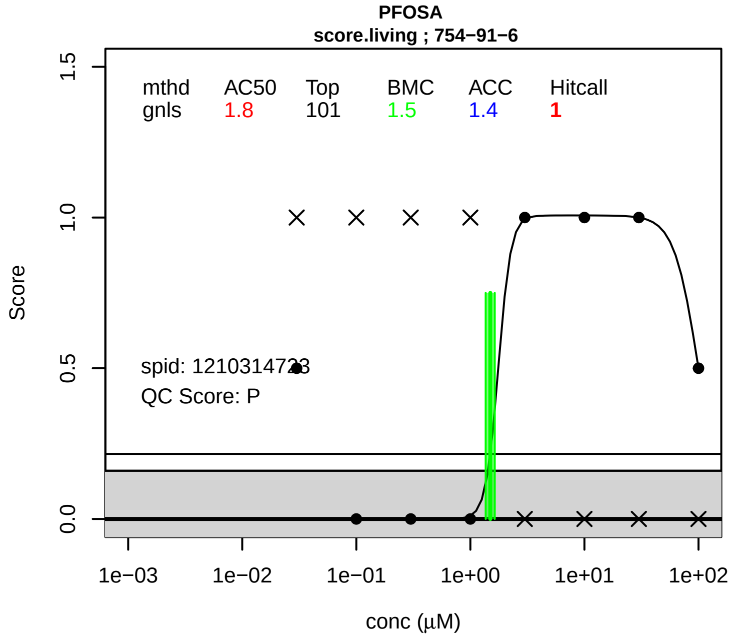
PFOSA
score.living ; 754−91−6
mthd AC50 Top BMC ACC Hitcall
gnls 1.8 101 1.5 1.4 1
QC Score: P
Score
conc (µM)
1e−03 1e−02 1e−01 1e+00 1e+01 1e+02
0.0 0.5 1.0 1.5

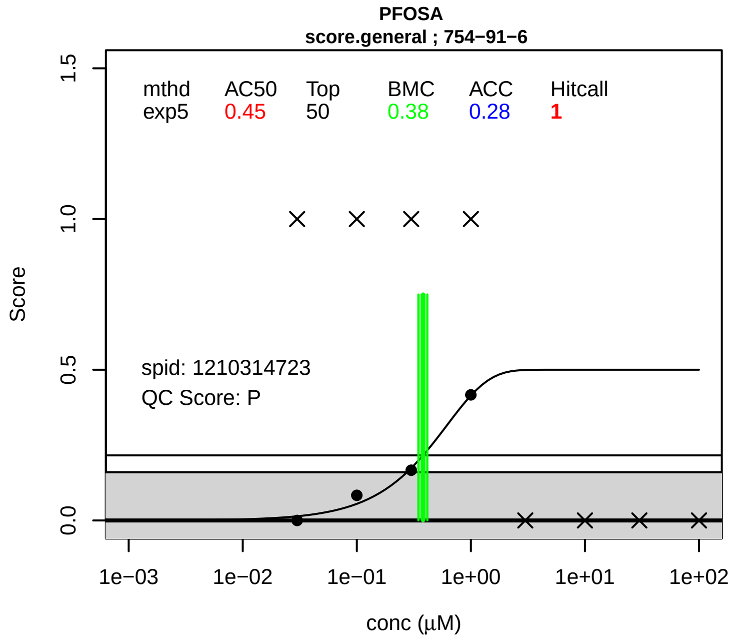
PFOSA
score.general ; 754−91−6
mthd AC50 Top BMC ACC Hitcall
exp5 0.45 50 0.38 0.28 1
spid: 1210314723
QC Score: P
Score
conc (µM)
1e−03 1e−02 1e−01 1e+00 1e+01 1e+02
0.0 0.5 1.0 1.5

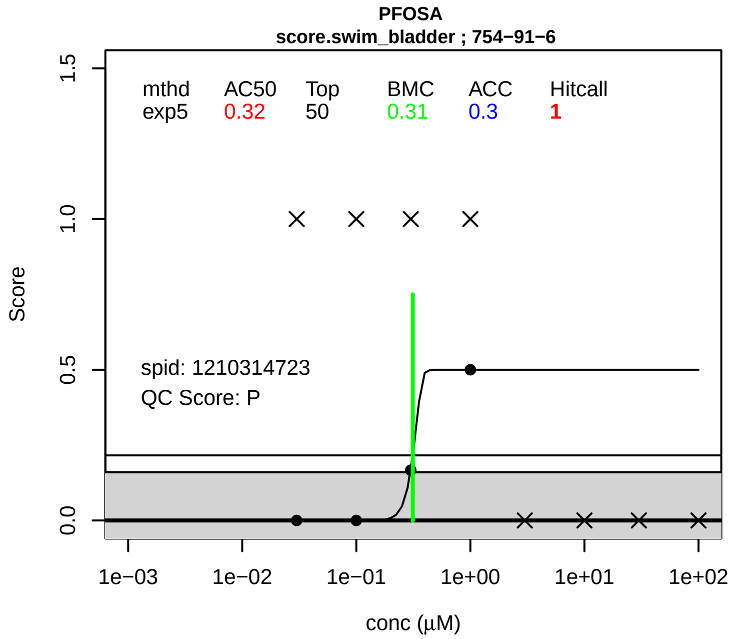
PFOSA
score.swim_bladder ; 754−91−6
mthd AC50 Top BMC ACC Hitcall
exp5 0.32 50 0.31 0.3 1
spid: 1210314723
QC Score: P
Score
conc (µM)
1e−03 1e−02 1e−01 1e+00 1e+01 1e+02
0.0 0.5 1.0 1.5

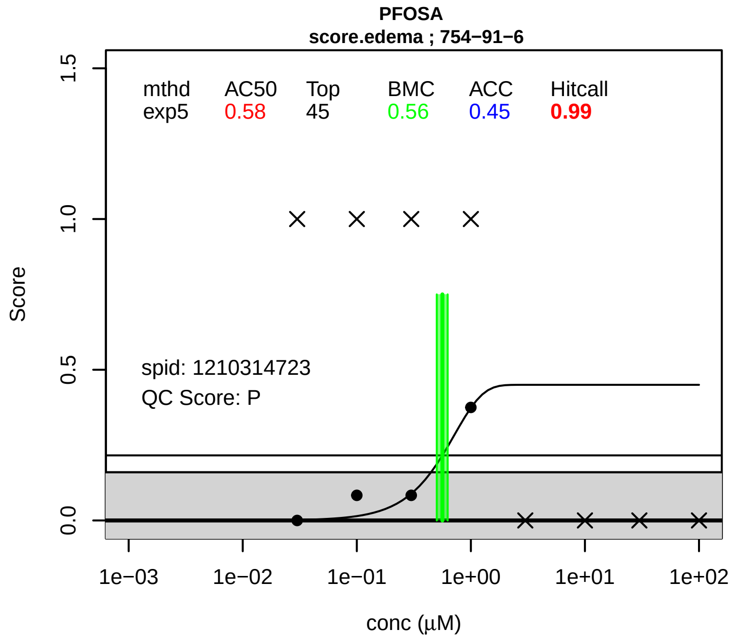
PFOSA
score.edema ; 754−91−6
mthd AC50 Top BMC ACC Hitcall
exp5 0.58 45 0.56 0.45 0.99
spid: 1210314723
QC Score: P
Score
conc (µM)
1e−03 1e−02 1e−01 1e+00 1e+01 1e+02
0.0 0.5 1.0 1.5

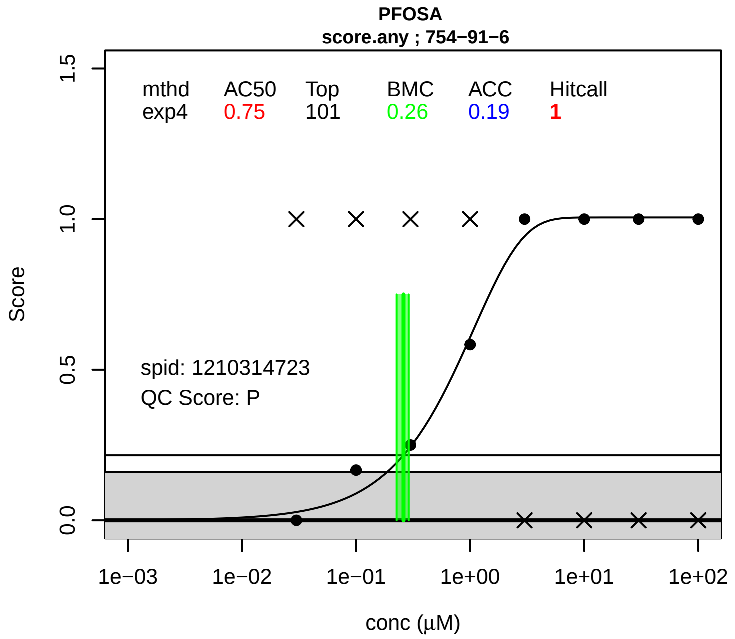
PFOSA
score.any ; 754−91−6
mthd AC50 Top BMC ACC Hitcall
exp4 0.75 101 0.26 0.19 1
spid: 1210314723
QC Score: P
Score
conc (µM)
1e−03 1e−02 1e−01 1e+00 1e+01 1e+02
0.0 0.5 1.0 1.5

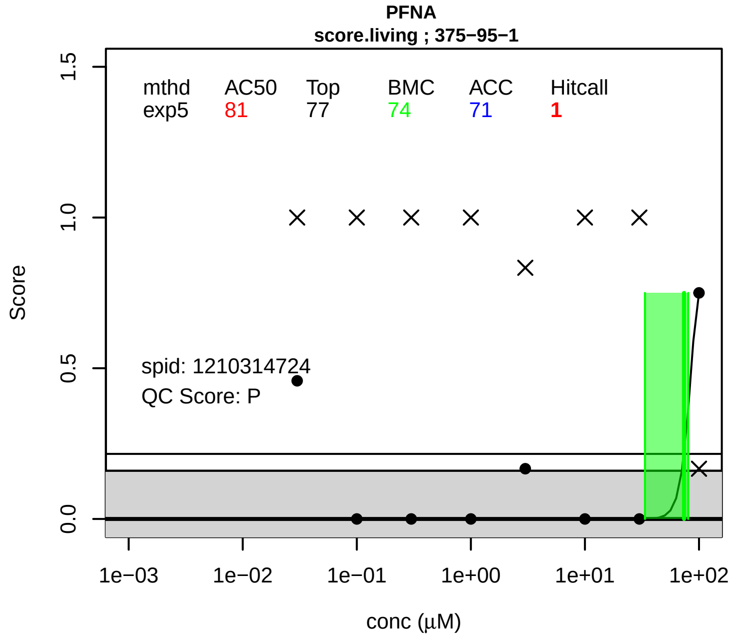
PFNA
score.living ; 375−95−1
mthd AC50 Top BMC ACC Hitcall
exp5 81 77 74 71 1
spid: 1210314724
QC Score: P
Score
conc (µM)
1e−03 1e−02 1e−01 1e+00 1e+01 1e+02
0.0 0.5 1.0 1.5

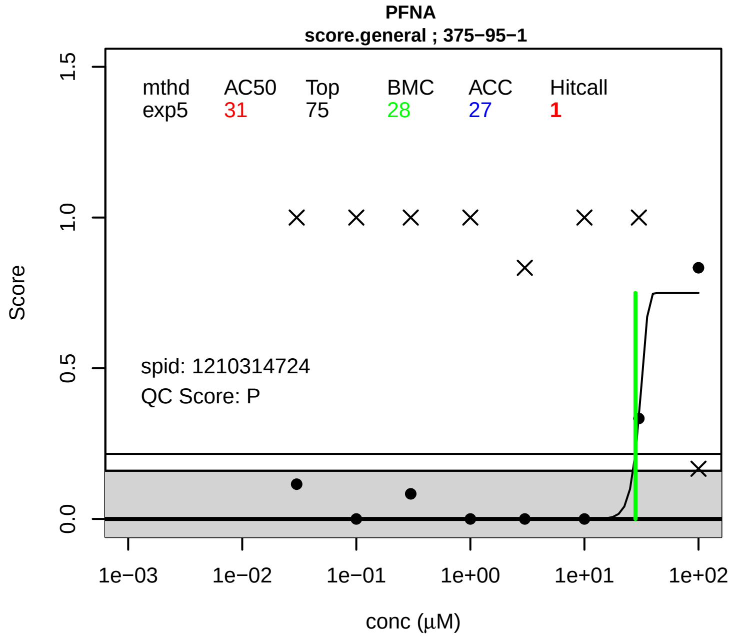
PFNA
score.general ; 375−95−1
mthd AC50 Top BMC ACC Hitcall
exp5 31 75 28 27 1
spid: 1210314724
QC Score: P
Score
conc (µM)
1e−03 1e−02 1e−01 1e+00 1e+01 1e+02
0.0 0.5 1.0 1.5

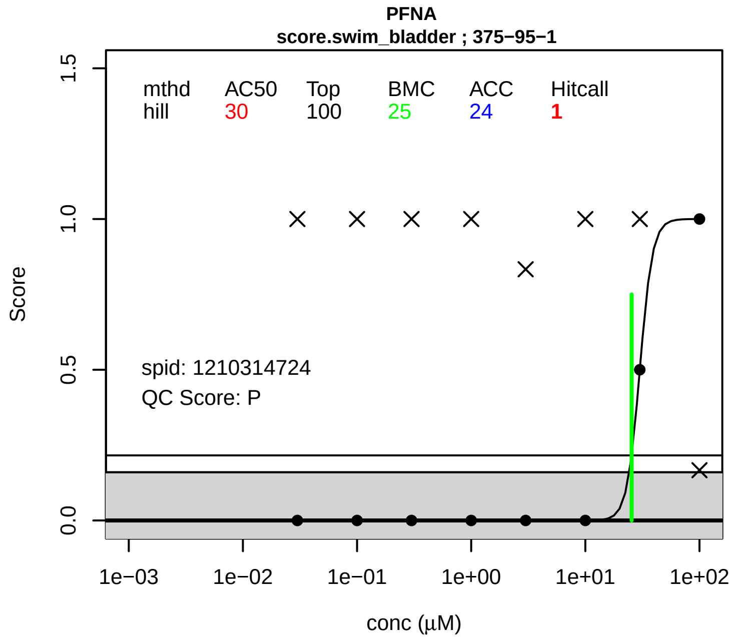
PFNA
score.swim_bladder ; 375−95−1
mthd hill
AC50 30
Top 100
BMC 25
ACC 24
Hitcall 1
spid: 1210314724
QC Score: P
Score
0.0 0.5 1.0 1.5
1e−03 1e−02 1e−01 1e+00 1e+01 1e+02
conc (µM)

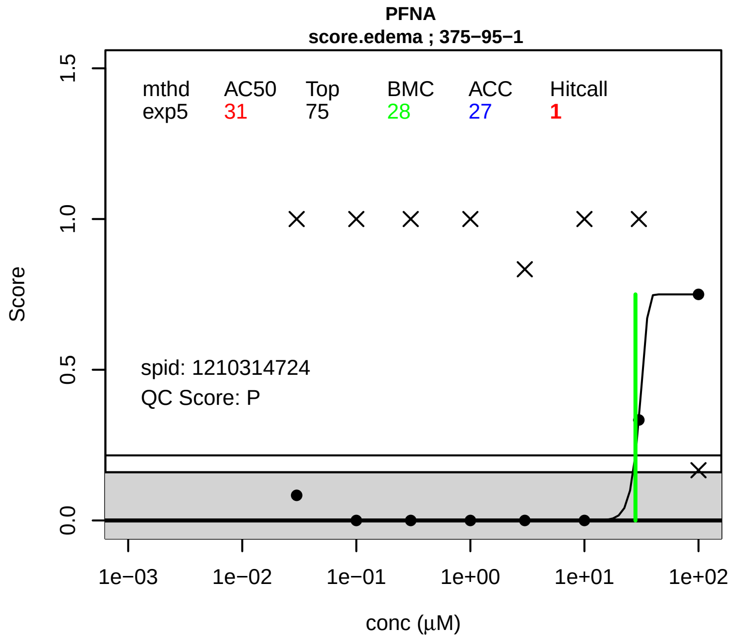
PFNA
score.edema ; 375−95−1
mthd exp5
AC50 31
Top 75
BMC 28
ACC 27
Hitcall 1
spid: 1210314724
QC Score: P
Score
0.0 0.5 1.0 1.5
1e−03 1e−02 1e−01 1e+00 1e+01 1e+02
conc (µM)

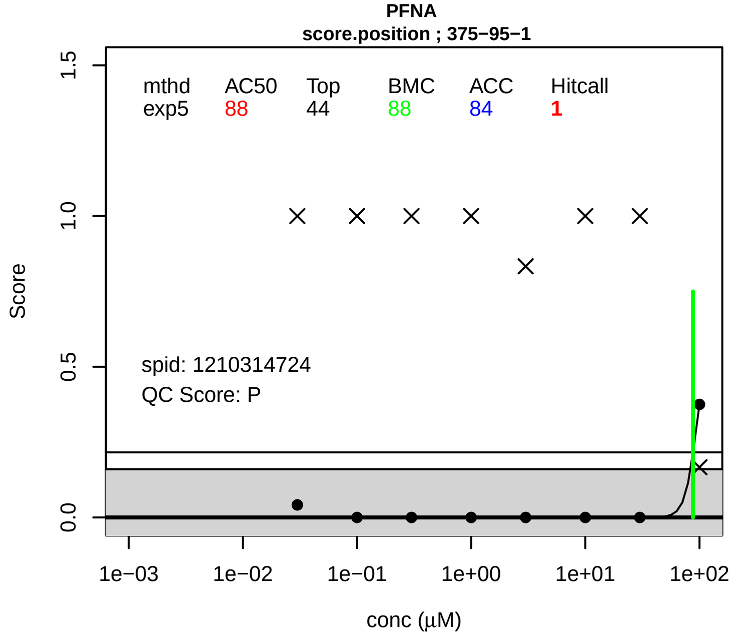
PFNA
score.position ; 375−95−1
mthd exp5
AC50 88
Top 44
BMC 88
ACC 84
Hitcall 1
spid: 1210314724
QC Score: P
Score
0.0 0.5 1.0 1.5
1e−03 1e−02 1e−01 1e+00 1e+01 1e+02
conc (µM)

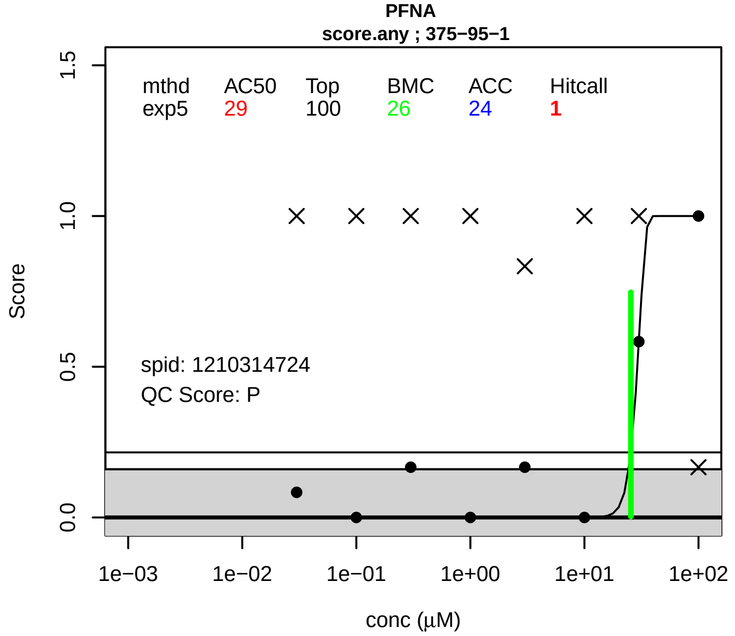
PFNA
score.any ; 375−95−1
mthd exp5
AC50 29
Top 100
BMC 26
ACC 24
Hitcall 1
spid: 1210314724
QC Score: P
Score
0.0 0.5 1.0 1.5
1e−03 1e−02 1e−01 1e+00 1e+01 1e+02
conc (µM)
